# The Core Rehabilitation Outcome Set for Single-Sided Deafness (CROSSSD) study: International consensus on outcome measures for trials of interventions for adults with single-sided deafness

**DOI:** 10.1186/s13063-022-06702-1

**Published:** 2022-09-08

**Authors:** Roulla Katiri, Deborah A. Hall, Derek J. Hoare, Kathryn Fackrell, Adele Horobin, Nicholas Hogan, Nóra Buggy, Paul H. Van de Heyning, Jill B. Firszt, Iain A. Bruce, Pádraig T. Kitterick, for the Core Rehabilitation Outcome Set for Single-Sided Deafness (CROSSSD) initiative, Ad Snik, Carly Sygrove, Cherith Campbell-Bell, Christopher Parker, Daniel M. Zeitler, Lewis Williams, Maxine Oxford, Patrick Boyle, Paul K. James, Penelope R. Hill-Feltham, Peter Toth, Richard Bowles, Richard Nicholson, Roger Bayston, Tove Rosenbom

**Affiliations:** 1 Hearing Sciences, Mental Health and Clinical Neurosciences, School of Medicine, University of Nottingham, Nottingham, NG7 2UH UK; 2 National Institute for Health Research Nottingham Biomedical Research Centre, Ropewalk House, 113 The Ropewalk, Nottingham, NG1 5DU UK; 3 Audiology Department, Mater Misericordiae University Hospital, North Circular Road, Dublin, D07 R2WY Ireland; 4 Department of Psychology, School of Social Sciences, Heriot-Watt University Malaysia, Putrajaya, Malaysia; 5 Wessex Institute, University of Southampton, University Road, Southampton, SO17 1BJ UK; 6 Nottingham University Hospitals NHS Trust, Queen’s Medical Centre, Derby Road, Nottingham, NG7 2UH UK; 7 Department of Otorhinolaryngology, Head and Neck Surgery, Antwerp University Hospital (UZA), 2650, Edegem, Antwerp, Belgium; 8 Experimental Laboratory of Translational Neurosciences, Faculty of Medicine and Health Sciences, University of Antwerp, 2610 Antwerp, Belgium; 9 Washington University School of Medicine, 660 South Euclid Avenue, St. Louis, MO 63110-1010 USA; 10 Manchester University Hospitals NHS Foundation Trust, Manchester Academic Health Science Centre, Oxford Road, Manchester, M13 9WL UK; 11 Division of Infection, Immunity and Respiratory Medicine, Faculty of Biology, Medicine and Health University of Manchester, Oxford Road, Manchester, M13 9PL UK; 12 National Acoustic Laboratories, Australian Hearing Hub, Macquarie University, Sydney, NSW 2109 Australia

**Keywords:** Consensus methods, Outcome domains, Core outcome set, Single-sided deafness, Hearing interventions, Clinical trial design

## Abstract

**Background:**

Single-sided deafness (SSD) has functional, psychological, and social consequences. Interventions for adults with SSD include hearing aids and auditory implants. Benefits and harms (outcome domains) of these interventions are until now reported inconsistently in clinical trials. Inconsistency in reporting outcome measures prevents meaningful comparisons or syntheses of trial results. The Core Rehabilitation Outcome Set for Single-Sided Deafness (CROSSSD) international initiative used structured communication techniques to achieve consensus among healthcare users and professionals working in the field of SSD. The novel contribution is a set of core outcome domains that experts agree are critically important to assess in all clinical trials of SSD interventions.

**Methods:**

A long list of candidate outcome domains compiled from a systematic review and published qualitative data, informed the content of a two-round online Delphi survey. Overall, 308 participants from 29 countries were enrolled. Of those, 233 participants completed both rounds of the survey and scored each outcome domain on a 9-point scale. The set of core outcome domains was finalised via a web-based consensus meeting with 12 participants. Votes involved all stakeholder groups, with an approximate 2:1 ratio of professionals to healthcare users participating in the Delphi survey, and a 1:1 ratio participating in the consensus meeting.

**Results:**

The first round of the survey listed 44 potential outcome domains, organised thematically. A further five outcome domains were included in Round 2 based on participant feedback. The structured voting at round 2 identified 17 candidate outcome domains which were voted on at the consensus meeting. Consensus was reached for a core outcome domain set including three outcome domains: *spatial orientation*, *group conversations in noisy social situations*, and *impact on social situations*. Seventy-seven percent of the remaining Delphi participants agreed with this core outcome domain set.

**Conclusions:**

Adoption of the internationally agreed core outcome domain set would promote consistent assessment and reporting of outcomes that are meaningful and important to all relevant stakeholders. This consistency will in turn enable comparison of outcomes reported across clinical trials comparing SSD interventions in adults and reduce research waste. Further research will determine how those outcome domains should best be measured.

**Supplementary Information:**

The online version contains supplementary material available at 10.1186/s13063-022-06702-1.

## Background

In adults, single-sided deafness (SSD) can lead to a range of well-documented functional hearing difficulties, such as impaired spatial awareness [1, 2] and difficulties perceiving speech in demanding listening environments [3, 4] such as in the presence of background noise [5, 6]. The clinical management of SSD involves a range of different interventions that can broadly be categorised as either rerouting sounds from the deaf ear to the hearing ear, either via air or bone conduction, or by restoring hearing to the deaf ear via a middle ear or cochlear implant. A systematic review of studies evaluating the effectiveness of these SSD interventions identified that outcome selection has been somewhat biased towards assessing functional impairments for which measures are readily available and widely used, e.g., tests of speech perception in noise [7]. However, the difficulties that SSD imposes can also affect the individual’s psychological and social well-being [8, 9], and therefore, outcomes that assess the impact on an individual’s overall health and well-being are also relevant and potentially as important [10]. Healthcare users express uncertainty about choice of treatment options for SSD often due to a lack of clarity about their benefit [11]. A subsequent systematic review identified a total of 520 outcome domains from 96 studies that evaluated SSD interventions [12]. Generally speaking, interventions to reroute sounds (such as contralateral routing of signals (CROS) aid and bone anchored hearing aid (BAHA)) assessed the same outcome domains as restoring interventions (such as middle ear and cochlear implants). However, tinnitus-related outcomes and brain-related assessments of neural activity were almost exclusively limited to studies that evaluated the effect of cochlear implants, while rerouting studies were more concerned about the aversiveness of sounds. With regard to the choice of measurement instruments, the Speech, Spatial and Qualities of hearing scale (SSQ) [13] for example is one of 73 different instruments used to measure speech-related outcomes, but this instrument has also been utilised to assess device benefit, hearing disability, quality of hearing, and spatial hearing [12]. This lack of consistency emphasises the need to define an agreed minimum standard for what is critically important to assess in all clinical trials evaluating SSD interventions. Without such consensus, it will remain challenging to make evidence-based decisions about the relative benefits of the different treatment options.

The need for harmonisation of assessment methods across trials of SSD interventions has already been acknowledged, and recommendations for a minimum set of outcome measures were made following expert clinical professionals panel discussions at two international conferences in 2015 and 2016 [14]. These were daily device use; pure tone audiometry; free-field testing of speech perception in noise and sound localisation; the Speech, Spatial, and Qualities of hearing questionnaire [15]; the Health Utilities Index Mark 3 [16]; and if applicable, the Tinnitus Functional Index [17]. A main limitation of this previous work is that it focussed on cochlear implants as a treatment for SSD and so the expert panels comprised professionals from cochlear implantation centres. Further limitations were considering those outcome measurement instruments that were readily available in the hearing clinic (e.g., pure tone audiometry, standard audiometric and validated sentence test, binaural effect measures) and the lack of healthcare user involvement in the decision-making process. Therefore, it is unclear whether the recommended measures are assessing outcome domains that are most meaningful to healthcare users.

The present harmonisation study addressed these limitations by considering all SSD interventions and all professional experts, by deliberately focusing first on establishing *what* is important to measure, and by giving the healthcare users’ input equal weighting to that of the professionals [18]. It advocates consistent choice of outcomes to ensure high-quality, easily comparable trials that are concentrating on important outcomes relevant to all stakeholders involved. The purpose of the study was to define an agreed minimum standard for what is critically important to assess in all clinical trials evaluating SSD interventions. The expected impact would be to increase the potential for evidence synthesis (i.e., meta-analysis) of published results in order to generate the required evidence base for commissioning of clinical services and for informed decision making between healthcare user and professional.

The study method was informed by a growing body of existing work by a community of core outcome set developers: the Core Outcome Measures in Effectiveness Trials (COMET) initiative [18]. COMET has established minimum standards to guide the process, as well as to help users of core outcome domain sets to evaluate whether they have been developed using appropriate methodology [19]. The COMET initiative has also published a handbook to support the development of consensus-based recommendations for core outcome domains [18]. An agreed minimum standard would not restrict trial investigators from assessing additional outcomes, but rather, it would aim to reduce diversity in reported outcomes and provide a basis for comparison between trials.

## Methods

**Fig. 1:**
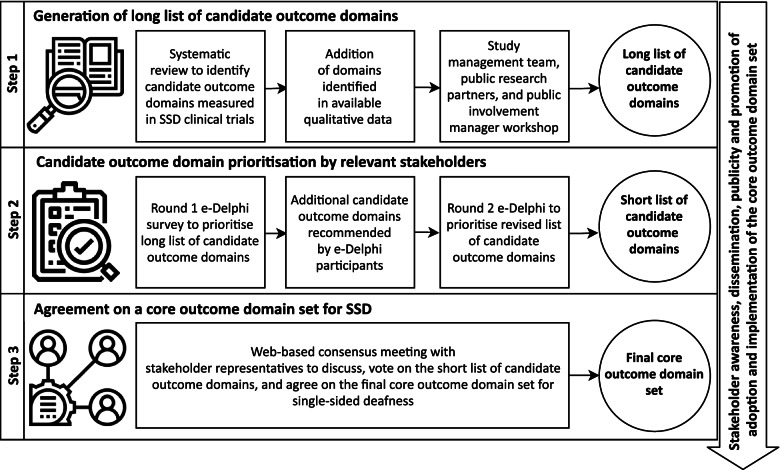
Overview of the process used to develop a core outcome domain set for clinical trials investigating SSD interventions in adults

The full protocol for prioritising the CROSSSD outcome domains has been published [20] in addition to the methodology for conducting the consensus meeting online [21]. The process is outlined in Fig. 1. Our core outcome domain set development process was informed by the COMET Handbook version 1.0 [18] and the Core Outcome Set-STAndards for Development (COS-STAD) [19]. Ethical approval was granted from the Nottingham 2 Research Ethics Committee (Research Ethics Committee reference 19/EM/0222, Integrated Research Application System Project ID 239750) on 06 August 2019. The study is reported according to the Standards for Reporting Qualitative Research (SRQR) [22].

In summary, the study comprised three steps:

- *Step 1:* Generating a long list of candidate outcome domains utilised to date in clinical trials assessing rerouting and restoring interventions for adults with SSD.
- *Step 2:* Prioritising which of these outcome domains are critically important to measure when assessing whether an SSD intervention has worked by involving a large representative set of SSD stakeholders (healthcare users and professionals).
- *Step 3:* Reaching a final consensus decision with a subset of stakeholder representatives on which outcome domains are sufficiently critically important to constitute the core outcome domain set for SSD interventions in adults.

### Changes from protocol

There were no significant deviations to the published protocol [20], except the use of a web-based meeting in place of a face-to-face consensus meeting due to travel and physical distancing restrictions imposed by the coronavirus disease (COVID-19) pandemic [21]. A non-substantial category C amendment to the ethical approval was obtained to accommodate this change (Sponsor reference: 19032, minor amendment reference number: NSA01). Informed consent was obtained from those choosing to participate in the online Delphi (e-Delphi) surveys and prior to participation at the consensus meeting.

### Participants

All relevant groups of stakeholders were invited to participate if they met the following inclusion criteria: (i) healthcare users with lived experience of SSD for 12 months or more, who had received or considered an SSD intervention; (ii) healthcare professionals (e.g., audiologists, ENT surgeons, neuro-otologists) with experience of assessing, diagnosing, or managing SSD in adults; (iii) clinical researchers with recent experience with SSD intervention studies; (iv) commercial representatives that worked for industry partners that developed, manufactured, or sold hearing aids or auditory implants used as SSD interventions; and (v) those employed by organisations that fund research focusing on SSD interventions. Recruitment used a purposive sampling method to engage with qualified experts who had a deep understanding of the topic. Advertisements were targeted at relevant international conferences, known professional bodies, related professional societies and charities, personal contacts of the study steering group, UK-based hearing clinics, and more generally through social media groups. Communications by charities and UK-based hearing clinics were the main routes for healthcare user recruitment. For more details, see Katiri et al., [20]. All participants were required to be at least 18 years old and able to read, understand, and complete web-based surveys in English.

### e-Delphi surveys

The recruitment target was that at least 20 participants in each of the three major stakeholder groups (healthcare users, healthcare professionals, clinical researchers) would complete both rounds of the e-Delphi survey. To minimise attrition between the two survey rounds, the importance of completing both rounds of the survey was emphasised to participants in the information sheets and during Round 1. Our operational definition of completion was for at least 50% of the outcome domains to be scored. This criterion ensured that participants contributing to the decision-making were fully engaged in the process and had sufficient expertise to form personal judgements. Each round was open for only a short time (Round 1 for 10.2 weeks and Round 2 for 13.3 weeks), and Round 2 opened the day after the completion of Round 1. Response rates were monitored weekly, and both generic reminder emails and personalised email reminders were sent to late responders to minimise attrition.

A modified Delphi method (steps 1 and 2, Fig. 1) was adopted, presenting participants with a long list of candidate outcome domains, each accompanied by a plain language definition. The list of outcome domains was compiled using information derived from (i) a systematic review of the literature [12], (ii) available qualitative data [9], and (iii) discussion during a workshop that involved members of the study management team, two public research partners (i.e., people with lived experience of SSD), and an expert in patient and public involvement. This workshop group developed the plain language outcome domain definitions, which were further reviewed by the study steering group for clarity of language and description prior to use in the e-Delphi surveys. The study steering group comprised a clinical audiologist, two otolaryngologists (ENT surgeons), two clinical researchers in hearing science, and one clinical researcher in speech and hearing science who had concomitant experience in clinical audiology.

**Fig. 2:**
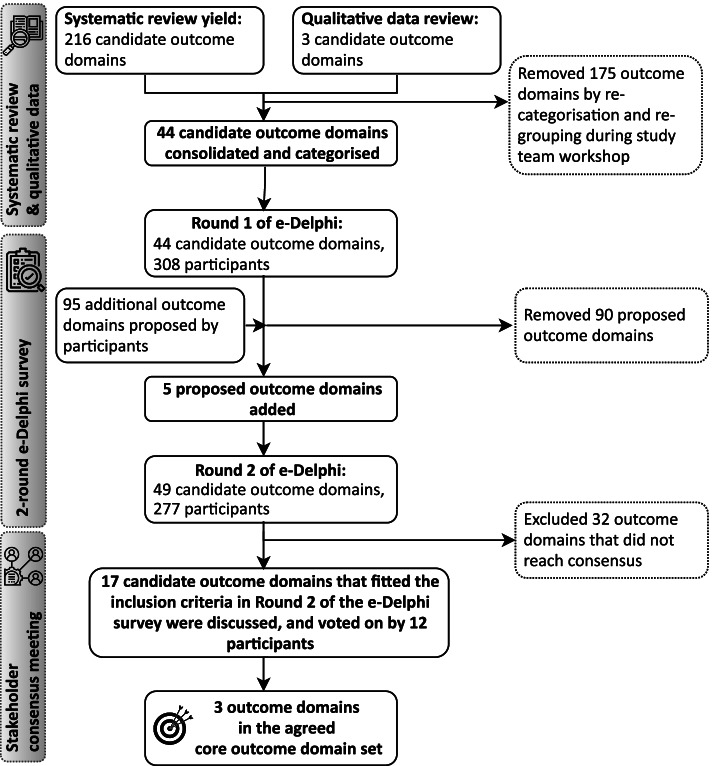
Summary of the steps taken in the outcome domain prioritisation process to agree a final core outcome domain set for clinical trials assessing SSD interventions in adults

The process of identifying and systematically refining the selection of candidate outcome domains is summarised in Figure 2. A total of 433 outcome domains were extracted from studies included in the systematic review [12]. We removed 217 by excluding duplicates and grouping similar outcome domains. The remaining 216 were discussed during the workshop, during which a further 83 domains were removed. Reasons for removing these included (i) the domain was a metric (e.g., thresholds, tonotopy) (*n* = 17), (ii) the domain referred to the measuring instrument (e.g., transcranial attenuation, cortical change) (*n* = 7), (iii) the domain was too generic or vaguely defined (e.g., handicap, cognition) (*n* = 51), and (iv) the domain was device specific (e.g., periodontal, dental measures) (*n* = 8). However, three outcome domains from reviews of the literature on the broader effects of SSD were added because they were deemed relevant [9]. These were personal safety (e.g, road safety, independent living), motivation (e.g., to engage in challenging listening situations), and mood (e.g., general sense of well-being). Following high-level categorisation and further re-grouping, the long list for the e-Delphi survey comprised a total of 44 outcome domains. For ease of presentation to survey participants, these outcome domains were arranged into 10 categories: (1) factors related to the treatment being tested, (2) health-related quality of life, (3) hearing disability, (4) other effects, (5) physical effects, (6) psychological effects, (7) self, (8) sound quality, (9) spatial hearing, and (10) tinnitus. Each outcome domain had a plain language definition explaining in more detail the unique construct it encapsulated (see table, Additional file 1, for list and definitions of initial domains). Most outcome domains described benefits to healthcare users. Category 4 encapsulated any bad or unexpected thing that might happen during the time an SSD treatment is being tested in a clinical trial, i.e., an adverse event.

The 44 outcome domains were presented in both rounds of the e-Delphi survey. At the end of Round 1, participants were invited to propose additional outcome domains. Two members of the study management team and a public research partner reviewed all proposals and five novel outcome domains (device usability, impact on learning, concern about hearing, vulnerability, and independence) were added to Round 2 with a corresponding plain language definition. Outcome domain scoring for the two Delphi rounds was managed using the DelphiManager v4.0 software tool developed and maintained by the COMET initiative at the University of Liverpool. A unique identifier was assigned to each participant linked to their email address, which allowed tracking of participant activity. Outcome domain items were presented in a random order to reduce the potential for systematic contextual effects on scoring, as observed by Brookes et al., [23].

For each outcome domain, participants were asked to consider how important it is to measure when deciding whether an SSD intervention works. Participants scored each outcome domain using the Grading of Recommendations Assessment, Development and Evaluation (GRADE) scale of 1 to 9 [24]. Scoring therefore used a Likert-type scale with additional interpretation categories; 1–3 indicated that the outcome domain was *not important in deciding whether an SSD intervention is effective*, 4–6 indicated that the outcome domain was *important but not critical*, and 7–9 indicated that it was *critically important to measure in all trials of SSD interventions*. Participants were also given an *unable to score* option and could comment on any aspects of the scoring or outcome domains using open-text boxes.

In Round 2, participants were presented with the score they personally gave each outcome domain during Round 1 together with numerical and graphical feedback on the distribution of scores across the key stakeholder groups (healthcare users, healthcare professionals, clinical researchers, or commercial representatives) (see histograms, Additional file 2, for output from Round 2). The protocol set out to consider commercial representatives and funders collectively as one stakeholder group due to similar stakeholder opinions expected and the anticipated small number of participants [20]. However, no funders participated in Round 2. Feedback enabled participants to reflect on their scores in light of the distribution of scores from their own and the rest of the stakeholder groups and re-score each outcome domain if they choose to do so. Consensus was defined as at least 70% of the participants in all three stakeholder groups scoring 7–9 (*critical to measure in all trials*) and fewer than 15% in any stakeholder group scoring 1–3 (*not important in deciding whether an SSD intervention is effective*).

### Consensus meeting

Participants who completed scoring for at least 90% of outcome domains in both rounds of the e-Delphi survey were invited to express their interest to attend the consensus meeting. A balance of 50:50 healthcare users and healthcare professionals was maintained for recruitment. A small number of individuals who had an interest in the process, but did not fit the inclusion criteria to actively participate in the consensus process, joined as observers.

The web-based consensus meeting was 7 h in duration, with three 30-min long breaks, and delivered using Microsoft Teams software. The meeting comprised semi-structured discussions led by three expert facilitators in three small groups (group A, group B, group C), together with large group discussions and voting involving all 12 participants (6 healthcare users and 6 healthcare professionals). The voting participants’ demographics and expertise can be found in Table 1. The two public research partners and the patient and public involvement expert were also present and could take part in the discussions but not vote. All participants were given equal turns to voice their opinions.

**Table 1:** Consensus meeting voting participants demographics and expertise

	Participant expertise	Gender	Age range (years)	Country	SSD expertise	SSD intervention experience
**Group A**	Healthcare user: sudden onset loss	Female	30–39	Spain	3 years	CROS aid
	Healthcare user: acoustic neuroma	Male	70–79	England	28 years	CROS aid
	Audiologist and clinical researcher	Male	60–69	Netherlands	32 years	CROS aids/BAHAs cochlear implants
	Audiologist and clinical researcher	Female	40–49	England	13 years	CROS aids/BAHAs/middle ear implants
**Group B**	Healthcare user: acoustic neuroma	Male	70–79	England	18 years, 10 months	CROS aid
	Healthcare user: sudden onset loss	Male	60–69	England	3 years, 9 months	CROS aid
	Audiologist	Male	50–59	England	35 years	CROS aids
	Clinical researcher and commercial representative	Female	30–39	England	10 years	BAHAs/middle ear implants/cochlear implants
**Group C**	Healthcare user: sudden onset loss	Male	18–29	England	1 year, 1 month	CROS aid/BAHA/cochlear implant
	Healthcare user: childhood loss	Male	70–79	England	73 years	CROS aid
	Clinical researcher and commercial representative	Male	50–59	England	35 years	Cochlear implants
	Audiologist	Male	40–49	Germany	25 years	CROS aids/BAHAs/cochlear implants

In advance of the consensus meeting, voting participants were sent a link to a short survey asking them to consider the outcome domains that had reached the criteria for inclusion in the core outcome domain set after the two rounds of the e-Delphi survey and to vote whether or not they agreed to limit the scope of the consensus meeting to discussing only those outcomes. Anonymised voting was performed using online surveys in real time during the workshop, which asked participants to vote *agree*, *disagree*, or *unsure* in response to questions about the inclusion or exclusion of specific outcome domains. Only outcome domains voted in by at least 70% of participants were included in the core outcome domain set. In cases where a majority vote was not achieved, outcome domains were set aside for re-discussion and re-voting. The results of voting were presented using histograms embedded in PowerPoint slides shared with all participants using Microsoft Teams. All consensus meeting discussions were recorded.

The meeting plans were revised to comply with the travel and physical distancing restrictions imposed by the UK government in 2020, in response to the COVID-19 pandemic. To minimise screen time during the web-based consensus meeting, participants were sent a pre-recorded introductory presentation in advance (see video, Additional file 3, for the introductory presentation) describing the aims of the day, and a guidance document outlining the day’s activities (see text file, Additional file 4, for the meeting plan and guidance document). Participant consent was obtained online.

## Results

### Participants

Of the 308 participants who completed Round 1, 92 (29.9%) were healthcare users, 148 (48.1%) were healthcare professionals, and 59 (19.2%) were clinical researchers (see Additional file 5: Table S5a, for participant numbers). Thirty-one participants (11 healthcare users, 13 healthcare professionals, 6 clinical researchers, 1 funder) rated fewer than 50% of outcome domains in Round 1 so were excluded from Round 2. Retention rate for all stakeholder groups exceeded 85%, with the exception of clinical researchers (74%) and funders (0%) (see Additional file 5: Table S5a, for participant numbers). Most of the consenting participants (*n* = 98, 31.8%) were in the 30–49 age range, followed by the 40–49 years group (*n* = 77, 25.0%). Only healthcare users (*n* = 14, 15.2%) were aged above 69 years (see Additional file 5: Table S5b, for participant numbers).

**Fig. 3:**
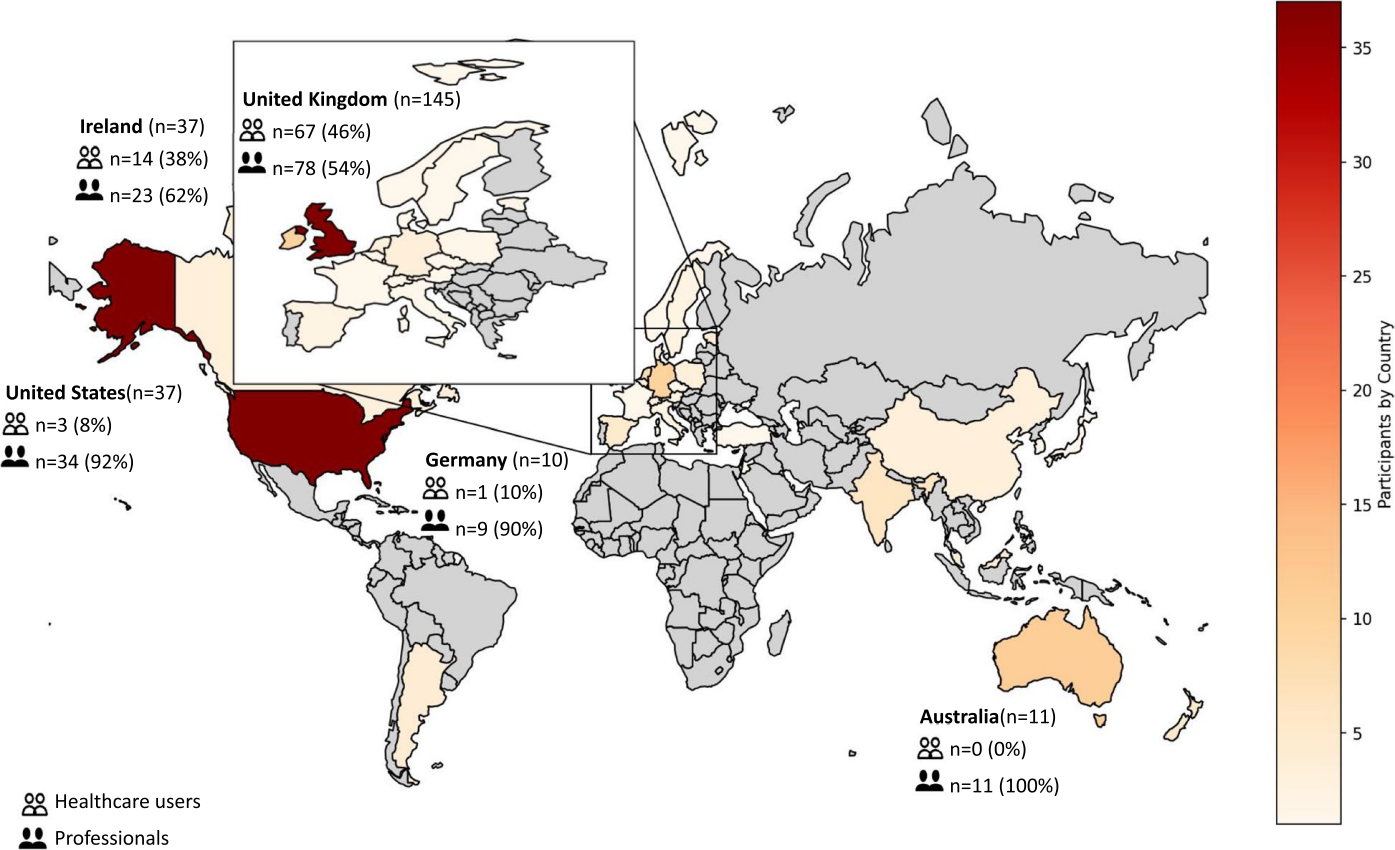
World map illustrating the geographical distribution of all consenting participants (*n* = 308). The number of healthcare users and healthcare professionals for the five countries where most participants were recruited from are also listed

Participants registered from 29 different countries (Fig. 3). The majority were from the UK (*n* = 145, 47.1%), followed by Ireland and the US (*n* = 37, 12.0% from both) (see Additional file 5: Table S5c, for a detailed breakdown of participant registrations in each stakeholder group per country; and Table S5d for a detailed breakdown of their language for everyday communication).

Of the healthcare professionals that reported their roles (*n* = 146), the majority were audiologists or clinical scientists in audiology (*n* = 107, 73.3%), or otolaryngologists/ENT surgeons (*n* = 11, 7.5%).

Of those healthcare users (*n*=84, 91.3%) who disclosed the time since their diagnosis of SSD, most had a history of SSD for 2–5 years (*n*=18, 21.4%), followed by 5–10 years or 10–20 years (*n* = 16, 19.0% in both cases) (see Additional file 5: Figure S5e, for details on the time since SSD diagnosis as disclosed by healthcare users). Almost all (*n* = 81, 88.0%) healthcare users disclosed the devices they had trialled or were primarily using. The majority (*n* = 47, 58.0%) had trialled or were using a CROS device, and a minority (*n* = 9, 11.1%) trialled a BAHA. One participant (1.2%) had received a cochlear implant. Twelve participants (14.8%) had trialled two devices (CROS and BAHA). Three healthcare users (3.7%) reported that they had trialled three devices, including a combination of CROS, BAHA, remote microphone technology, middle ear, or cochlear implants. When asked what interventions they considered trialling, 60 healthcare users (65.2%) provided a response. Most (*n* = 23, 38.3%) had considered a CROS aid, 10 (16.7%) had considered a BAHA, and one (1.7%) had considered the SoundBite^TM^ (Newport Beach, California). Five participants considered trialling restoring interventions, cochlear implantation (*n* = 3, 5.0%), and middle ear implants (*n* = 2, 3.3%). The remaining 18 participants (30.0%) indicated that they considered trialling a combination of two or more re-routing and/or restoring interventions including CROS, BAHA, the SoundBite^TM^, middle ear implants, or cochlear implantation. Three (5.0%) participants commented that they were not aware of alternative options they had to consider.

### e-Delphi surveys

Three hundred and eight participants completed e-Delphi Round 1 (see table, Additional file 2, for the ratings for the 44 outcome domains). Of those, 54 participants submitted 95 comments about potential additional outcome domains that they felt should be included in the list (see table, Additional file 6, for the list of comments provided in Round 1). Most comments (*n* = 43, 45.3%) were from healthcare users and healthcare professionals (*n* = 35, 36.8%), and the rest from clinical researchers (*n* = 16, 16.8%) and commercial representatives (*n* = 1, 1.0%).

Fifty-six of the proposed additional outcome domains were already captured by one or more of the existing domains. Twenty-four comments were rejected as they were out of scope (e.g., concerned economic factors, auditory training, access to hearing loss support groups). Following discussion and feedback from the public research partners and the study steering group, a further nine proposed additional outcome domains were rejected as less relevant. These included aspects of trial device usage, aetiology of the SSD, stigma, sound effects of the device, access to audiological services for timely device adjustment, family support, and availability of auditory implants in different healthcare systems. Three comments (ability to manage treatment option, ease of use, complexity of the device) seemed to be referring to the same concept. Hence, five additional outcome domains were included in Round 2. These were device usability, impact on learning, concern about your hearing, vulnerability, and independence. Two members of the study management team categorised and assigned plain language definitions to these five outcome domains prior to launching Round 2 of the e-Delphi survey. The plain language definitions and categories for these outcome domains can be found in Additional file 1.

Two-hundred and thirty-three participants completed Round 2 (see histograms, Additional file 2, for the ratings for the 49 outcome domains included in Round 2). A number of scores changed between Rounds 1 and 2, such that the outcome domain reached consensus at Round 2 but not at Round 1. Most of these changes were made by the clinical researcher and commercial representative groups with many comments indicating that scores were changed after reviewing the healthcare user group responses. Six outcome domains (device usage, discomfort in listening situations, group conversation in quiet, listening in reverberant conditions, physical tiredness, and spatial orientation) changed for two stakeholder groups. Nine outcome domains (adverse events, being aware of a sound, dissatisfaction with life, emotional distress, mood, motivation, personal safety, and protecting your hearing) changed for one stakeholder group. Two tinnitus-related outcome domains which reached consensus within the commercial representatives at Round 1 did not reach consensus at Round 2 when ratings from the healthcare users were considered. These were loudness and tinnitus-related brain changes. Finally, at Round 1, none of the four stakeholder groups reached consensus to include device malfunction, but all four groups did so at Round 2.

Overall, from Round 2, stakeholder scoring for 17 outcome domains (Table 2) met the consensus criterion for inclusion (see table, Additional file 1, for more details on ratings in Round 2). These were taken forward to the consensus meeting. Participants did not reach consensus on the importance of the remaining 32 outcome domains. These included adverse events which was the only harms outcome. Adverse events was scored 7–9 by 51% of healthcare users, 59% of healthcare professionals, 81% of clinical researchers, and 100% of commercial representatives. The 32 outcome domains were discussed by the study management group and the consensus meeting facilitators. A decision was taken to remove these from the consensus meeting discussions as they did not meet the pre-determined criteria for being important and critical for inclusion.

**Table 2:** Voting scores for the 17 outcome domains that met the criterion for inclusion. Bold font denotes the three core outcome domains. For plain language definitions of the outcome domains, see table, Additional file 1

Outcome domain name	e-Delphi Round 2 scoring (number and percentage of participants that scored 7–9 ‘critically important’)				Consensus meeting scoring
	Healthcare users	Healthcare professionals	Clinical researchers	Commercial representatives	
Domain category: factors related to the treatment being tested					
Treatment satisfaction	60 (87%)	105 (91%)	31 (79%)	7 (100%)	92.2% agreed to exclude
Device usage	48 (72%)	93 (81%)	28 (74%)	5 (71%)	25% agreed to include
Device malfunction	46 (72%)	83 (72%)	28 (74%)	5 (71%)	83.3% agreed to exclude
Domain category: health-related quality of life					
Avoiding social situations	61 (85%)	108 (93%)	34 (89%)	7 (100%)	83.3% agreed to exclude
**Impact on social situations**	**63 (89%)**	**109 (96%)**	**32 (84%)**	**7 (100%)**	**100% agreed to include**
Impact on work	50 (76%)	110 (96%)	33 (87%)	7 (100%)	83.3% agreed to exclude
Domain category: hearing disability					
Being aware of a sound	64 (89%)	101 (88%)	32 (80%)	7 (100%)	25% agreed to include
Listening in complex situations	72 (100%)	108 (94%)	37 (93%)	7 (100%)	58% agreed to include
Listening in reverberant conditions	70 (97%)	86 (75%)	29 (73%)	6 (86%)	83.3% agreed to exclude
Group conversation in quiet	55 (76%)	92 (80%)	32 (80%)	6 (86%)	83.3% agreed to exclude
One-to-one conversation in general noise	68 (96%)	103 (90%)	35 (88%)	7 (100%)	25% agreed to include
**Group conversations in noisy social situations**	**71 (100%)**	**105 (91%)**	**35 (88%)**	**6 (86%)**	**83.3% agreed to include**
Domain category: other effects					
Listening effort	60 (83%)	107 (92%)	33 (85%)	7 (100%)	66.7% agreed to include
Domain category: physical effects					
Physical tiredness	56 (79%)	94 (82%)	30 (77%)	7 (100%)	83.3% agreed to exclude
Domain category: self					
Personal safety	56 (79%)	102 (89%)	31 (82%)	7 (100%)	83.3% agreed to exclude
Domain category: spatial hearing					
Sound localisation	66 (92%)	94 (82%)	36 (92%)	7 (100%)	83.3% agreed to exclude
**Spatial orientation**	**62 (86%)**	**86 (75%)**	**33 (85%)**	**6 (86%)**	**100% agreed to include**

### Pre-consensus meeting survey

All 12 consensus meeting participants completed this survey. Ten (83.3%) participants agreed with the recommendation to discuss only the 17 outcome domains that met the consensus criterion for inclusion at the end of the e-Delphi survey process. Only one participant disagreed (8.3%). With regard to their personal choice of ‘top 3’ out of the 17 candidate outcome domains, the two most popular were listening in complex situations and impact on social situations, selected by five participants. Next were sound localisation, personal safety, listening effort, and group conversations in noisy social situations, selected by four participants. Physical tiredness, impact on work, and device malfunction were not chosen by any of the participants. The remaining eight outcome domains were selected by either one or two participants.

### Consensus meeting

**Fig. 4:**
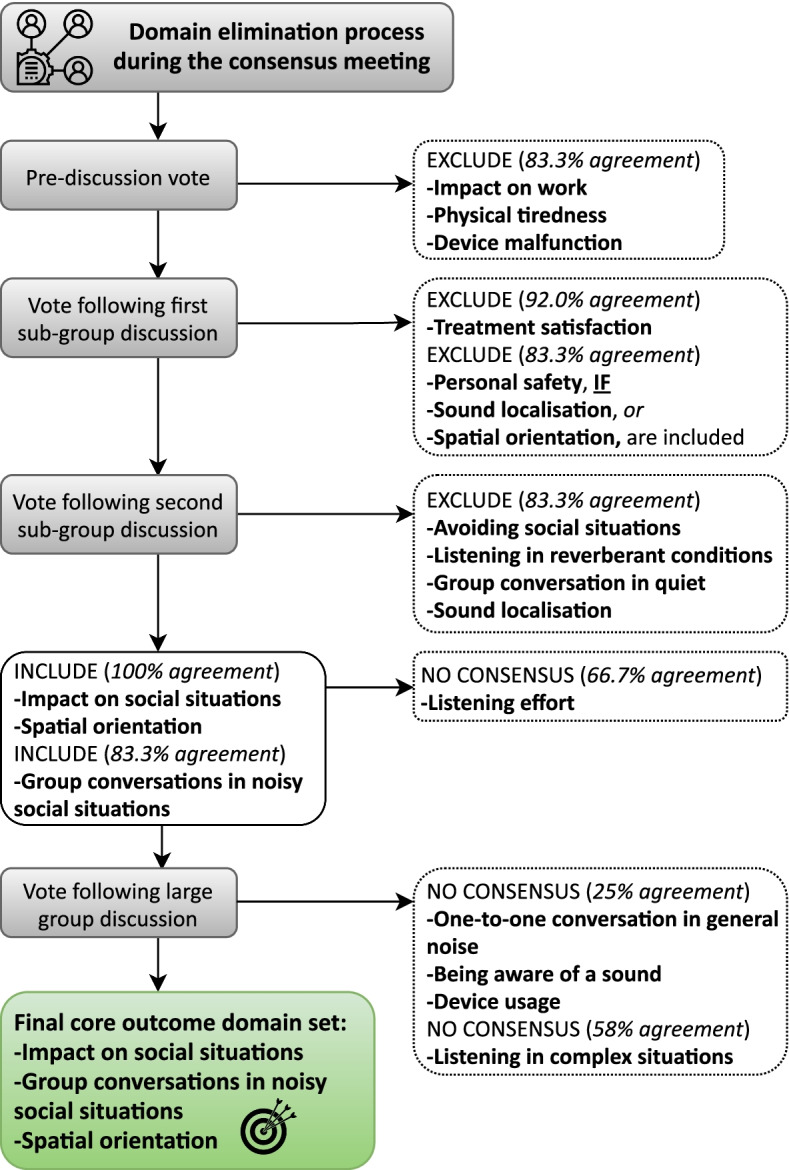
Outcome domain elimination process during the consensus meeting. Only outcome domains voted in by at least 70% of participants were included in the core outcome domain set

Participants first agreed to set aside the outcome domains (83.3% agreement): Physical tiredness, impact on work, and device malfunction. The remaining list of 14 outcome domains were discussed during two small group discussions and subsequently voted on. Initial votes were around whether to exclude outcome domains where a lack of consensus to include them was apparent, and subsequent votes were whether to include remaining outcome domains for inclusion in the core outcome domain set (Fig. 4). Participants agreed that three outcome domains should form the minimum standard. These were impact on social situations (100% agreement), group conversations in noisy social situations (83.3% agreement), and spatial orientation (100% agreement). Supporting comments made during the consensus discussions can be found in Table 3 and there were no comments against their inclusion. Five outcome domains (listening effort, device usage, being aware of a sound, listening in complex situations, and one-to-one conversations in general noise) required more extensive discussion among the group to hear a variety of opinions, but at voting none of these met the consensus criteria for inclusion (Fig. 4).

**Table 3:** Comments in favour of inclusion of these outcome domains, and other general discussion points, as extracted from the consensus meeting discussions

Outcome domain	Participants comments
Impact on social situations	● “Thoroughly covers quite a few other outcome domains, it encapsulates social situations and captures the positives as well as negatives which is important according to the groups’ discussions. Quite a few things can be captured with a single measure” ● “The social situations outcome domain covers whether someone knows when to stop talking and all of this is captured within this domain of social situations” ● “This outcome domain covers ‘Listening effort’ too” ● “Definition relates particularly to situations where a lot of effort is required, effort is a key part of the definition, there is an overlap between ‘Listening effort’ and ‘Impact on social situations’, therefore ‘Listening effort’ was not identified as a domain to be in [the core outcome domain set] on its own right” ● “For me it is about the social situations, it is about family, friends, relationships, when having a few pints down the pub, for me as someone with SSD is about the social side of things”
Group conversations in noisy social situations	● “Provides a good real world example of complex listening and where people with SSD generally have a challenge” ● “One of the hardest speech related tasks so it’s an appropriate outcome measure, and in particular thinking about the devices, e.g., a cochlear implant has a speech processor, it promotes better speech comprehension” ● “This outcome domain captures ‘Listening in complex listening situations’ too”
Spatial orientation	● “Covers more than ‘Sound localisation’, more about the person, more valid: knowing which direction sounds is coming at you from. ‘Sound localisation’ is captured in orientation” ● “More valid for real world situations e.g., car in the street, walking across the road, and covers ‘Sound localisation’ as well” ● “Good fit because the definition includes a safety aspect to it, because it’s about where you are in the world, that is an important aspect of spatial orientation” ● “Covers outcome domain ‘Being aware of a sound’”

### Participant feedback

The final core outcome domain set was shared with the 219 participants (64 healthcare users, 113 healthcare professionals, 36 clinical researchers, six commercial representatives) who completed both rounds of the e-Delphi survey but did not join the consensus meeting. Ninety-five (43.4%) participants responded of whom 32 (50.0%) were healthcare users, 48 (42.5%) were healthcare professionals, 14 (38.9%) were clinical researchers, and one (16.7%) was a commercial representative. Overall, 73 participants (76.8%) responded that they were very satisfied with the choice of included outcome domains, and 19 (20.0%) indicated that they were somewhat satisfied. One participant (healthcare professional) was neither satisfied nor dissatisfied. Two participants (2.1%) responded that they were very dissatisfied; one was a healthcare user that commented that the batteries are too expensive, and the device was unsuitable for their ear; and the other respondent was a healthcare professional that commented that “the outcome domains chosen is what matters most to patients”. None of the participants indicated that they were “somewhat dissatisfied” (see table, Additional file 7, for a detailed breakdown of the participant feedback).

## Discussion

This study completes the first step in the development of a core outcome set for SSD interventions: reaching agreement on *the what*, i.e., the set of standardised outcomes to measure as a minimum in this clinical area [25]. A large group of stakeholders came to a consensus about those outcome domains that are important to measure in adults with SSD, and three outcome domains were identified by consensus as being critically important to measure in *all* clinical trials for SSD interventions in adults. The core outcome domain set recommends which are the most important outcome domains to measure in order to promote greater consistency across trials. The core outcome domain set development process has also provided detailed information about the importance of a broader set of outcome domains from a diverse and international group of key stakeholders that can inform the selection of primary and secondary outcomes for future trials of SSD interventions more generally.

The current work, in focusing on prioritising outcome domains, is complementary to the previous work to establish a unified testing framework for SSD as recommended by Van de Heyning et al., [14]. That previous work aimed to harmonise assessment methods for treatment options prescribed for SSD and asymmetrical hearing loss in clinical practice and was based on the set of measures that were available, familiar, commonly used in previous studies, and judged to be relevant and appropriate for this clinical population by clinical professionals in otolaryngology and audiology. This approach, focusing on available measures (*the how*), is a pragmatic solution to addressing heterogeneity in the selection of outcome measures that has been observed in interventional studies involving adults with SSD [7]. The CROSSSD study perspective was complementary in that its aim was to identify which outcome domains are critical to measure (*the what*), irrespective of whether measures with which those outcome domains could be assessed already exist or are known to the SSD research community. In doing so, it also engaged a broader group of stakeholders using a consensus methodology that incorporates views of all relevant stakeholders, including healthcare users, healthcare professionals, clinical researchers, and commercial representatives.

Perhaps reassuringly, two of the measures recommended by Van de Heyning et al., [14] (speech in noise testing, and localisation testing) appear to be closely related to two of the outcome domains included in the CROSSSD core outcome domain set (group conversations in noisy social situations, and spatial orientation). The third outcome domain in the CROSSSD core outcome domain set (impact on social situations) also related to quality of life, which was identified more generally as an outcome of interest by Van de Heyning et al. [14], whom recommended the use of the Health Utilities Index Mark 3 (HUI3) questionnaire [16]. The HUI3 is however rarely reported in published SSD intervention studies as a quality of life measure, instead the EuroQol Research Foundation (EuroQol-5D-3L) questionnaire [26], the World Health Organization Quality of Life Short Form Survey (WHOQOL-BREF) [27], and Medical Outcome Study Short Form 36 (SF-36) [28] questionnaire tend to be reported [12]. Furthermore, the suitability of the HUI3 multi-attribute score in detecting health-related quality of life changes in cochlear implant recipients has been questioned [29]. For those aged over 55 years, the authors suggested that hearing loss comorbidities and psychosocial factors compromise its sensitivity to implantation-related changes. Of the other measures recommended by the previous consensus study, device usage was discussed extensively during the CROSSSD consensus meeting but only 25% of the stakeholders voted it to be critically important to measure in all trials, and tinnitus-related outcome domains did not reach consensus at the e-Delphi stage. The former decision may reflect that the importance of Device usage as an outcome can vary depending on the nature of the devices being trialled, e.g., usage of cochlear implants during all waking hours is generally strongly encouraged to promote adaptation to the electrical stimulation [30], whereas effective use of devices like CROS aids may involve being judicious about when and where they are used [31]. The lack of consensus around tinnitus could be attributed to it not being a symptom that is experienced by all healthcare users with SSD and only likely modified by some interventions (e.g., a cochlear implant or middle ear implant). As such, it is therefore not an outcome domain that is equally important and critical to all trials of SSD interventions. For SSD trials in which tinnitus is a main outcome of interest, the core outcome domain set for sound-based interventions for tinnitus could be considered [32].

Although adverse events did not meet the criteria for inclusion, we recognise that it is important to rigorously monitor and report adverse aspects of interventions when synthesising evidence [33]. Every healthcare intervention is associated with a risk of harms that should be balanced against therapeutically beneficial outcomes. Despite this, a systematic review showed that reporting of harms’ data in randomised controlled trials across a range of clinical specialties failed to meet the Consolidated Standards of Reporting Trials (CONSORT) harms criteria [34], and is rarely reported in trials of SSD interventions [7]. There may be a number of reasons for this, including the possibility of a mismatch between investigator-led assessment of harms and the experience of patients, harms might be misreported because they are highly diverse, they might be documented in the trial yet under-reported by investigators or influenced by sponsors, and short-term follow-up might fail to spot long-term harms. In our study, we noted that a majority of clinical researchers recognised the importance of reporting adverse events, but fewer healthcare users and healthcare professionals did so. There is no standard way for handling adverse events during core outcome domain set development and different teams have taken different approaches. Some, like CROSSSD, are purely driven by the consensus process [35–37], whereas others are driven by panel discussions [38]. Others agreed that adverse events should be reported as per good clinical practice guidelines and are thus relevant to all clinical trials so fall outside of the core outcome domain set concept [39]. A similar situation to CROSSSD arose in the development of multiple core outcome domain sets for tinnitus trials, in that all outcome domains related to intervention-related benefits rather than harms [32]. However, the authors were explicit in highlighting the importance of assessing and reporting harms regardless of their inclusion in the core outcome domain set or not. There is no apparent rationale for why an equal emphasis should not also be put on assessment and reporting of harms in trials of SSD interventions, both to promote patient safety, good clinical practice, and adherence to the CONSORT recommendations, and we would advocate that approach.

### Strengths and limitations

The CROSSSD study’s approach to develop a core outcome domain set for SSD interventions in adults, using COMET initiative recommendations [18], proved effective. Effectiveness was demonstrated by low attrition rates at the e-Delphi surveys stage, as well as positive participant feedback. The recommended outcome domains are deemed critically important to measure by *all* relevant stakeholders, including both healthcare users and professionals. The methodological approaches used at all stages of the study ensured that all opinions were considered and the resulting decisions were not biased towards clinical researchers or healthcare professionals’ views. Future steps will concentrate on identifying or rigorously developing instruments that can successfully measure each outcome domain.

Despite efforts to fully represent stakeholders internationally (e.g,. recruitment strategies linking in ENT and audiology global ambassadors, professional bodies and charities, and the CROSSSD steering group representatives), recruitment for low- and middle-income countries was limited. This fact is an acknowledged challenge in core outcome domain set development, and previous studies have recommended that geographical and income-based differences should be considered in outcome prioritisation [40]. In the current study, the use of a web-based e-Delphi approach presented few barriers to participation and specific attention was given to recruiting stakeholders for the web-based consensus meeting from various backgrounds. Given that most published clinical trials of SSD interventions have been conducted in North America, Australia and Europe, and the CROSSSD study is focused on developing a core outcome domain set for clinical trials, the sample of participants involved in developing the core outcome domain set is representative of the geographical regions in which future research on SSD interventions is likely to take place. However, further work that includes identifying and appraising measurement instruments to assess the outcome domains in the core outcome domain set should ensure that accessibility is considered as part of that process.

Although our recruitment strategy sought to engage a diversity of participants, there was a predominance of CROS aid healthcare users, audiology healthcare professionals, and female participants. These reflect real-world imbalances in current clinical practice. With respect to healthcare users, the greater numbers of CROS aid users is not surprising, since this device is the longest standing, non-surgical remediation solution for SSD [41, 42]. With respect to healthcare professionals, in many of the participating countries, audiologists are the first point of call for SSD interventions because they assess, counsel and rehabilitate hearing aid and auditory implant users.

There was no restriction on the minimum number of years of clinical experience of participating healthcare professionals, which might have had an impact on the choice of outcome domains [43]. Variables such as knowledge of outcome measures and having a master’s level qualification can be influencing factors [44]. In the context of core outcome set development, time for reflection and vicarious thinking were important drivers for score changes when choosing outcome measures in Round 2 [45].

Participants commented on the clarity of definitions and choice of language used. Despite involving public research partners from the inception of the current study, as per recommended standards [18], and having the outcome domain definitions reviewed by the study management team and steering group representatives, we observed there was still ambiguity detected by participants during both the e-Delphi surveys and consensus meeting. This challenge was also noted by colleagues [46], who co-produced plain language descriptors and introduced additional examples to their definitions. Other core outcome set developers have translated their surveys into other languages, aiming to capture several culturally diverse regions and optimise global participation [47] but they found very little variation in opinion within stakeholder groups when participant region and other characteristics were considered [48]. Future core outcome domain set developers could seek feedback on the clarity of definitions from a larger number of stakeholder representatives internationally (e.g., via charities or professional bodies) to address any ambiguity.

## Conclusions

The three recommended outcome domains represent what is deemed critically important to always measure *as a minimum*, and clearly fitted our consensus criteria. However, this list does not restrict clinical trialists from assessing other outcomes. Five other outcome domains (listening effort, one-to-one conversation in general noise, being aware of a sound, device usage, and listening in complex situations) reached the final stages of elimination and were considered highly important by stakeholders throughout the process despite not making it into the core outcome domain set. Therefore, special consideration could be placed on these as well as the core outcome domain set domains when designing future clinical trials. Wide adoption of the resulting core outcome domain set in trials investigating SSD interventions will lead to high-quality, easily comparable trials that are concentrating on important outcomes relevant to all stakeholders involved. Moreover, there is an increasing expectation by funders for justification on the choice of outcome measures [49].

Stakeholder awareness, dissemination, publicity, and promotion of adoption and implementation of the core outcome domain set are ongoing. For example, a short video on YouTube aims to raise awareness of the study outcomes [50]. The well-documented problems of research waste [51] will be addressed, unnecessary duplication of effort and outcome-reporting bias will be eliminated, and evidence synthesis in the SSD field will be enhanced if the core outcome domain set is widely adopted. The next steps will concentrate on determining how these outcomes should be operationalised and measured, i.e., the identification or development of robust instruments that can measure the chosen outcome domains. Consideration will also be placed on the instruments’ relevance to clinical practice from the perspective of healthcare users and professionals. Subsequent development of unified testing guidelines that incorporate the core outcome domain set, appropriate measuring instruments, and time-frame of measurement will eliminate the diversity and inconsistency of reported measures in the field of SSD.

## Supplementary Information

**Additional file 1.** Outcome domains and Round 2 e-Delphi survey consensus results

**Additional file 2.** e-Delphi Round 1 and Round 2 outcome domain distribution scores

**Additional file 3.** Pre-recorded introductory presentation for consensus meeting

**Additional file 4.** Detailed plan and guidance document for consensus meeting

**Additional file 5.** Consenting participants’ characteristics and demographics

**Additional file 6.** Suggested additional outcomes at Round 1 e-Delphi with final decisions

**Additional file 7.** Detailed breakdown of the participant feedback

## Data Availability

The datasets used and/or analysed during the current study are available from the corresponding author on reasonable request.

## Abbreviations

BAHA: Bone Anchored Hearing Aid
COMET: Core Outcome Measures in Effectiveness Trials
CONSORT: Consolidated Standards of Reporting Trials
COS-STAD: Core Outcome Set-STAndards for Development
COVID-19: Coronavirus disease
CROS: Contralateral Routing of Signals
CROSSSD: Core Rehabilitation Outcome Set for Single-Sided Deafness
GRADE: Grading of Recommendations Assessment, Development and Evaluation
HUI3: Health Utilities Index Mark 3
SF-36: Medical Outcome Study Short Form 36
SRQR: Standards for Reporting Qualitative Research
SSD: Single-sided deafness
SSQ: Speech, Spatial and Qualities hearing scale
UK: United Kingdom
WHOQOL-BREF: World Health Organization Quality of Life Short Form Survey
US: United States

## References

[1] Akeroyd MA (2006). The psychoacoustics of binaural hearing. Int J Audiol.

[2] Douglas SA, Yeung P, Daudia A, Gatehouse S, O’Donoghue GM (2007). Spatial hearing disability after acoustic neuroma removal. Laryngoscope..

[3] Gallun FJ (2021). Impaired binaural hearing in adults: a selected review of the literature. Front Neurosci.

[4] Snapp HA, Ausili SA (2020). Hearing with one ear: consequences and treatments for profound unilateral hearing loss. J Clin Med.

[5] Hawley ML, Litovsky RY, Culling JF (2004). The benefit of binaural hearing in a cocktail party: effect of location and type of interferer. J Acoust Soc Am.

[6] Welsh LW, Welsh JJ, Rosen LF, Dragonette JE (2004). Functional impairments due to unilateral deafness. Ann Otol Rhinol Laryngol.

[7] Kitterick PT, Smith SN, Lucas L (2016). Hearing instruments for unilateral severe-to-profound sensorineural hearing loss in adults: a systematic review and meta-analysis. Ear Hear.

[8] Carlsson P-I, Hall M, Lind K-J, Danermark B (2011). Quality of life, psychosocial consequences, and audiological rehabilitation after sudden sensorineural hearing loss. Int J Audiol.

[9] Lucas L, Katiri R, Kitterick PT (2018). The psychological and social consequences of single-sided deafness in adulthood. Int J Audiol.

[10] Kitterick PT, Lucas L, Smith SN (2015). Improving health-related quality of life in single-sided deafness: a systematic review and meta-analysis. Audiol Neurootol.

[11] Underdown T, Pryce H (2022). How do patients decide on interventions for single sided deafness? A qualitative investigation of patient views. Int J Audiol.

[12] Katiri R, Hall DA, Killan CF, Smith S, Prayuenyong P, Kitterick PT (2021). Systematic review of outcome domains and instruments used in designs of clinical trials for interventions that seek to restore bilateral and binaural hearing in adults with unilateral severe to profound sensorineural hearing loss ('single-sided deafness’). Trials..

[13] Gatehouse S, Noble W (2004). The speech, spatial and qualities of hearing scale (SSQ). Int J Audiol.

[14] Van de Heyning P, Távora-Vieira D, Mertens G, Van Rompaey V, Rajan GP, Müller J (2017). Towards a unified testing framework for single-sided deafness studies: a consensus paper. Audiol Neurootol.

[15] Noble W, Jensen NS, Naylor G, Bhullar N, Akeroyd MA (2013). A short form of the speech, spatial and qualities of hearing scale suitable for clinical use: the SSQ12. Int J Audiol.

[16] Horsman J, Furlong W, Feeny D, Torrance G (2003). The health utilities index (HUI): concepts, measurement properties and applications. Health Qual Life Outcomes.

[17] Meikle MB, Henry JA, Griest SE, Stewart BJ, Abrams HB, McArdle R (2012). The tinnitus functional index: development of a new clinical measure for chronic, intrusive tinnitus. Ear Hear.

[18] Williamson PR, Altman DG, Bagley H, Barnes KL, Blazeby JM, Brookes ST (2017). The COMET handbook: version 1.0. Trials..

[19] Kirkham JJ, Davis K, Altman DG, Blazeby JM, Clarke M, Tunis S (2017). Core outcome set-STAndards for development: the COS-STAD recommendations. PLoS Med.

[20] Katiri R, Hall DA, Buggy N, Hogan N, Horobin A, Van de Heyning P (2020). Core rehabilitation outcome set for single sided deafness (CROSSSD) study: protocol for an international consensus on outcome measures for single sided deafness interventions using a modified Delphi survey. Trials..

[21] Katiri R, Hall DA, Hoare DJ, Fackrell K, Horobin A, Buggy N (2021). Redesigning a web-based stakeholder consensus meeting about core outcomes for clinical trials: formative feedback study. JMIR Form Res.

[22] O’Brien BC, Harris IB, Beckman TJ, Reed DA, Cook DA (2014). Standards for reporting qualitative research: a synthesis of recommendations. Acad Med.

[23] Brookes ST, Chalmers KA, Avery KNL, Coulman K, Blazeby JM (2018). Impact of question order on prioritisation of outcomes in the development of a core outcome set: a randomised controlled trial. Trials..

[24] Guyatt GH, Oxman AD, Kunz R, Atkins D, Brozek J, Vist G (2011). GRADE guidelines: 2. Framing the question and deciding on important outcomes. J Clin Epidemiol.

[25] Clarke M (2007). Standardising outcomes for clinical trials and systematic reviews. Trials..

[26] EuroQol Group (1990). EuroQol - a new facility for the measurement of health-related quality of life. Health Policy (New York).

[27] Skevington SM, Lotfy M, O’Connell KA (2004). The World Health Organization’s WHOQOL-BREF quality of life assessment: psychometric properties and results of the international field trial. A report from the WHOQOL group. Qual Life Res.

[28] Ware JEJ, Sherbourne CD (1992). The MOS 36-item short-form health survey (SF-36). I. Conceptual framework and item selection. Med Care.

[29] Andries E, Gilles A, Topsakal V, Vanderveken O, Van de Heyning P, Van Rompaey V (2022). The impact of cochlear implantation on health-related quality of life in older adults, measured with the health utilities index mark 2 and mark 3. Eur Arch Otorhinolaryngol.

[30] Alzaher M, Vannson N, Deguine O, Marx M, Barone P, Strelnikov K (2021). Brain plasticity and hearing disorders. Rev Neurol.

[31] Pedley AJ, Kitterick PT (2017). Contralateral routing of signals disrupts monaural level and spectral cues to sound localisation on the horizontal plane. Hear Res.

[32] Hall DA, Smith H, Hibbert A, Colley V, Haider HF, Horobin A (2018). The COMiT’ID study: developing core outcome domains sets for clinical trials of sound-, psychology-, and pharmacology-based interventions for chronic subjective tinnitus in adults. Trends Hear.

[33] Peryer G, Golder S, Junqueira D, Vohra S, Loke YK. Chapter 19: Adverse effects. In: Higgins JPT, Thomas J, Chandler J, Cumpston M, Li T, Page MJ, Welch VA (editors). Cochrane Handbook for Systematic Reviews of Interventions version 6.2 (updated February 2021). Cochrane, 2021. Available from https://training.cochrane.org/handbook/archive/v6.2/chapter-19.

[34] Hodkinson A, Kirkham JJ, Tudur-Smith C, Gamble C (2013). Reporting of harms data in RCTs: a systematic review of empirical assessments against the CONSORT harms extension. BMJ Open.

[35] Allin BSR, Hall NJ, Ross AR, Marven SS, Kurinczuk JJ, Knight M (2019). Development of a gastroschisis core outcome set. Arch Dis Child Fetal Neonatal Ed.

[36] Beuscart J-B, Knol W, Cullinan S, Schneider C, Dalleur O, Boland B (2018). International core outcome set for clinical trials of medication review in multi-morbid older patients with polypharmacy. BMC Med.

[37] Callis Duffin K, Merola JF, Christensen R, Latella J, Garg A, Gottlieb AB (2018). Identifying a core domain set to assess psoriasis in clinical trials. JAMA Dermatol.

[38] Balakrishnan K, Sidell DR, Bauman NM, Bellia-Munzon GF, Boesch RP, Bromwich M (2019). Outcome measures for pediatric laryngotracheal reconstruction: international consensus statement. Laryngoscope..

[39] Haywood K, Whitehead L, Nadkarni VM, Achana F, Beesems S, Böttiger BW (2018). COSCA (Core outcome set for cardiac arrest) in adults: an advisory statement from the international liaison committee on resuscitation. Circulation..

[40] Lee A, Davies A, Young AE (2020). Systematic review of international Delphi surveys for core outcome set development: representation of international patients. BMJ Open.

[41] Harford E, Barry J (1965). A rehabilitative approach to the problem of unilateral hearing impairment: the contralateral routing of signals CROS. J Speech Hear Disord.

[42] Snapp HA (2019). Nonsurgical management of single-sided deafness: contralateral routing of signal. J Neurol Surg B Skull Base.

[43] Hall N, Parker D, Williams A (2020). An exploratory qualitative study of health professional perspectives on clinical outcomes in UK orthotic practice. J Foot Ankle Res.

[44] Copeland JM, Taylor WJ, Dean SG (2008). Factors influencing the use of outcome measures for patients with low back pain: a survey of New Zealand physical therapists. Phys Ther.

[45] Fish R, MacLennan S, Alkhaffaf B, Williamson PR (2020). “Vicarious thinking” was a key driver of score change in Delphi surveys for COS development and is facilitated by feedback of results. J Clin Epidemiol.

[46] Smith H, Horobin A, Fackrell K, Colley V, Thacker B, Hall DA (2018). Defining and evaluating novel procedures for involving patients in core outcome set research: creating a meaningful long list of candidate outcome domains. Res Involv Engag.

[47] Alkhaffaf B, Blazeby JM, Metryka A, Glenny A-M, Adeyeye A, Costa PM (2021). Methods for conducting international Delphi surveys to optimise global participation in core outcome set development: a case study in gastric cancer informed by a comprehensive literature review. Trials..

[48] Alkhaffaf B, Metryka A, Blazeby JM, Glenny A-M, Williamson PR, Bruce IA (2021). How are trial outcomes prioritised by stakeholders from different regions? Analysis of an international Delphi survey to develop a core outcome set in gastric cancer surgery. PLoS One.

[49] Williamson PR, Altman DG, Blazeby JM, Clarke M, Devane D, Gargon E (2012). Developing core outcome sets for clinical trials: issues to consider. Trials..

[50] CROSSSD Study Initiative. (2022). CROSSSD study outcomes YouTube video clip. https://www.youtube.com/watch?v=BcUy_2bzHZw. Accessed 27 Aug 2022.

[51] Chalmers I, Bracken MB, Djulbegovic B, Garattini S, Grant J, Gulmezoglu AM (2014). How to increase value and reduce waste when research priorities are set. Lancet..

