# Supplementary material for: The Core Rehabilitation Outcome Set for Single-Sided Deafness (CROSSSD) study: International consensus on outcome measures for trials of interventions for adults with single-sided deafness
Source: Trials. 2022 Sep 8;23:764. doi: 10.1186/s13063-022-06702-1 (PMC9454406; doi:10.1186/s13063-022-06702-1)
Supplement: Supplementary file 2 — Additional file 2. e-Delphi Round 1 and Round 2 outcome domain distribution scores [file 13063_2022_6702_MOESM2_ESM.pdf]

e-Delphi Round 1 outcome domain rating

CROSSSD

CORE REHABILITATION OUTCOME SET for SINGLE SIDED DEAFNESS

Identifying what is critical and important to measure when evaluating hearing interventions for adults with Single Sided Deafness (SSD)

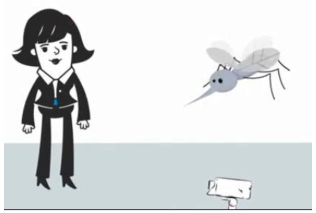

1. AVERSION TO LOUD SOUNDS

Feeling uncomfortable when listening to loud sounds

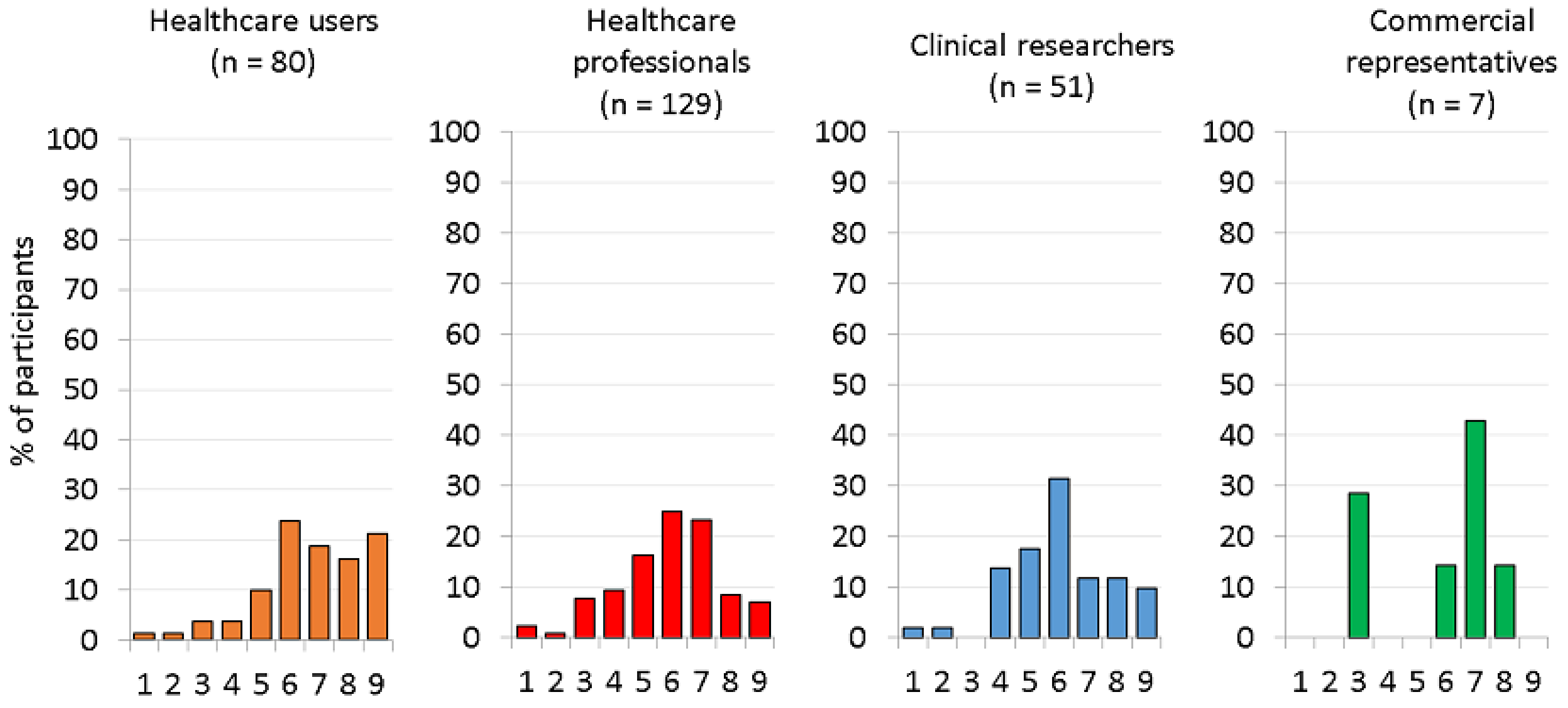

Domain Category:

Psychological effects

NIHR | National Institute for Health Research

Rating scale:

1	2	3	4	5	6	7	8	9
Not at all important			*Important but not critical*			*Critical*		

2. DISCOMFORT IN LISTENING SITUATIONS

Finding yourself in listening situations that you feel you can't adequately control (for example; when you can't choose a favourable listening position); or situations in which you don't feel comfortable (for example when interacting with people who don't know you have a hearing loss)

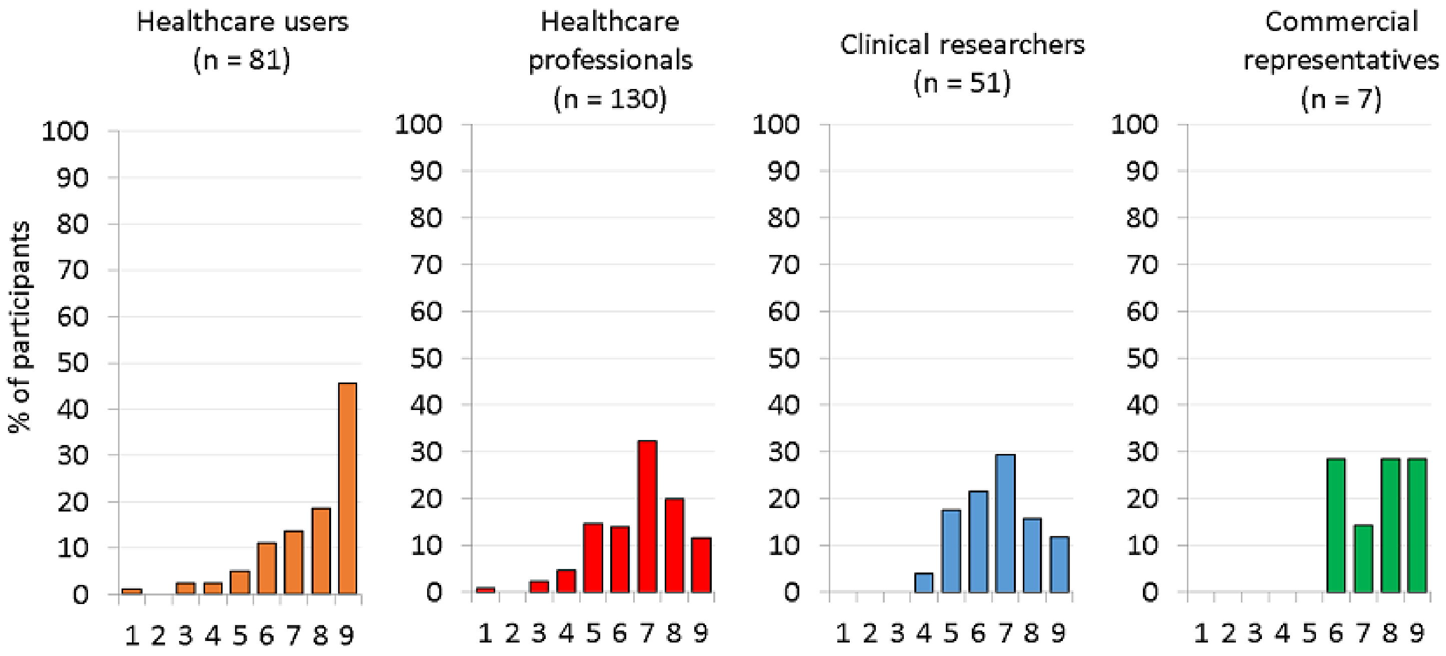

Domain Category:
Psychological effects

NIHR | National Institute for Health Research

Rating scale:

1	2	3	4	5	6	7	8	9
Not at all important			*Important but not critical*			*Critical*		

3. EMOTIONAL DISTRESS

A negative unpleasant emotional reaction which may include fear; anger; frustration; anxiety; and suffering

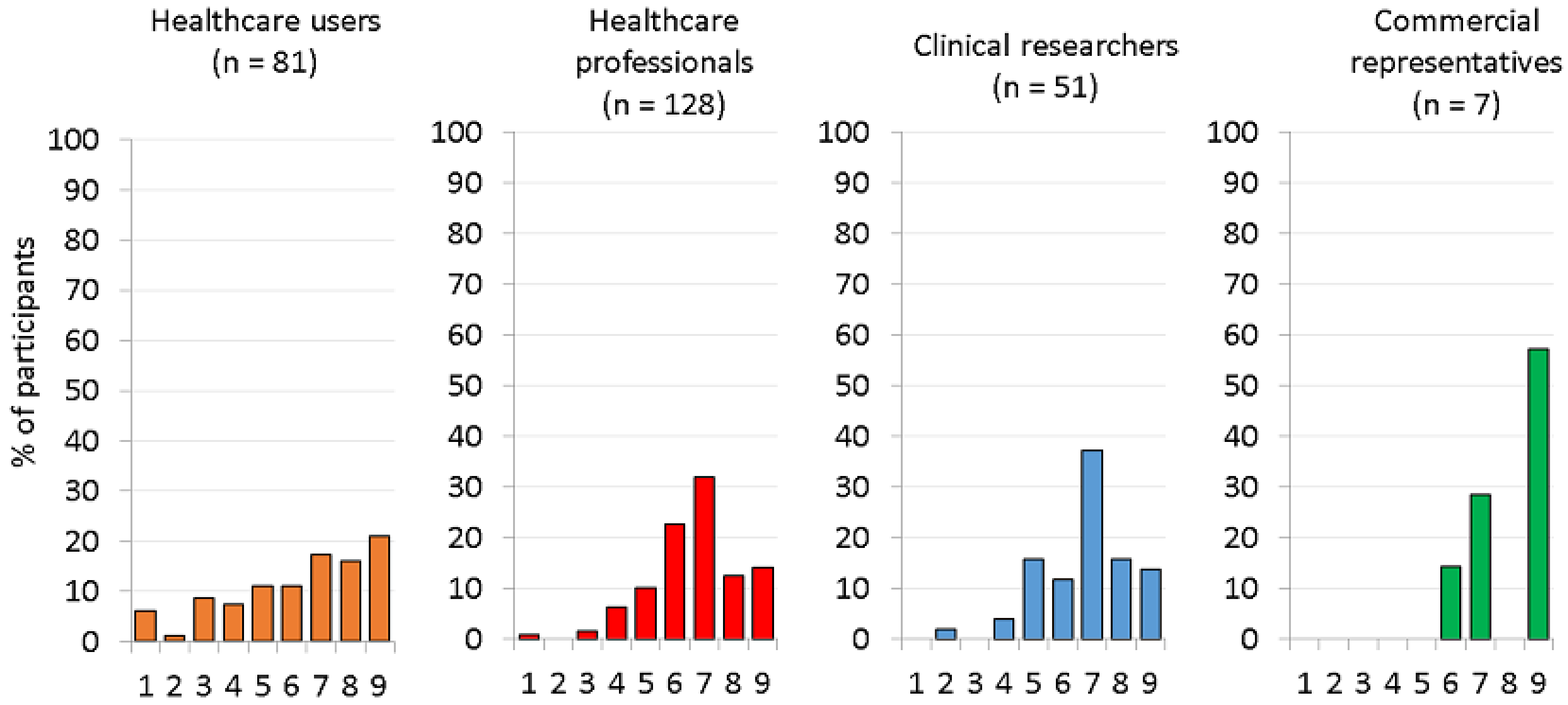

Domain Category:
Psychological effects

NIHR | National Institute for Health Research

Rating scale:

1	2	3	4	5	6	7	8	9
Not at all important			*Important but not critical*			*Critical*		

4. MOOD

General sense of well-being; ranging from feeling very low or negative to very positive

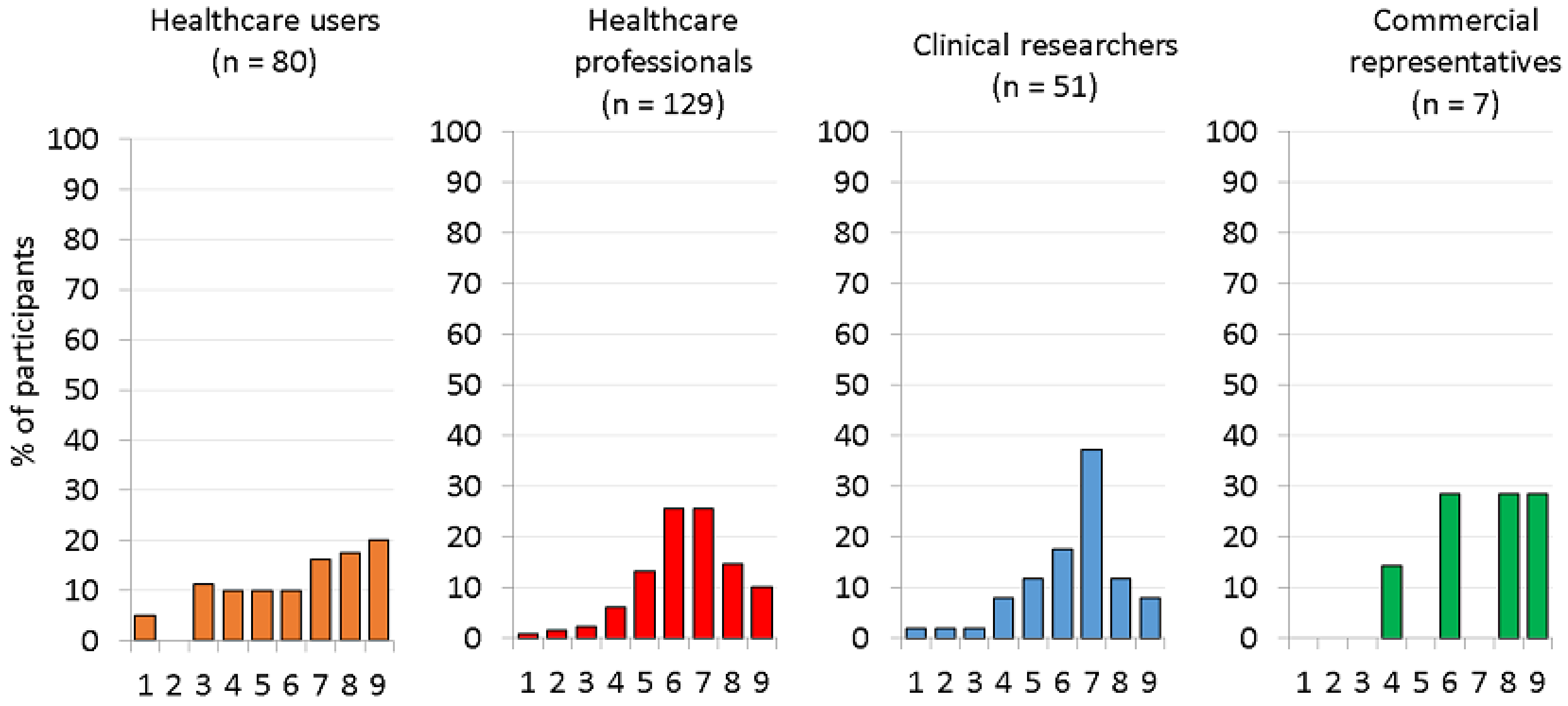

Domain Category:
Psychological effects

NIHR | National Institute for Health Research

Rating scale:

1	2	3	4	5	6	7	8	9
Not at all important			*Important but not critical*			*Critical*		

5. MOTIVATION

A willingness to engage in challenging listening situations

CROSSSD

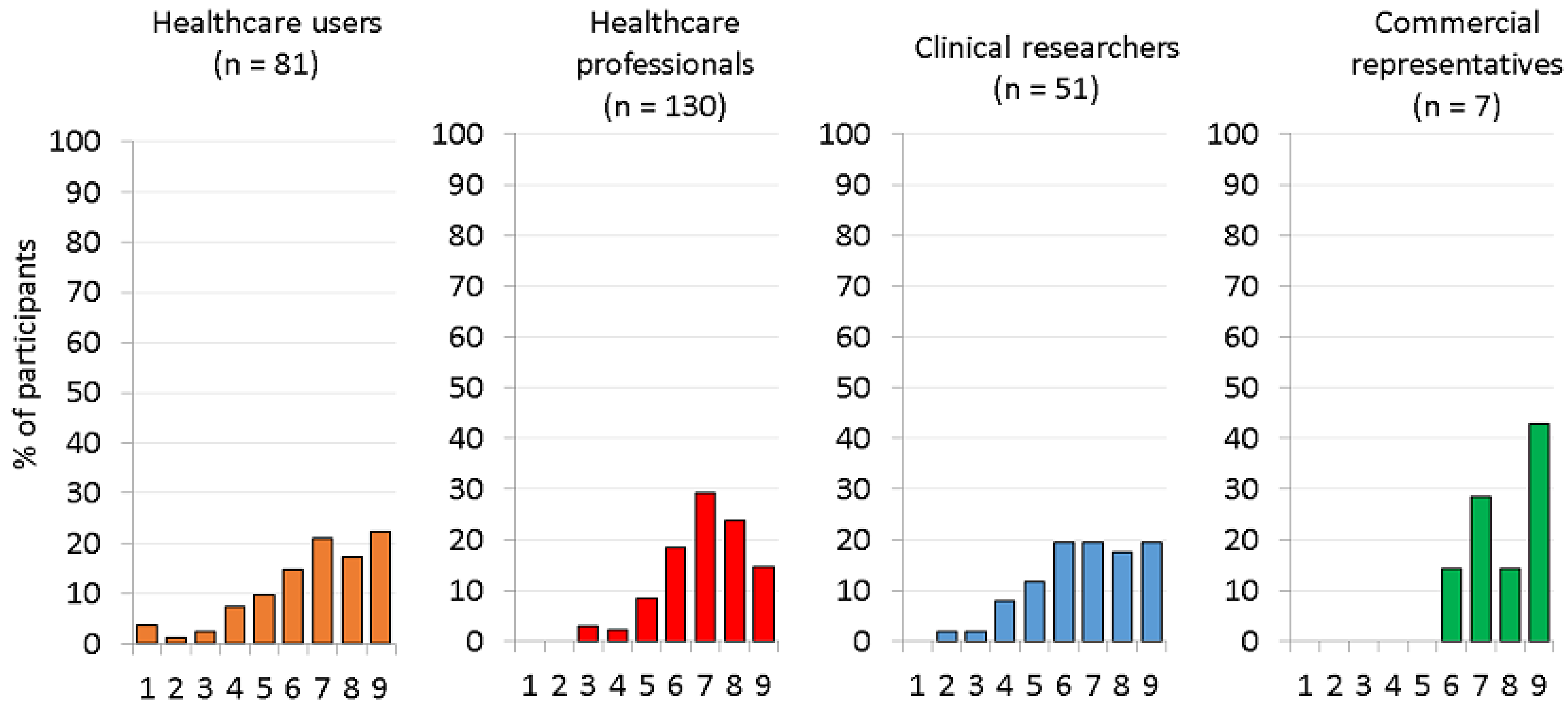

Domain Category:

Psychological effects

NIHR | National Institute for Health Research

Rating scale:

1	2	3	4	5	6	7	8	9
Not at all important			*Important but not critical*			*Critical*		

6. DISSATISFACTION WITH LIFE

CROSSSD

Being unhappy because you feel you should be achieving or should have achieved more in your life

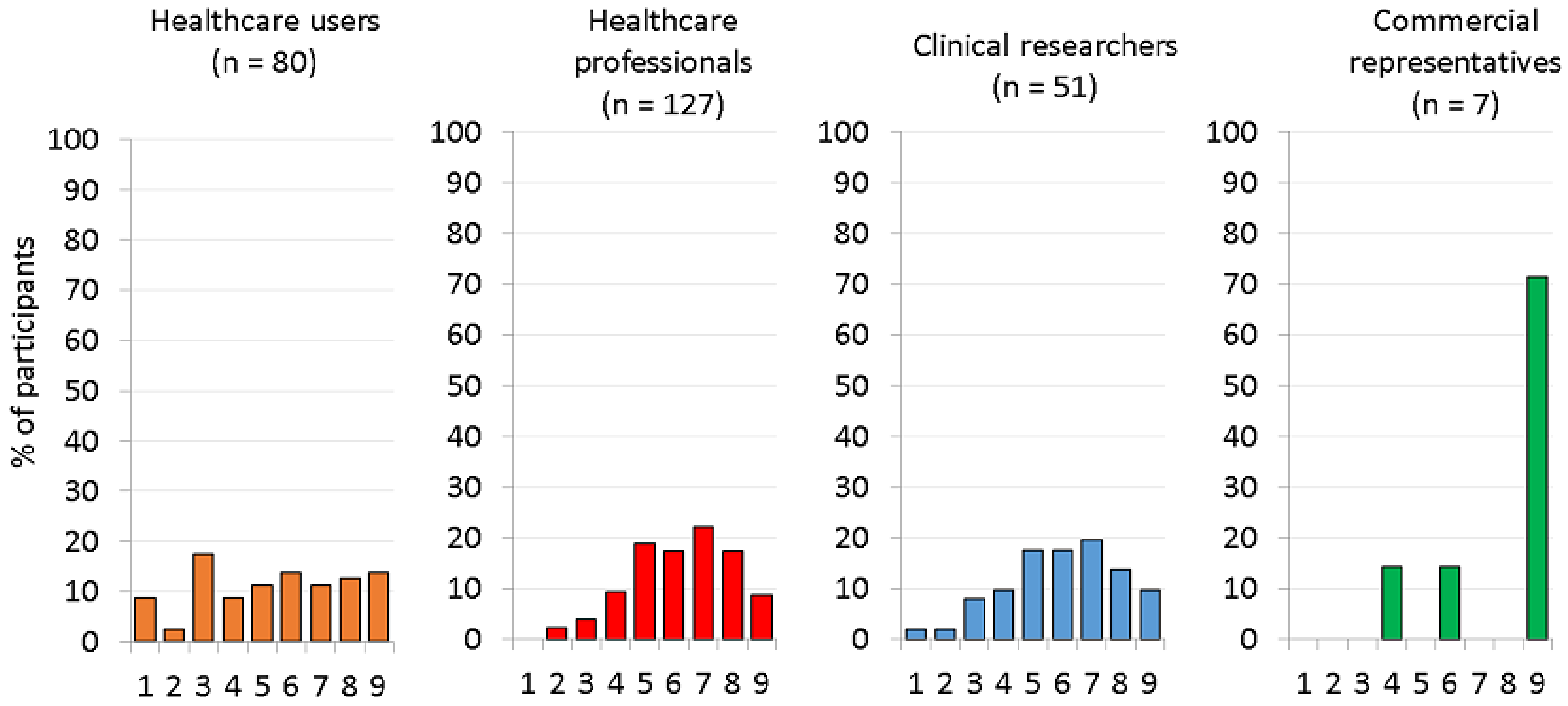

Domain Category:

Psychological effects

NIHR | National Institute for Health Research

Rating scale:

1	2	3	4	5	6	7	8	9
Not at all important			*Important but not critical*			*Critical*		

7. LISTENING EFFORT

Exerting greater effort to listen and follow a conversation.
This might consequently lead to feelings of tiredness and fatigue;
but those feelings would be a separate outcome domain

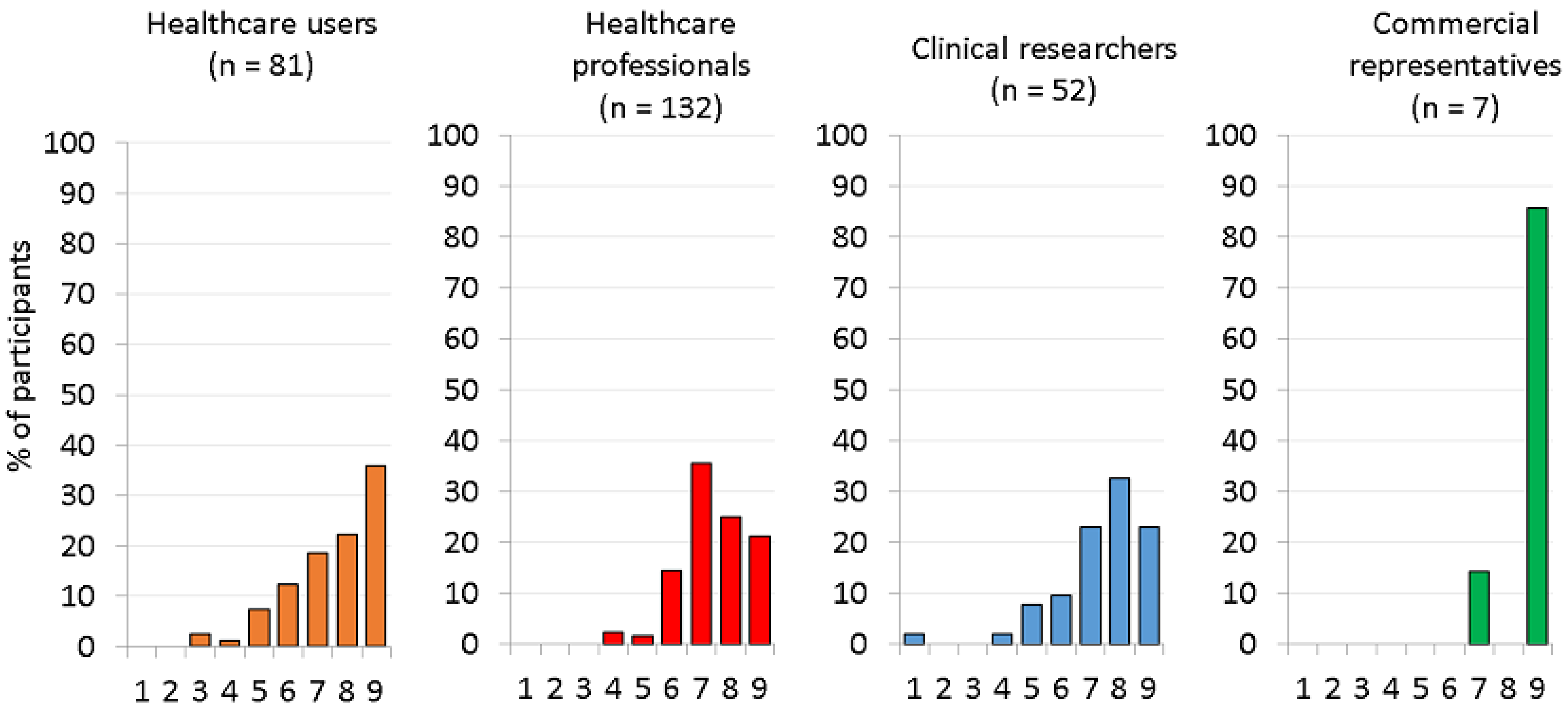

Domain Category:
Other effects

NIHR | National Institute for Health Research

Rating scale:

1	2	3	4	5	6	7	8	9
Not at all important			*Important but not critical*			*Critical*		

8. TREATMENT SATISFACTION

How the treatment meets your expectations or how pleased you are after receiving the treatment; or how likely you are to recommend the treatment

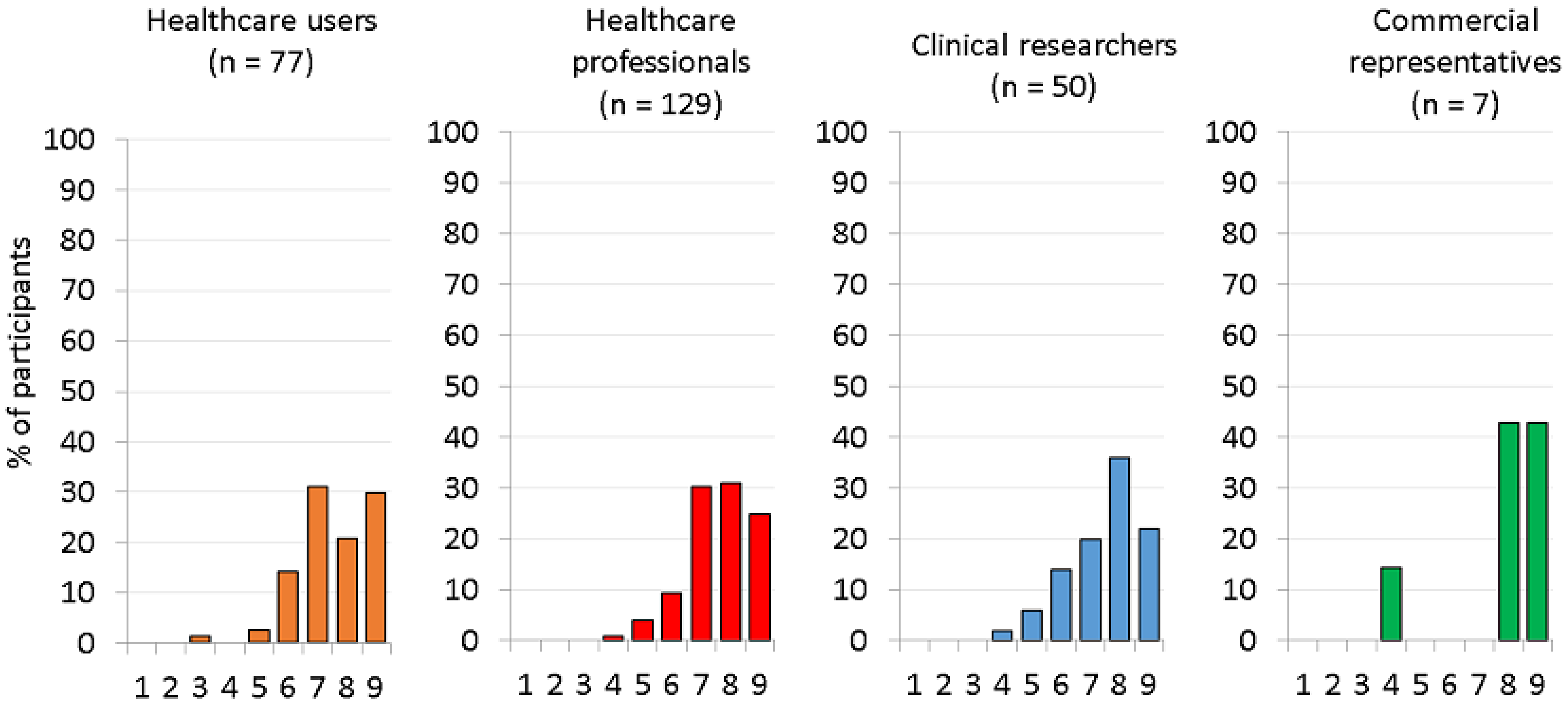

Domain Category: Factors related to the treatment being tested

Rating scale:

1	2	3	4	5	6	7	8	9
Not at all important			*Important but not critical*			*Critical*		

NIHR | National Institute for Health Research

9. DEVICE USAGE

How you use the device
(for example; in what situations; for how long)

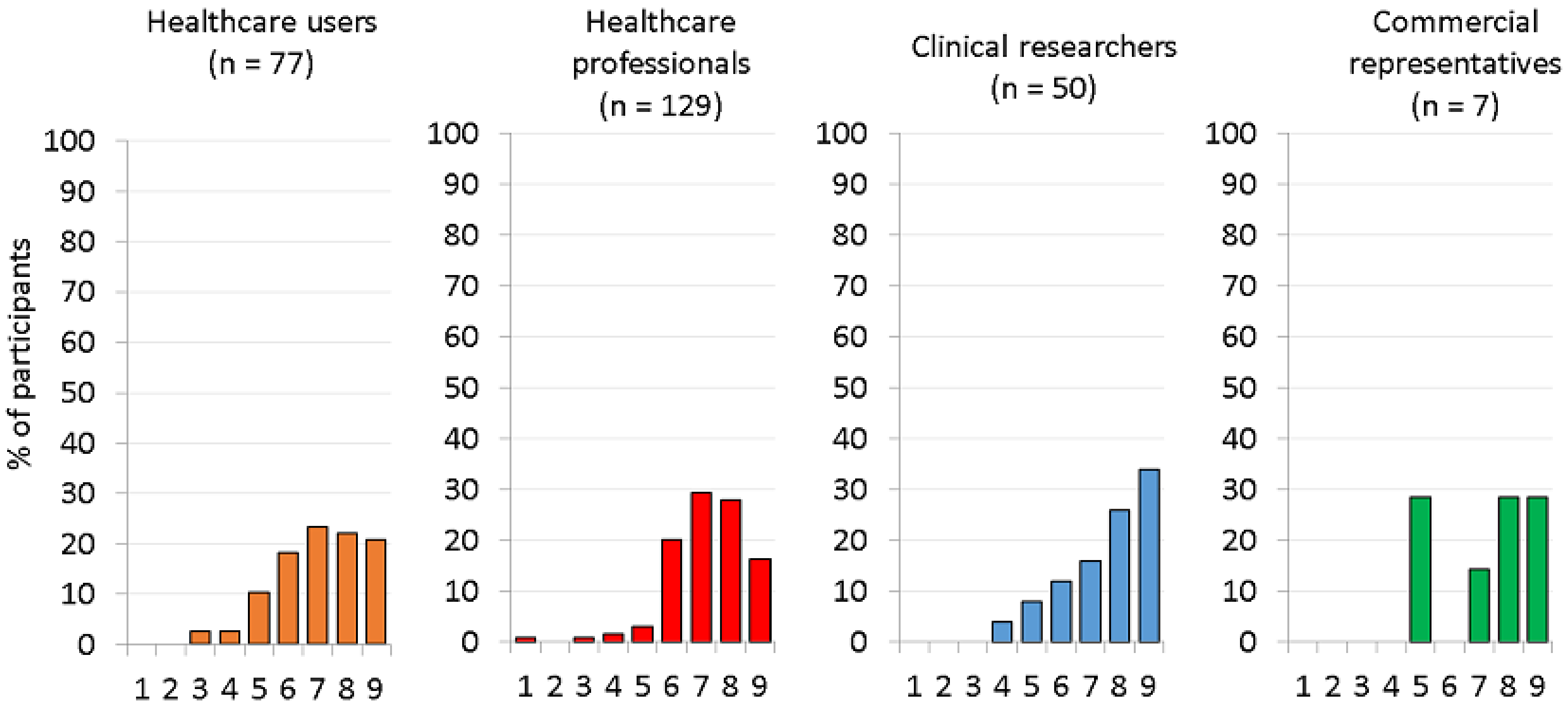

Domain Category: Factors related to the treatment being tested

Rating scale:

1	2	3	4	5	6	7	8	9
Not at all important			*Important but not critical*			*Critical*		

NIHR | National Institute for Health Research

10. DEVICE MALFUNCTION

The device does not work as it should or it stops working

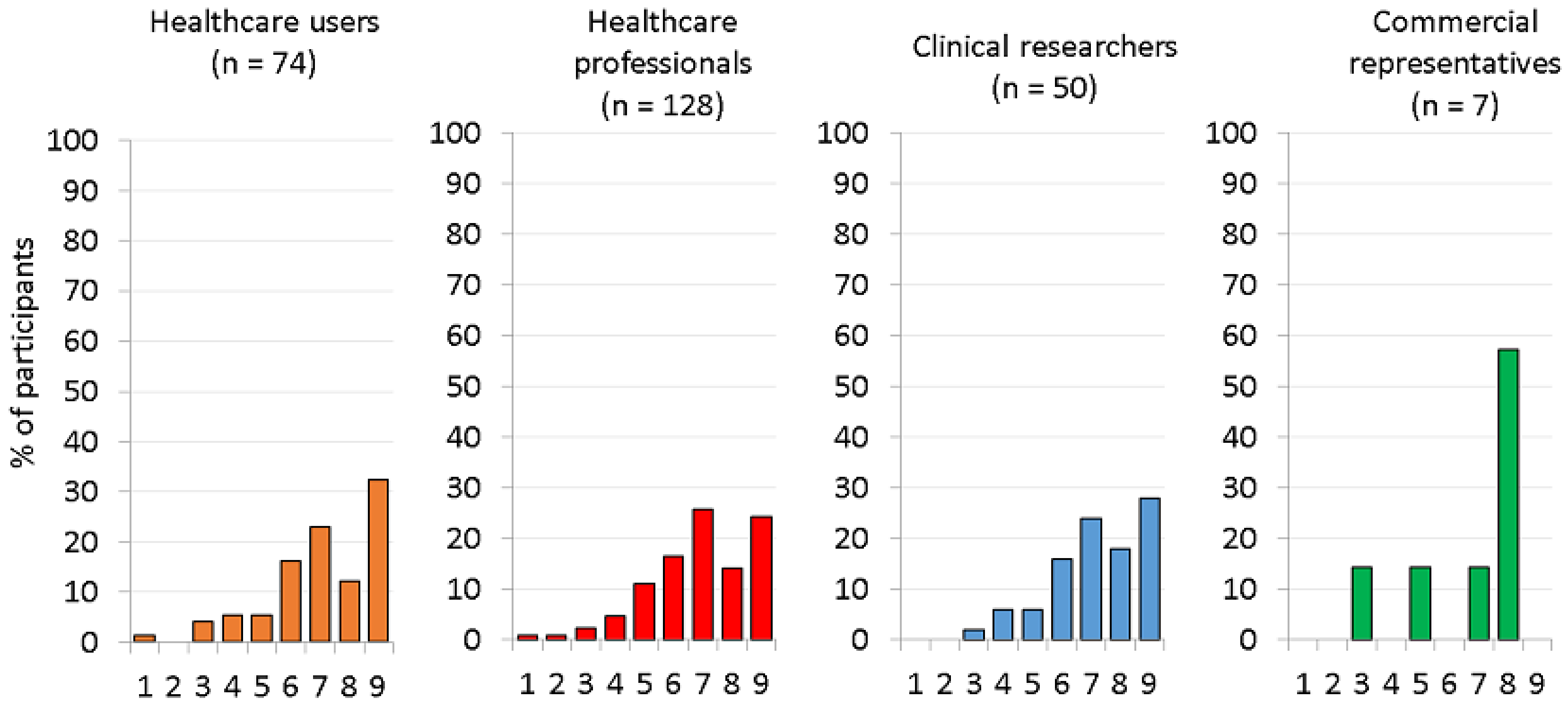

Domain Category: Factors related to the treatment being tested

Rating scale:

1	2	3	4	5	6	7	8	9
Not at all important			*Important but not critical*			*Critical*		

NIHR | National Institute for Health Research

11. ADVERSE EVENTS

Any bad or unexpected thing that happens during the time a treatment is being tested in a clinical trial

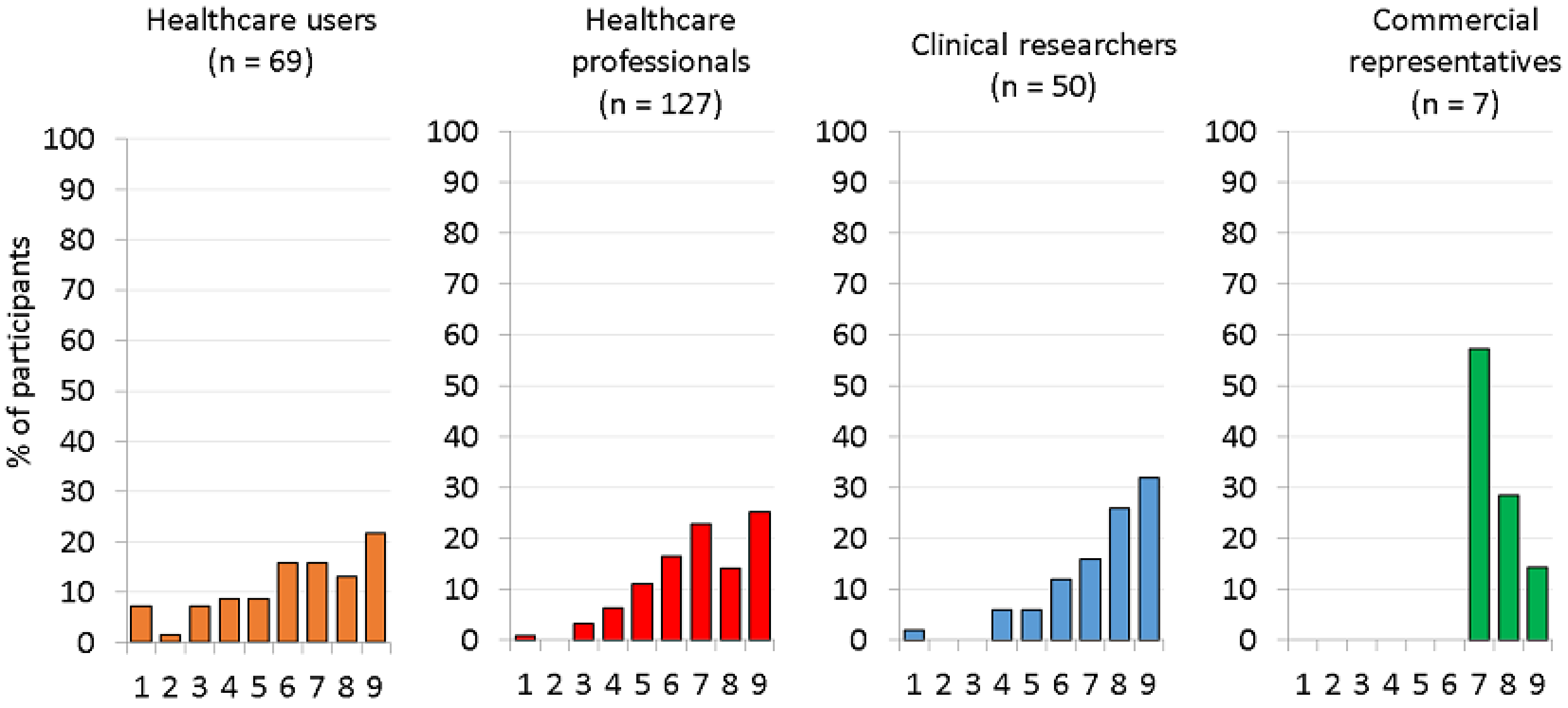

Domain Category: Factors related to the treatment being tested

NIHR | National Institute for Health Research

Rating scale:

1	2	3	4	5	6	7	8	9
Not at all important			*Important but not critical*			*Critical*		

12. AVOIDING SOCIAL SITUATIONS

Choosing not to go to particular social situations because of your hearing loss

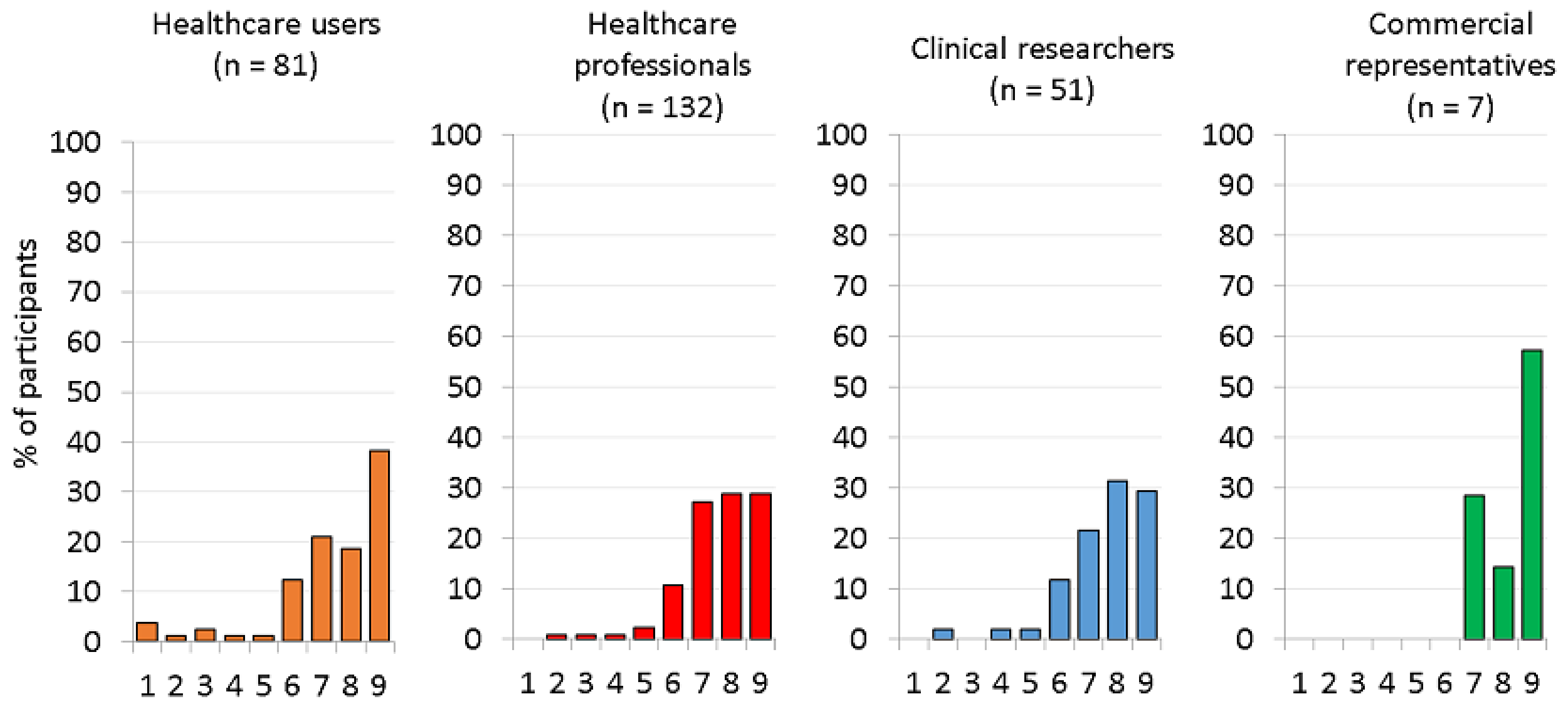

Domain Category: Health-related quality of life

Rating scale:

1	2	3	4	5	6	7	8	9
Not at all important			*Important but not critical*			*Critical*		

NIHR | National Institute for Health Research

13. IMPACT ON INDIVIDUAL ACTIVITIES

Effect of your hearing loss or your device on your choice to engage in individual activities (for example; travelling alone; swimming or watching TV / films / movies)

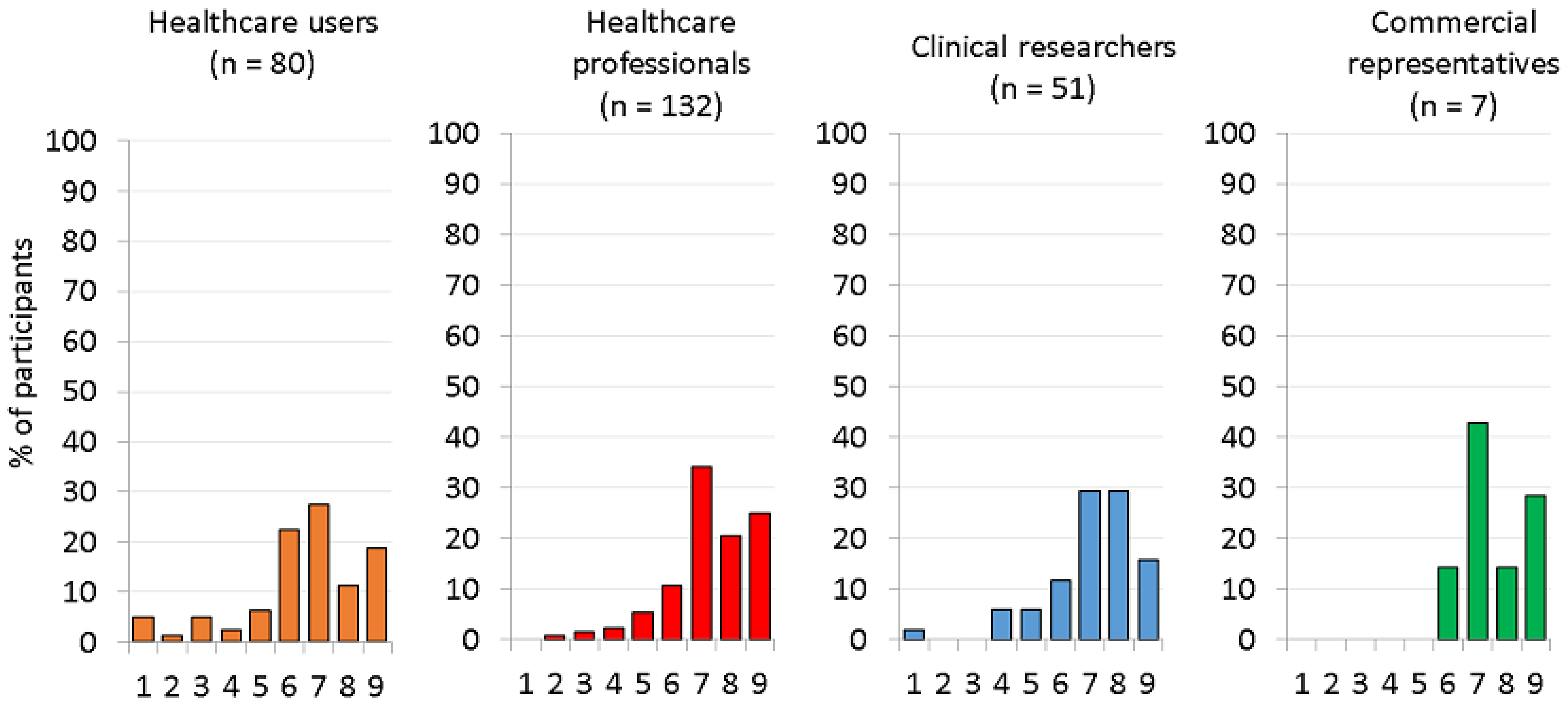

Domain Category: Health-related quality of life

Rating scale:

1	2	3	4	5	6	7	8	9
Not at all important			*Important but not critical*			*Critical*		

NIHR | National Institute for Health Research

14. IMPACT ON RELATIONSHIPS

Effect of your hearing loss or your device on making new relationships and maintaining relationships with a spouse or partner; family; friends and colleagues

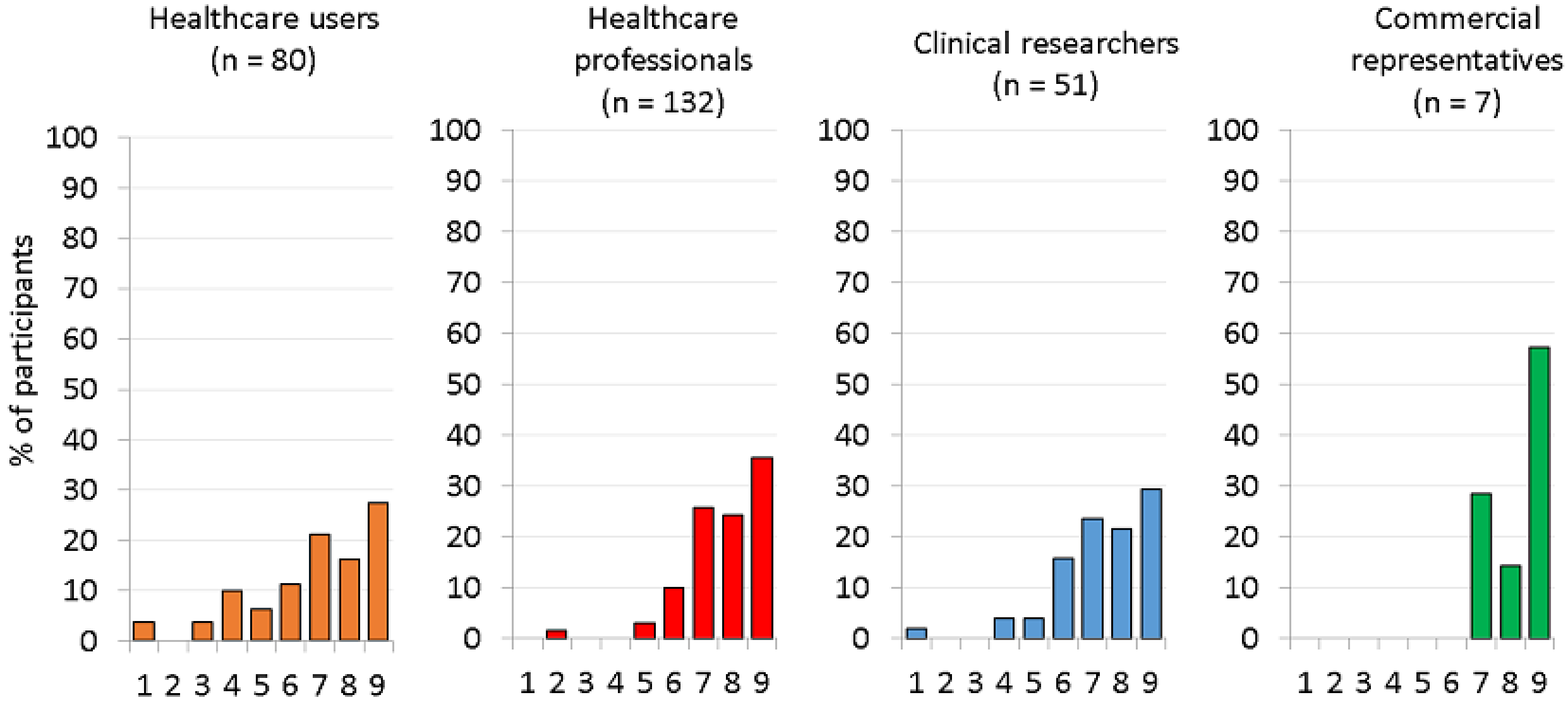

Domain Category: Health-related quality of life

Rating scale:

1	2	3	4	5	6	7	8	9
Not at all important			*Important but not critical*			*Critical*		

NIHR | National Institute for Health Research

15. IMPACT ON SOCIAL SITUATIONS

Your hearing loss or device limiting your ability to fully participate in the social world; especially in challenging situations or where a lot of effort is needed to follow the conversation (for example; at a restaurant; at the park; in a bar or at a party)

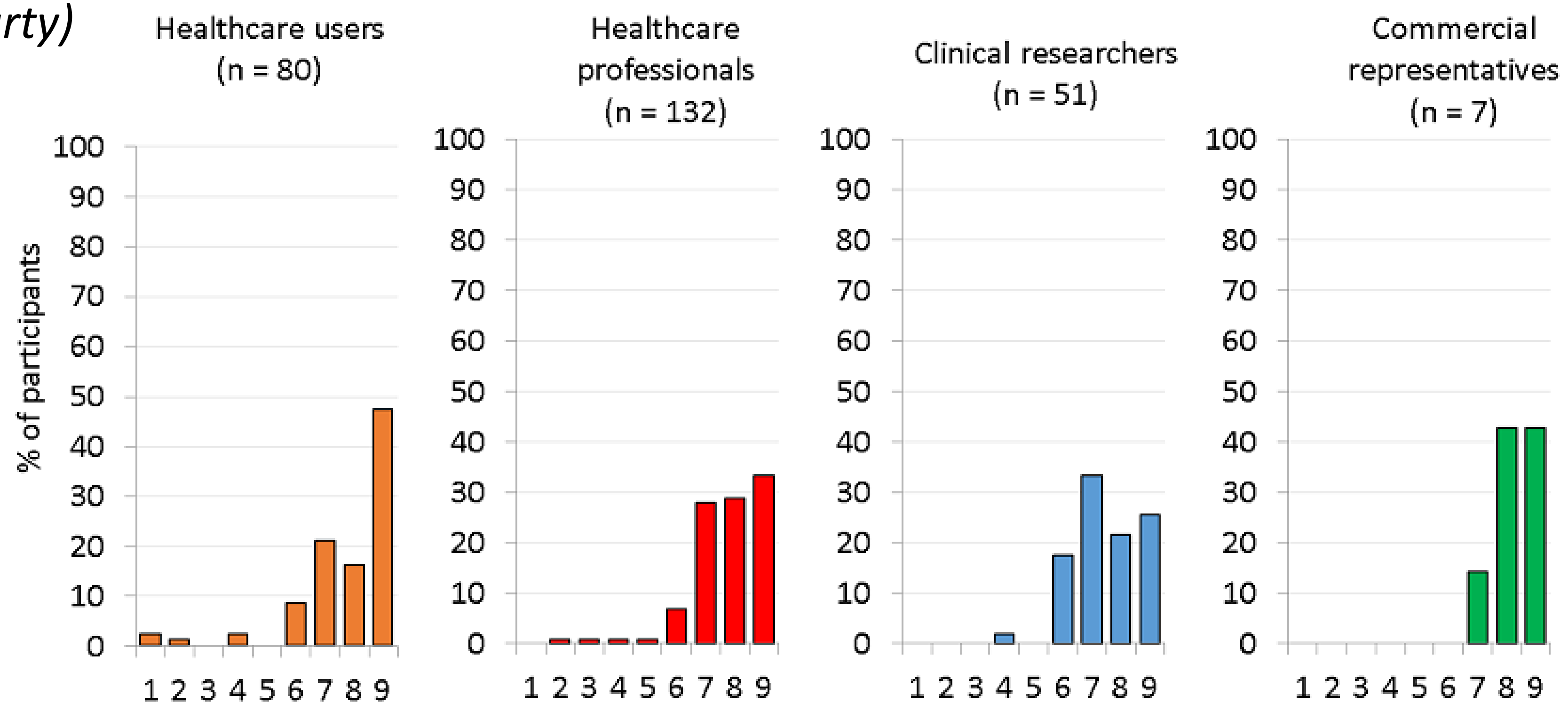

Domain Category: Health-related quality of life

Rating scale:

1	2	3	4	5	6	7	8	9
Not at all important			*Important but not critical*			*Critical*		

NIHR | National Institute for Health Research

16. IMPACT ON WORK

Effect of your hearing loss or device on your ability to carry out work tasks or job roles; or advancing your career

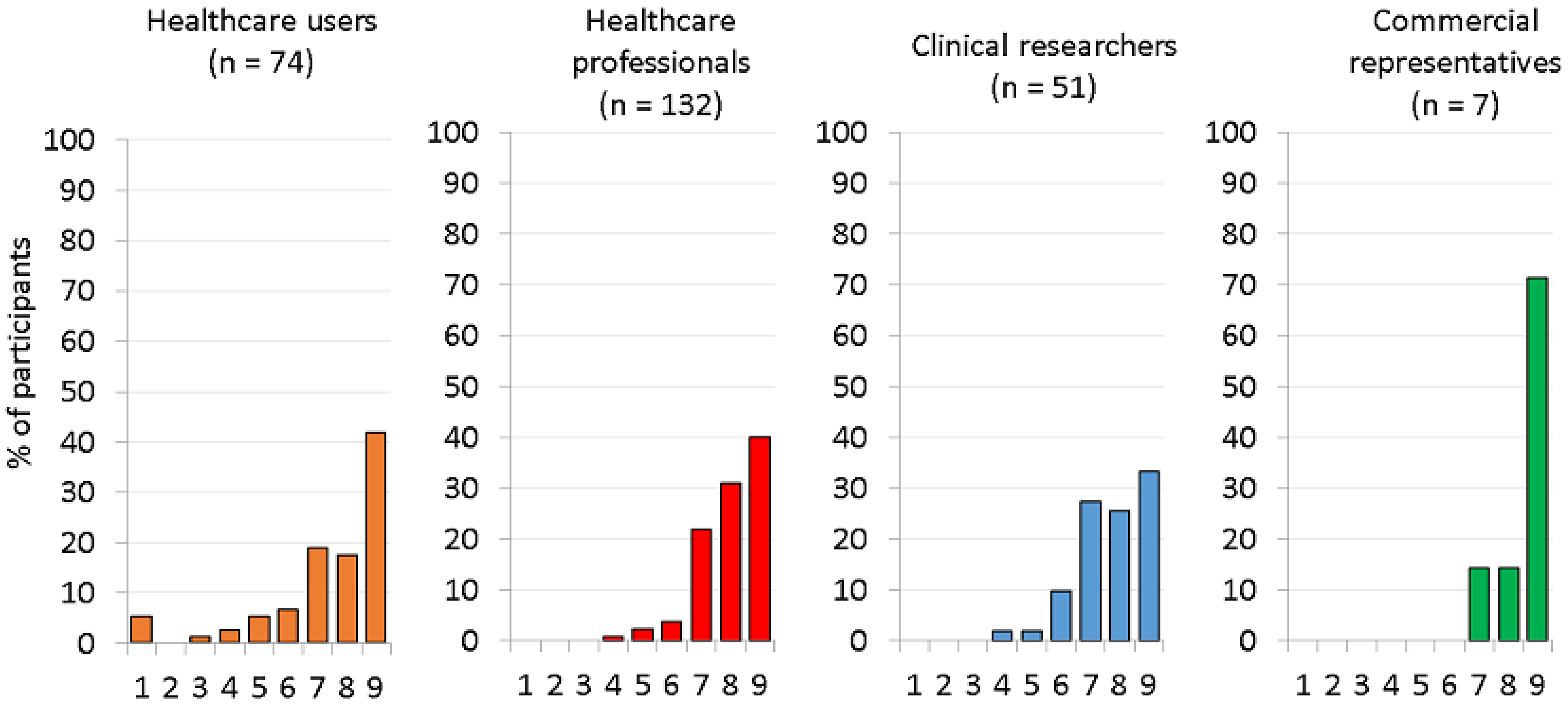

Domain Category: Health-related quality of life

Rating scale:

1	2	3	4	5	6	7	8	9
Not at all important			*Important but not critical*			*Critical*		

NIHR | National Institute for Health Research

17. BEING AWARE OF A SOUND

Being aware of a sound and recognising what that sound is (for example; being aware that someone has started to speak)

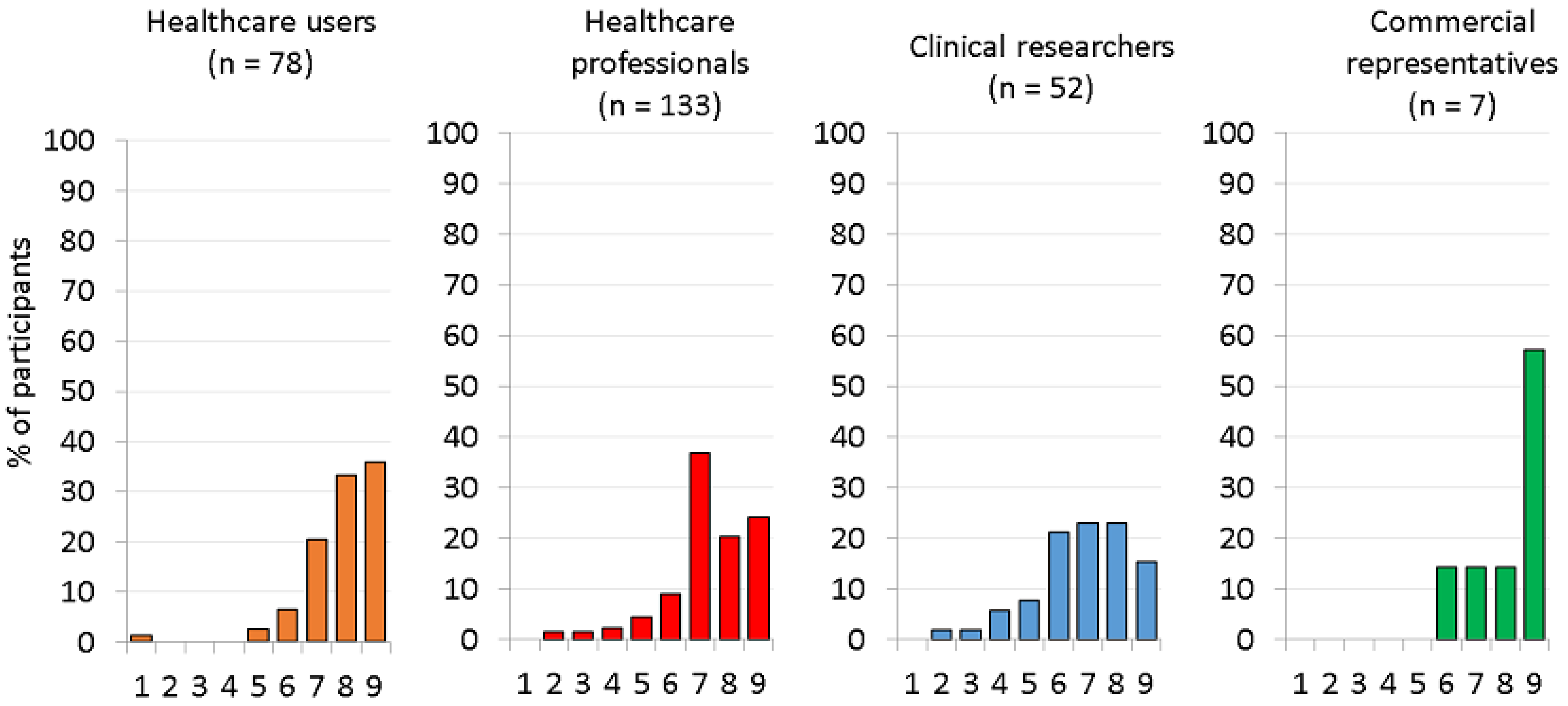

Domain Category: Hearing disability

National Institute for Health Research

Rating scale:

1	2	3	4	5	6	7	8	9
Not at all important			*Important but not critical*			*Critical*		

18. LISTENING IN COMPLEX SITUATIONS

The difficulty experienced when listening to a sound while separating it out from a background of other sounds

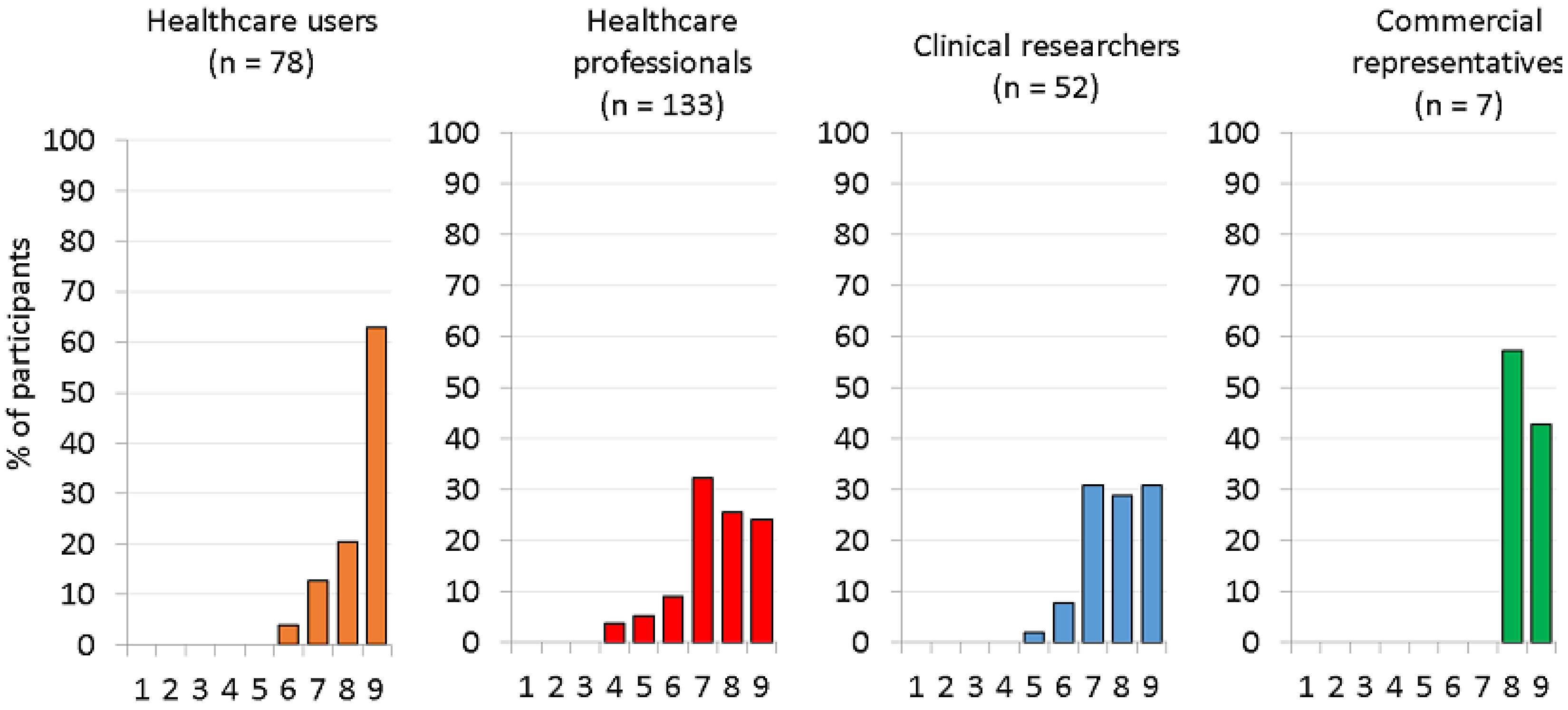

Domain Category:
Hearing disability

NIHR | National Institute for Health Research

Rating scale:

1	2	3	4	5	6	7	8	9
Not at all important			*Important but not critical*			*Critical*		

19. LISTENING IN REVERBERANT CONDITIONS

CROSSSD

The difficulty experienced when listening in places where the sound reflects off the walls; floor or ceiling (echoes); creating a blurred sound. For example; understanding announcements in train stations or airports

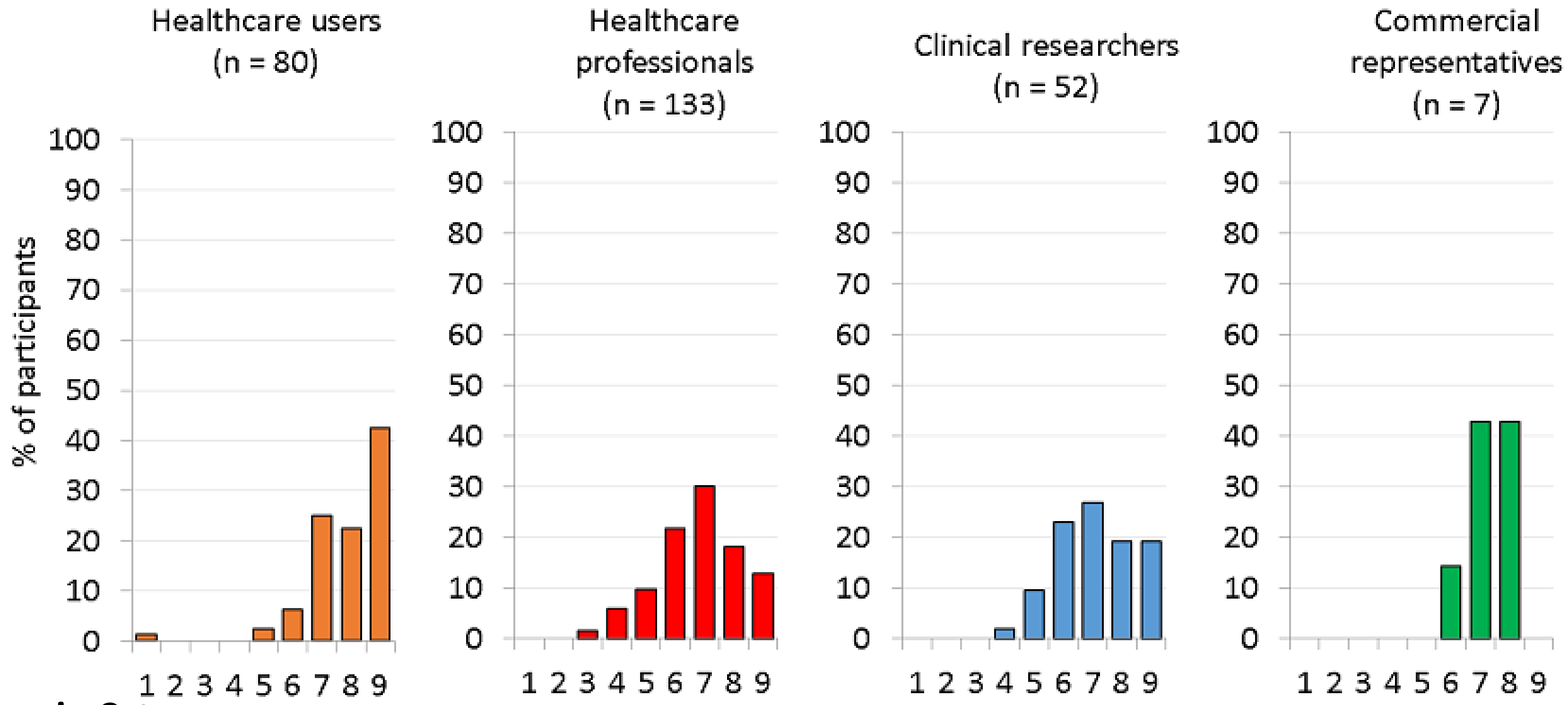

Domain Category:
Hearing disability

Rating scale:

1	2	3	4	5	6	7	8	9
Not at all important			*Important but not critical*			*Critical*		

NIHR | National Institute for Health Research

20. ONE-TO-ONE CONVERSATION IN QUIET

Listening and understanding one person; in a quiet environment

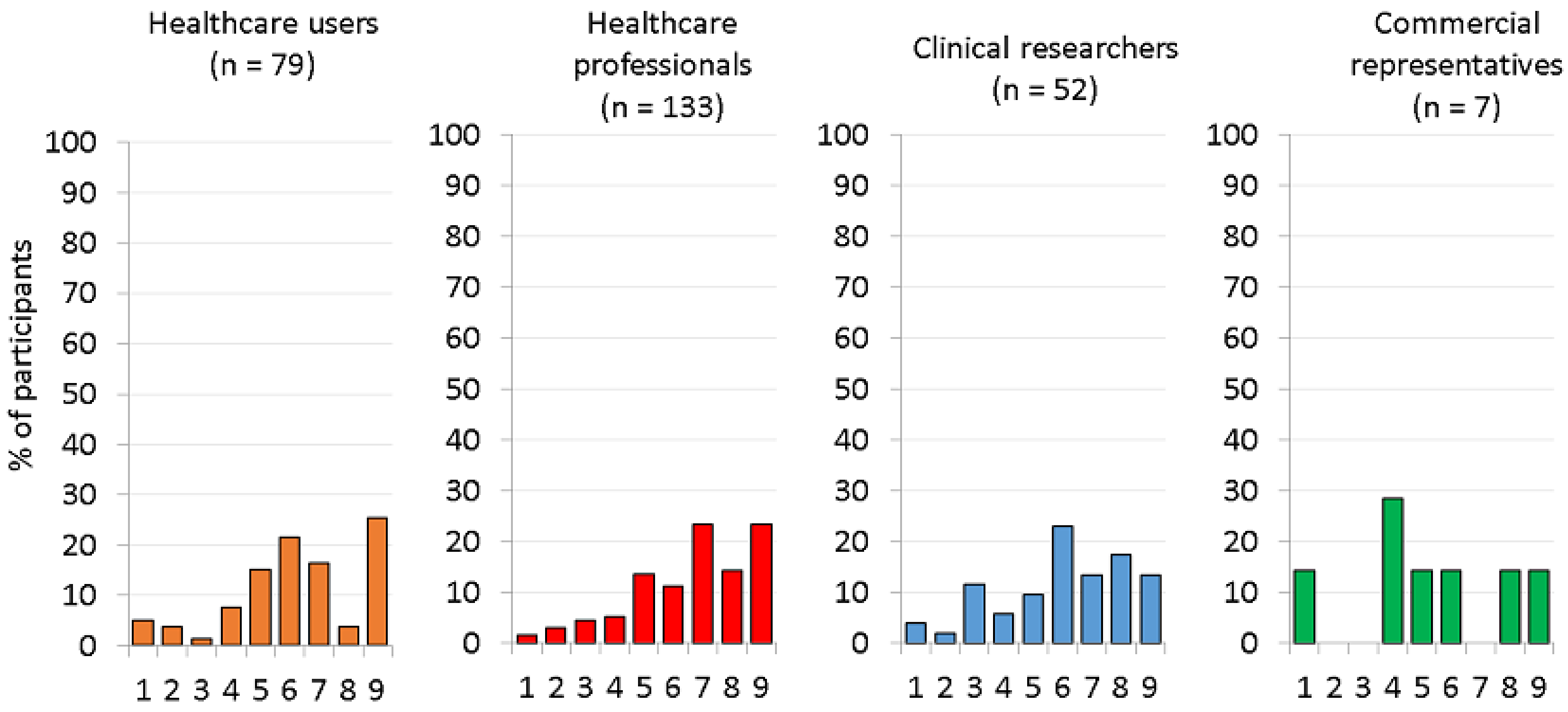

Domain Category:
Hearing disability

NIHR | National Institute for Health Research

Rating scale:

1	2	3	4	5	6	7	8	9
Not at all important			*Important but not critical*			*Critical*		

21. GROUP CONVERSATION IN QUIET

Listening and following a conversation between a group of people; in a quiet environment

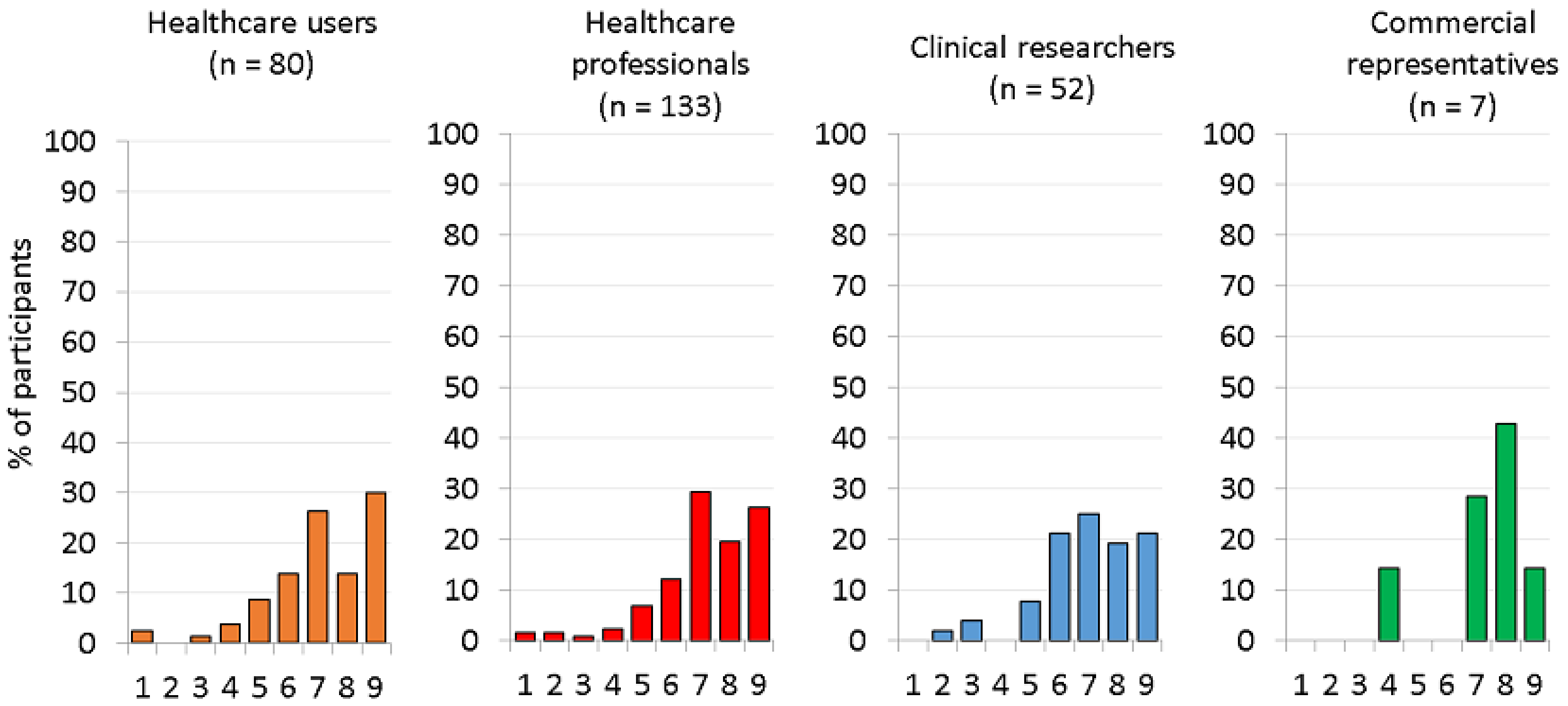

Domain Category:
Hearing disability

Rating scale:

1	2	3	4	5	6	7	8	9
Not at all important			*Important but not critical*			*Critical*		

NIHR | National Institute for Health Research

22. ONE-TO-ONE CONVERSATION IN GENERAL NOISE

Listening and understanding one person; in a noisy environment

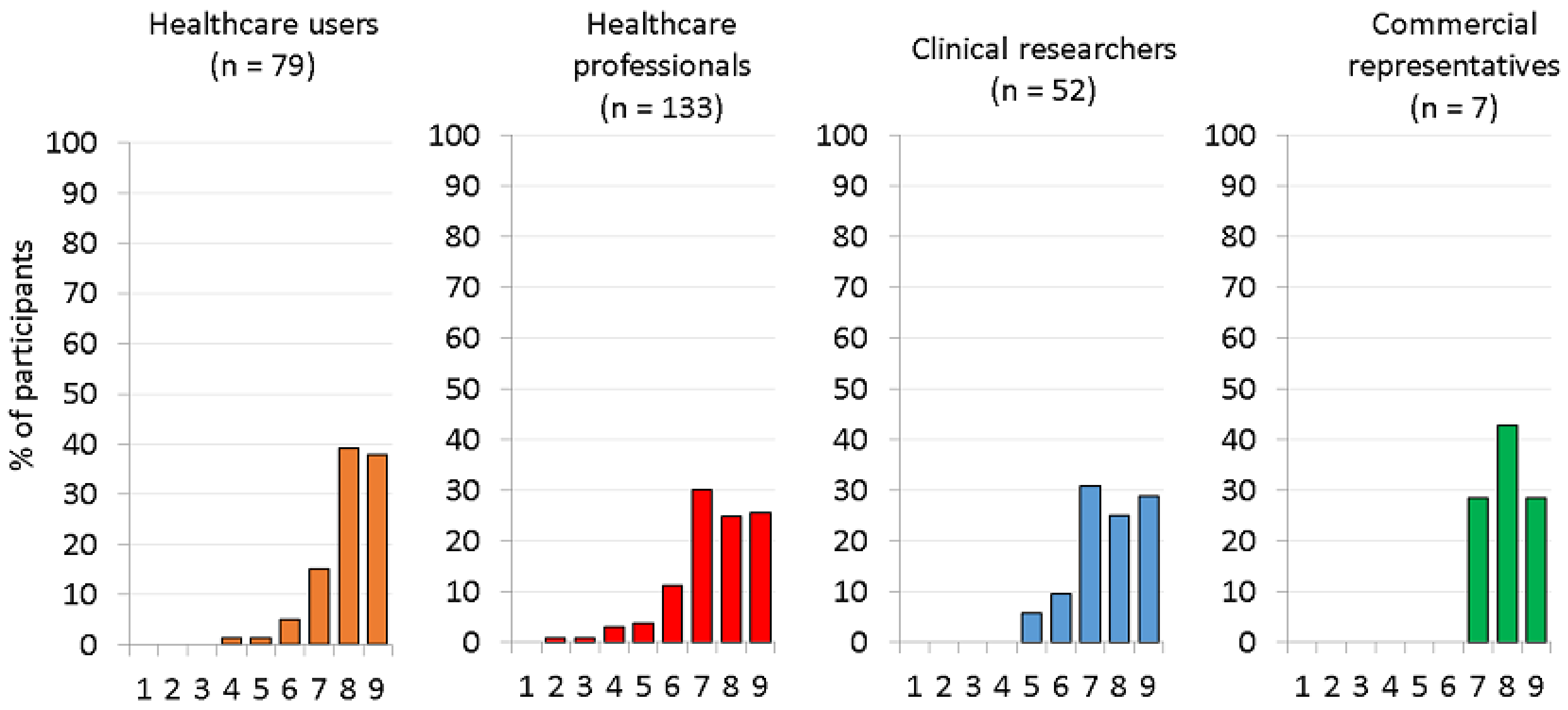

Domain Category:
Hearing disability

NIHR National Institute for Health Research

Rating scale:

1	2	3	4	5	6	7	8	9
Not at all important			*Important but not critical*			*Critical*		

23. GROUP CONVERSATION IN NOISY SOCIAL SITUATIONS

Listening and following a conversation between a group of people; when others are talking in the background

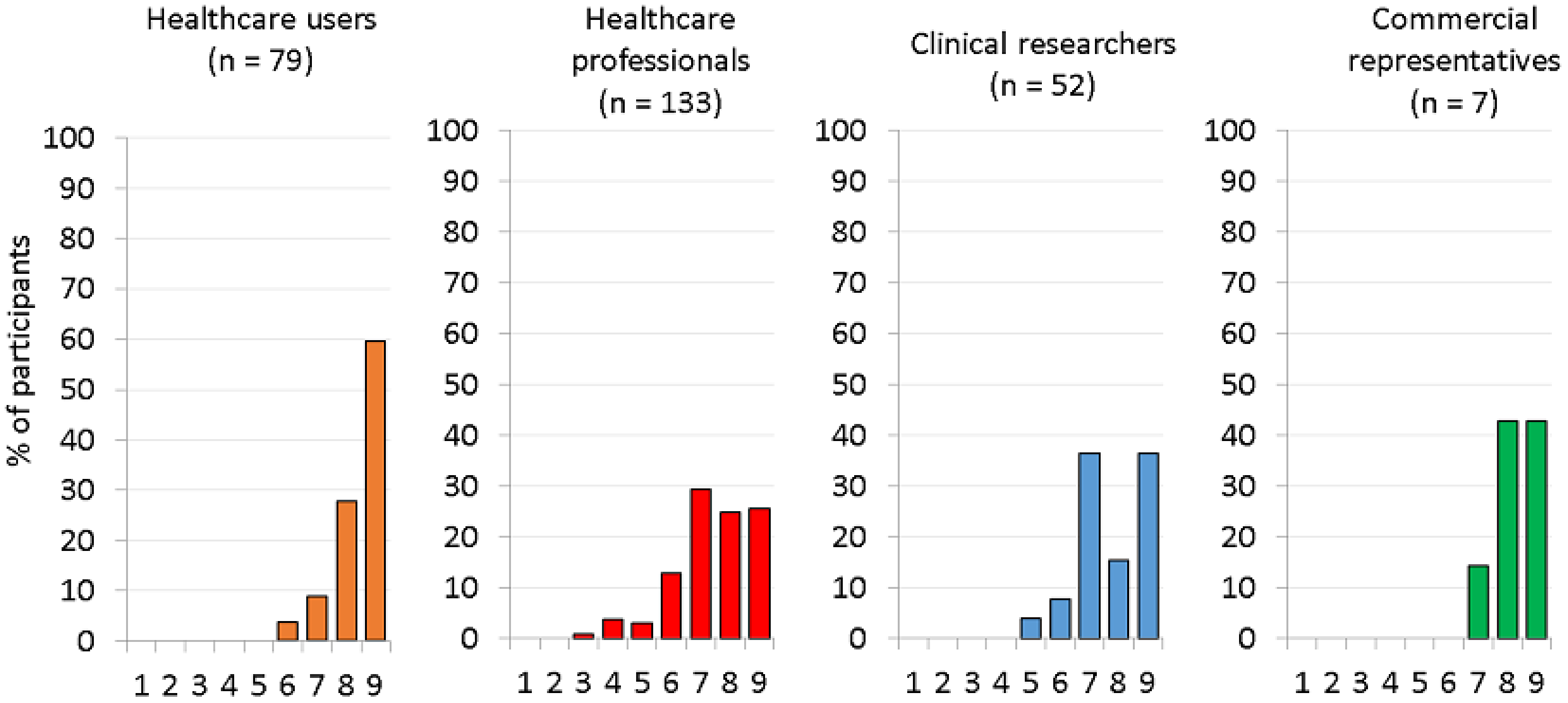

Domain Category:
Hearing disability

NIHR | National Institute for Health Research

Rating scale:

1	2	3	4	5	6	7	8	9
Not at all important			*Important but not critical*			*Critical*		

24. SOUND LOCALISATION

Knowing where a sound is coming from

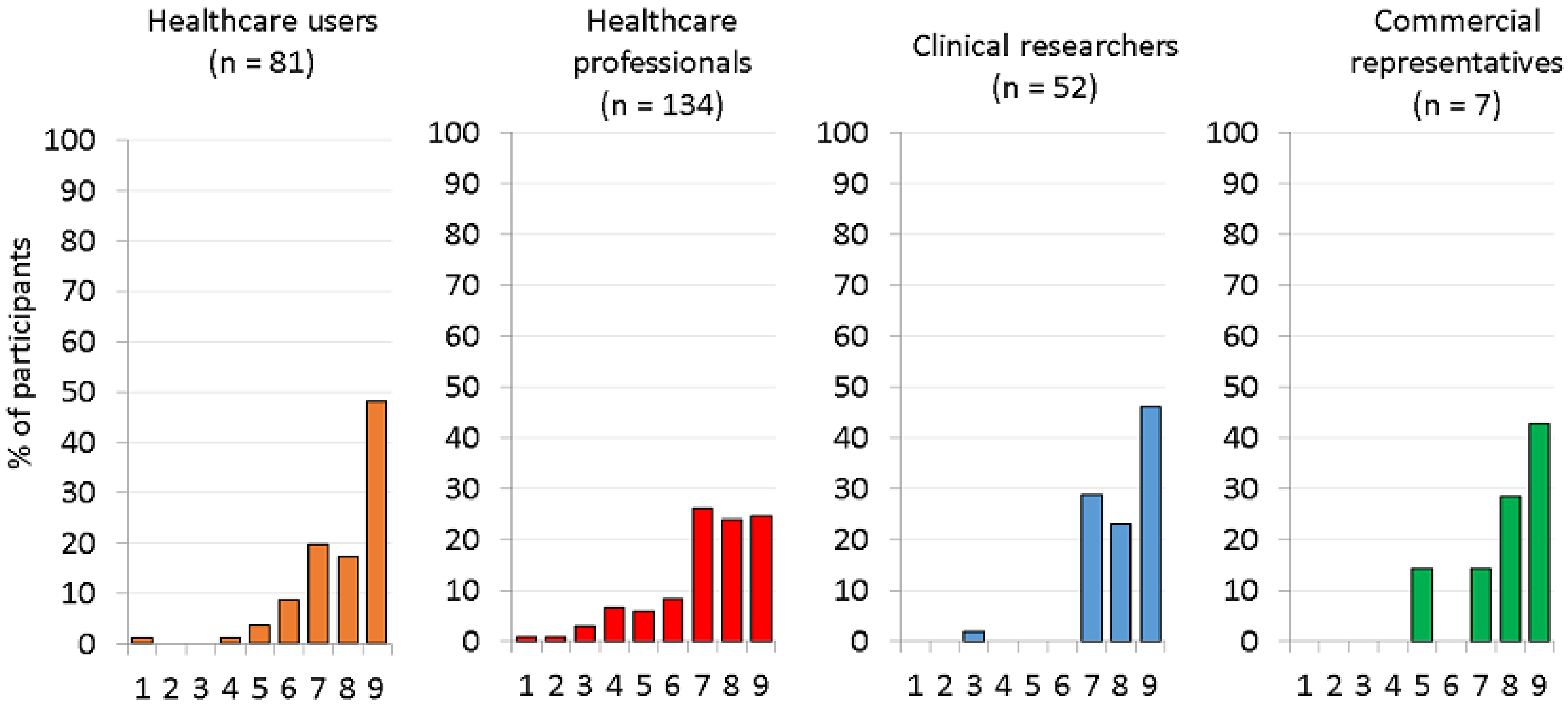

Domain Category:

Spatial hearing

NIHR | National Institute for Health Research

Rating scale:

1	2	3	4	5	6	7	8	9
Not at all important			*Important but not critical*			*Critical*		

25. SOUND DISTANCE

Knowing if a sound is close by or far away

CROSSSD

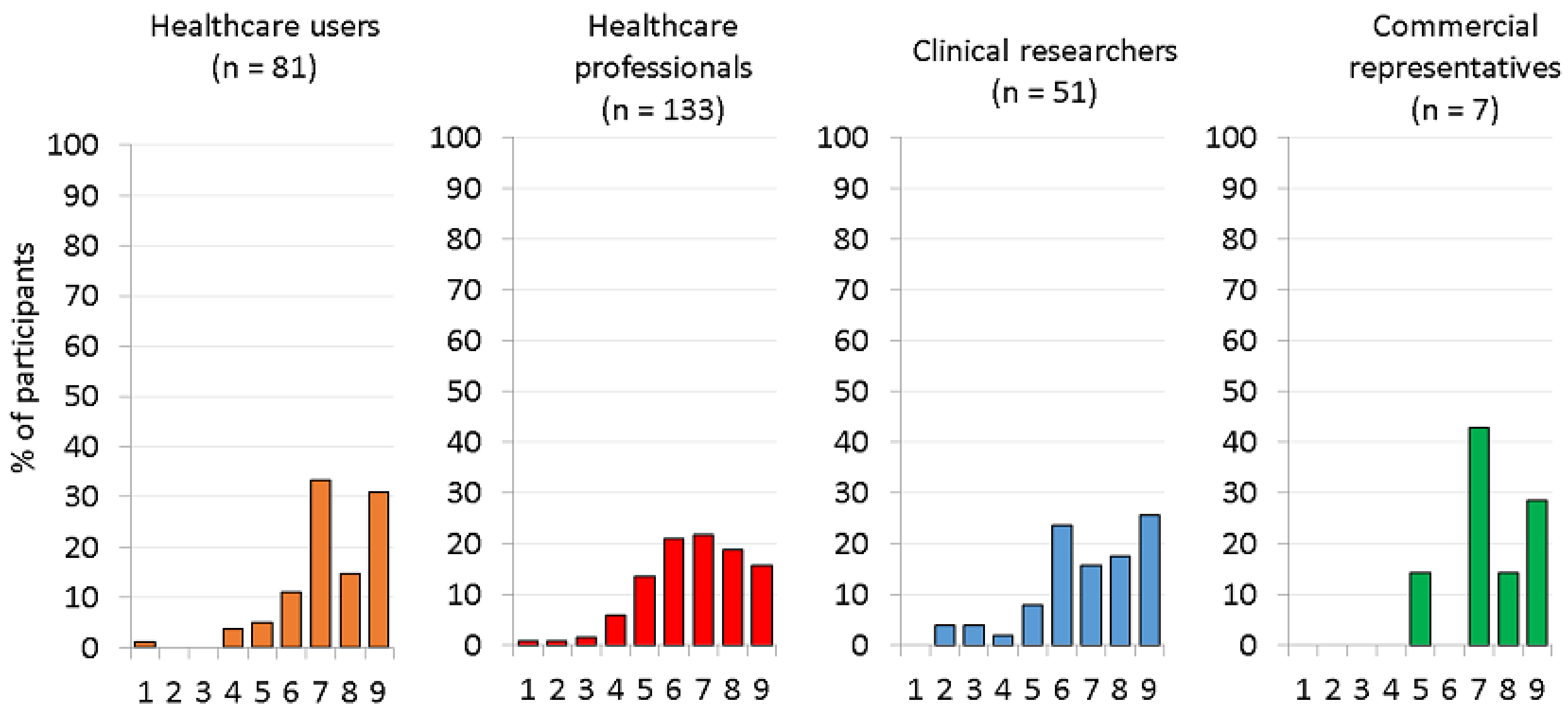

Domain Category:

Spatial hearing

NIHR | National Institute for Health Research

Rating scale:

1	2	3	4	5	6	7	8	9
Not at all important			*Important but not critical*			*Critical*		

26. SPATIAL ORIENTATION

Knowing where you are in relation to the position of a sound source

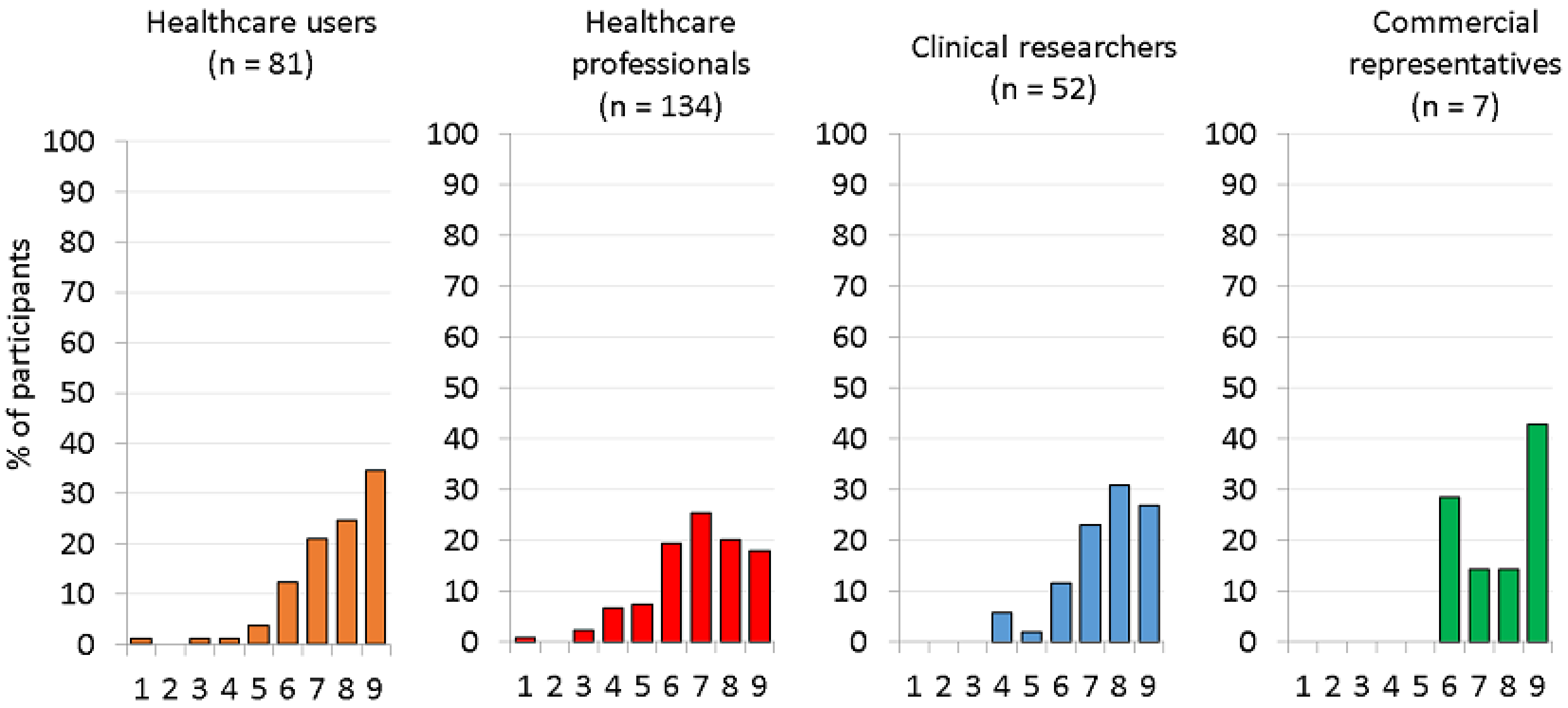

Domain Category:

Spatial hearing

NIHR | National Institute for Health Research

Rating scale:

1	2	3	4	5	6	7	8	9
Not at all important			*Important but not critical*			*Critical*		

27. ENJOYMENT OF LISTENING TO MUSIC

Appreciating 'stereo'; '3-dimensional' or 'surround sound' quality of live or recorded music

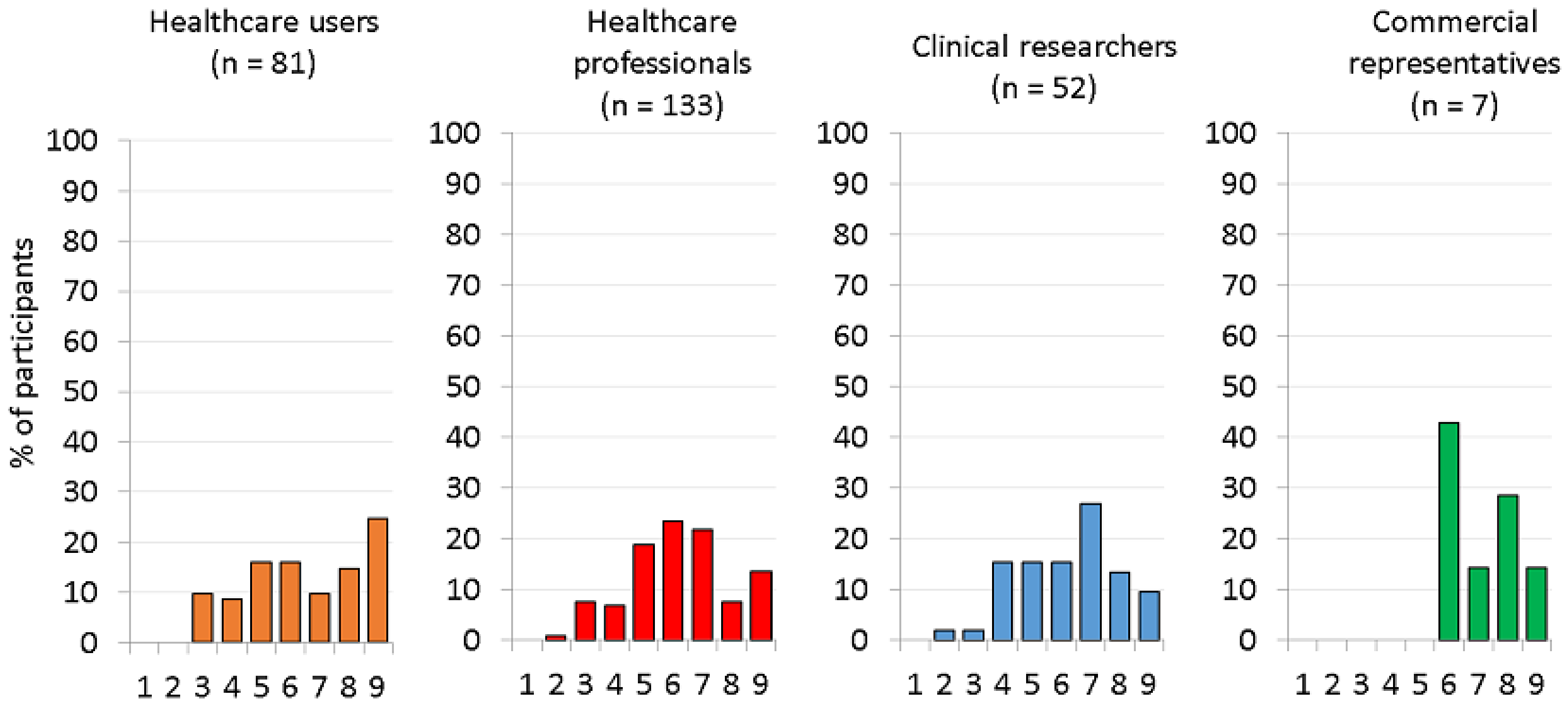

Domain Category:
Spatial hearing

NIHR | National Institute for Health Research

Rating scale:

1	2	3	4	5	6	7	8	9
Not at all important			*Important but not critical*			*Critical*		

28. PHYSICAL TIREDNESS

Tiredness or fatigue from the effort of listening or when you need to turn your head repeatedly to listen in social situations

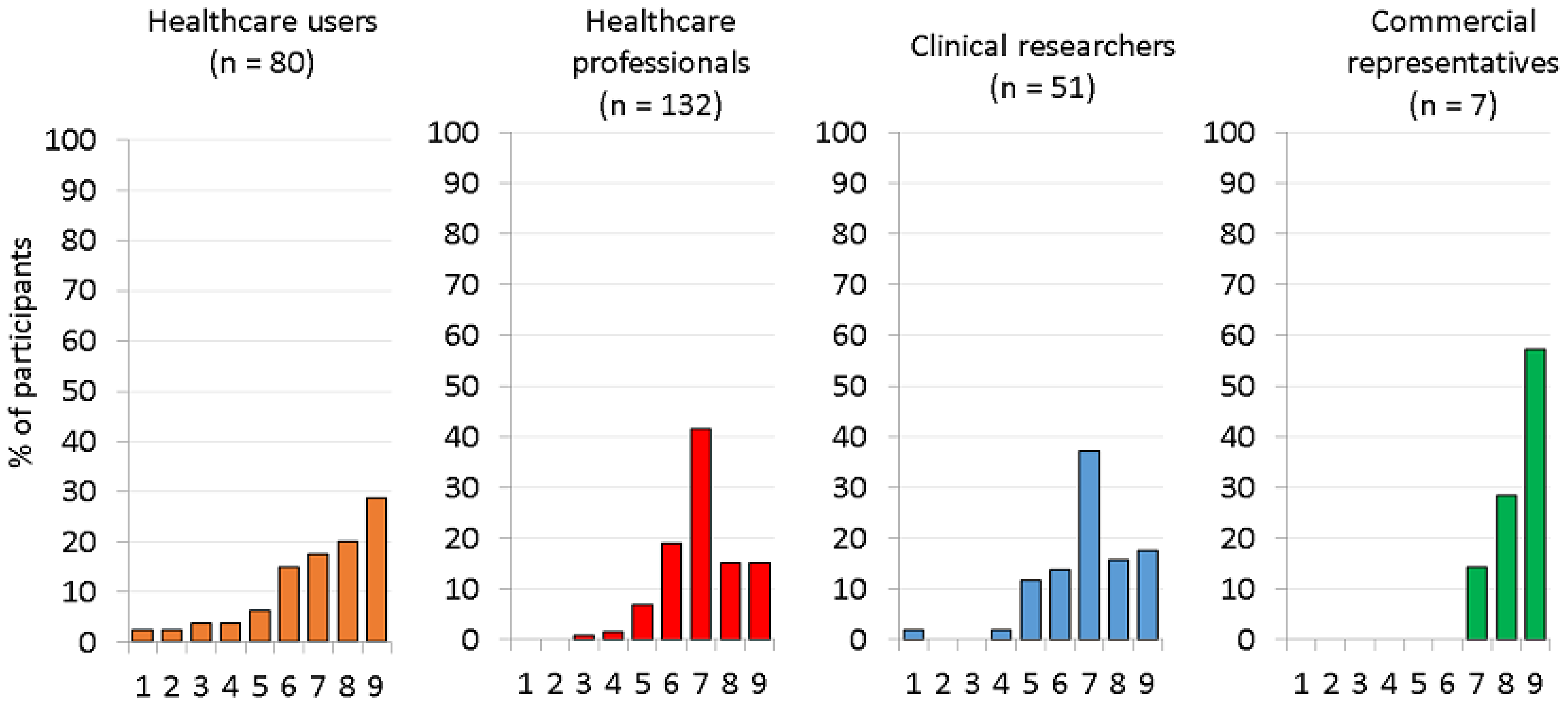

Domain Category:

Physical effects

NIHR | National Institute for Health Research

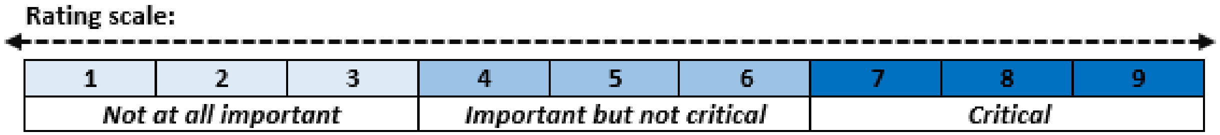

29. BALANCE PROBLEMS

Feeling unbalanced and the effect it has on your ability to walk or move normally

CROSSSD
CORE REHABILITATION OUTCOME SET for SINGLE SIDED DEAFNESS

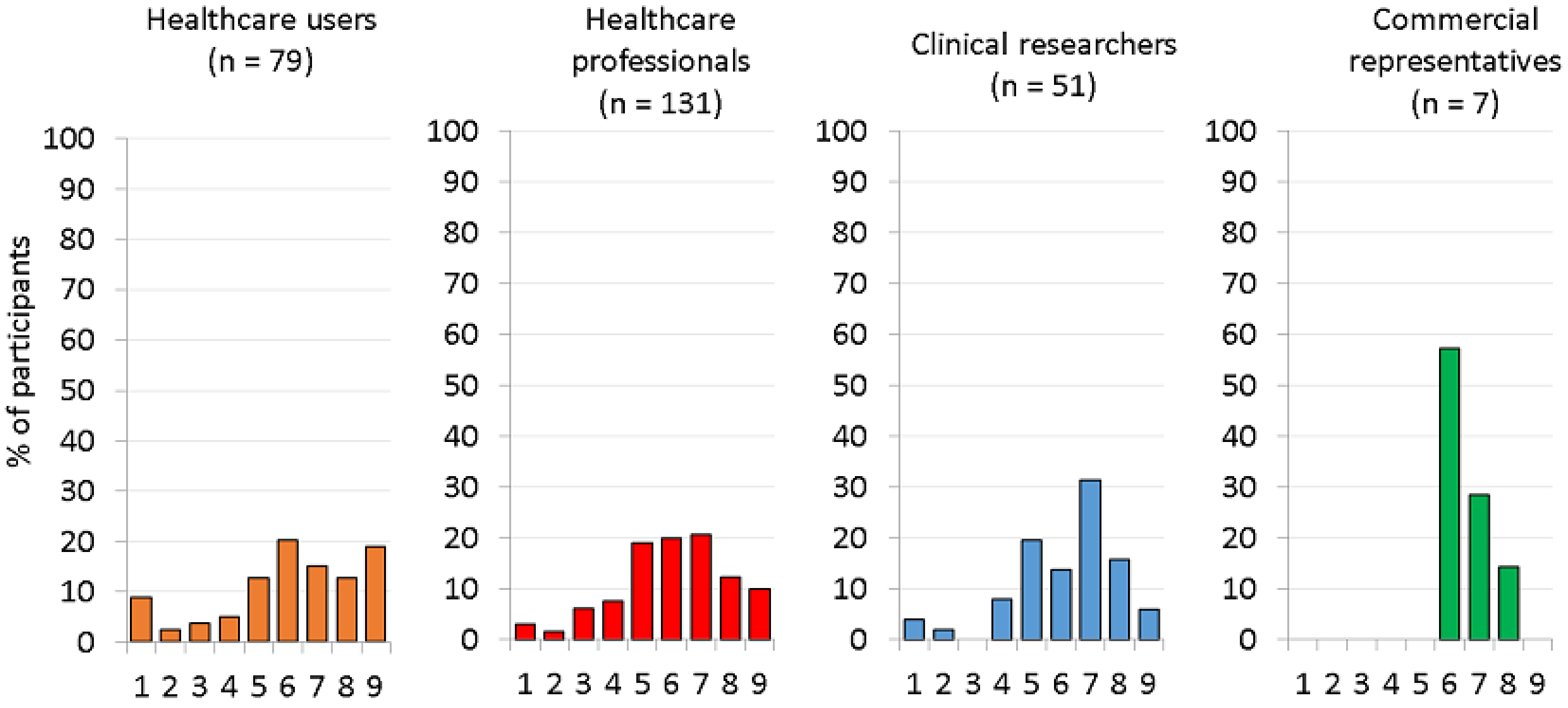

Domain Category:
Physical effects

NIHR | National Institute for Health Research

Rating scale:

1	2	3	4	5	6	7	8	9
Not at all important			*Important but not critical*			*Critical*		

30. MANUAL DEXTERITY

Having the fine motor skills needed to use your device effectively (for example; putting the device on; changing the batteries)

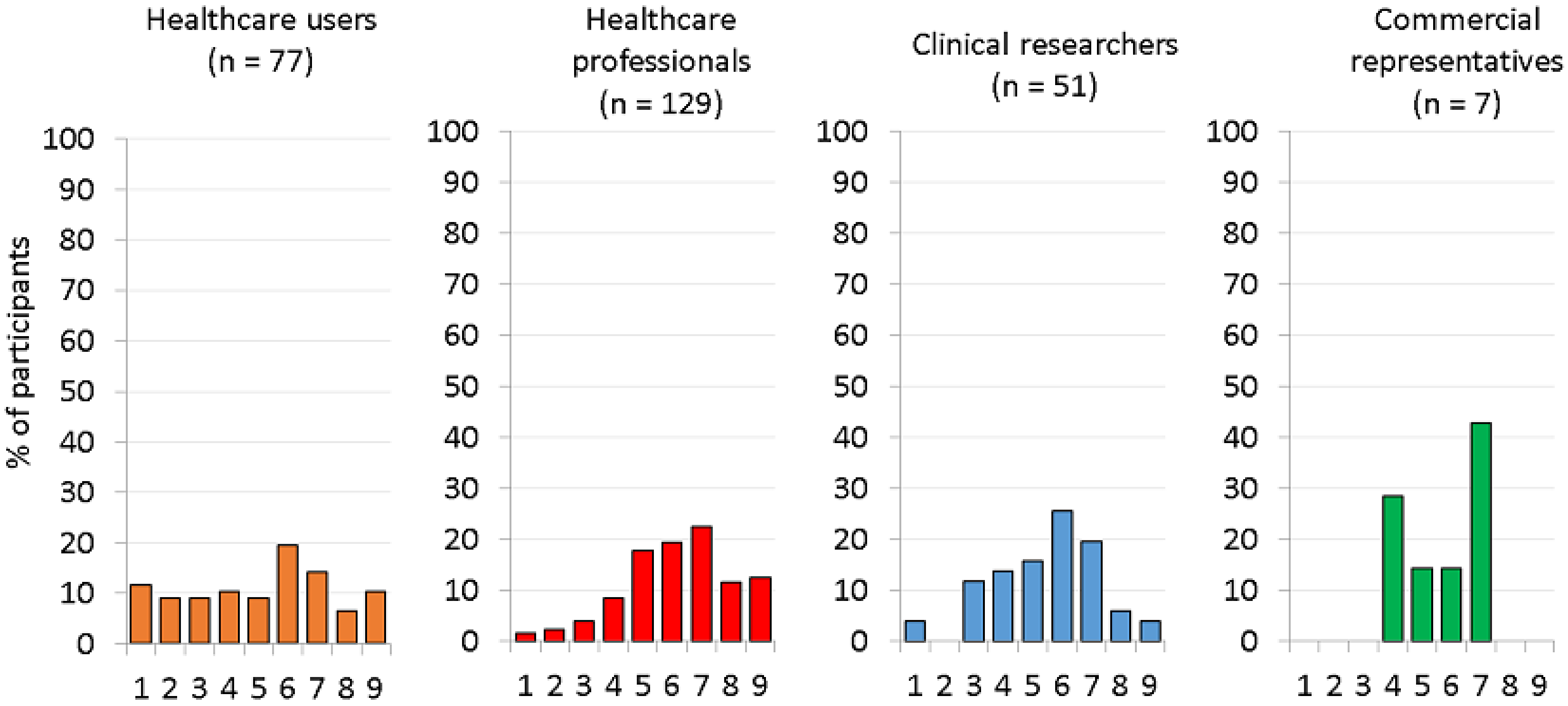

Domain Category:

Physical effects

NIHR | National Institute for Health Research

Rating scale:

1	2	3	4	5	6	7	8	9
Not at all important			*Important but not critical*			*Critical*		

31. TINNITUS-RELATED BRAIN CHANGES

Changes in brain structure or function associated with tinnitus

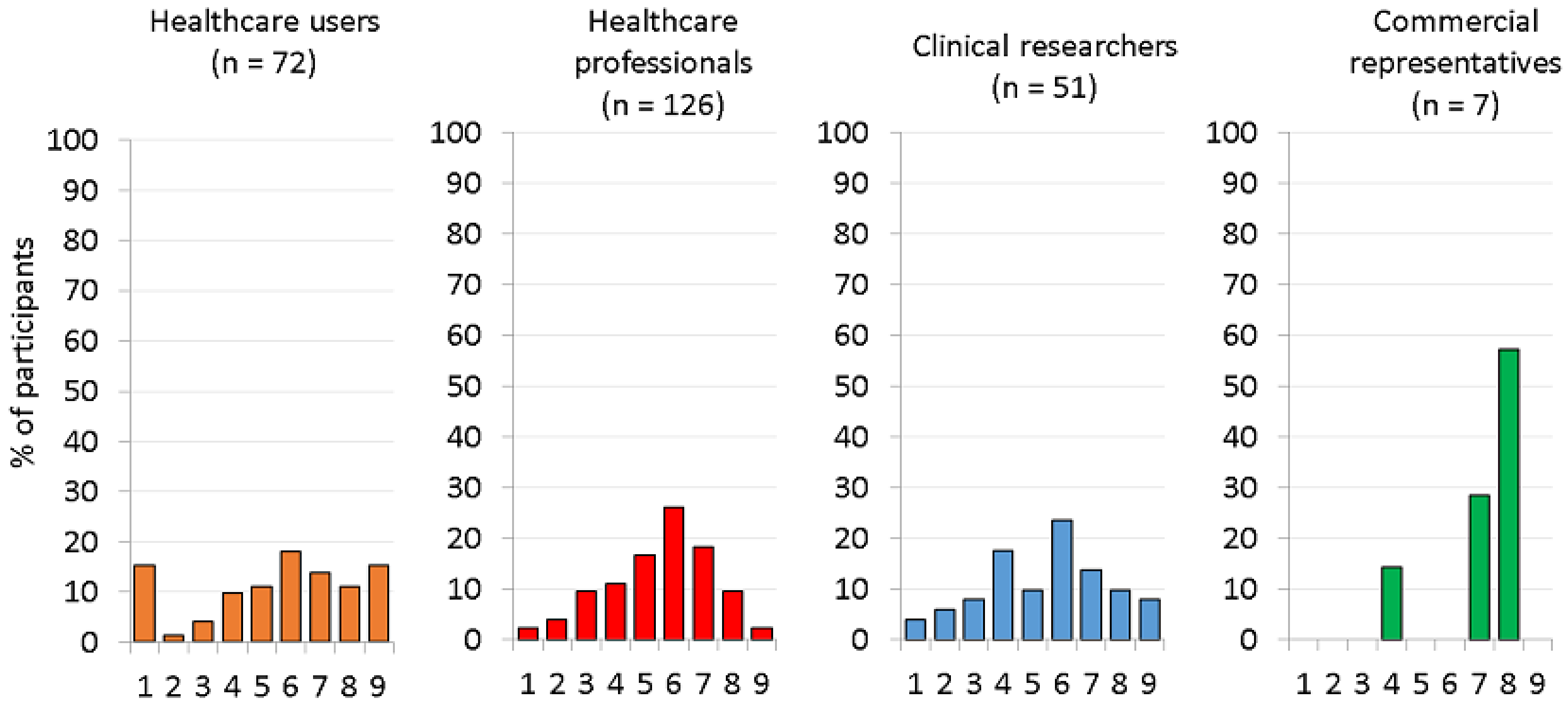

Domain Category:
Physical effects

NIHR | National Institute for Health Research

Rating scale:

1	2	3	4	5	6	7	8	9
Not at all important			*Important but not critical*			*Critical*		

32. HEARING-RELATED BRAIN CHANGES

Changes in brain structure or function associated with hearing loss

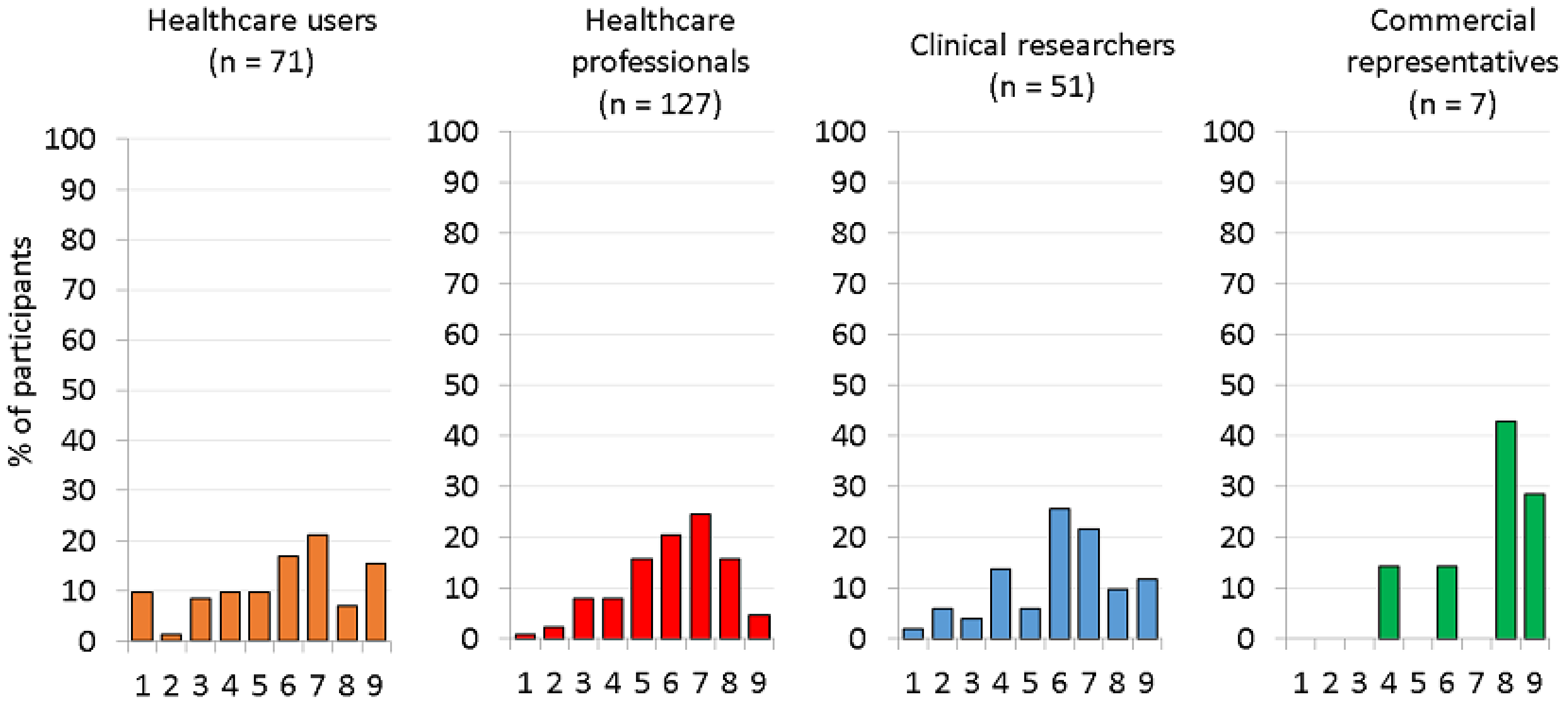

Domain Category:
Physical effects

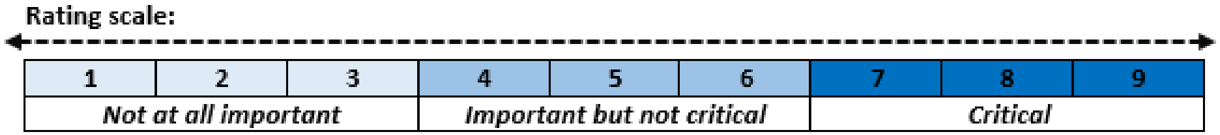

33. SELF-STIGMA

Negative perception of yourself due to your hearing loss and feeling stigmatised for using a hearing aid

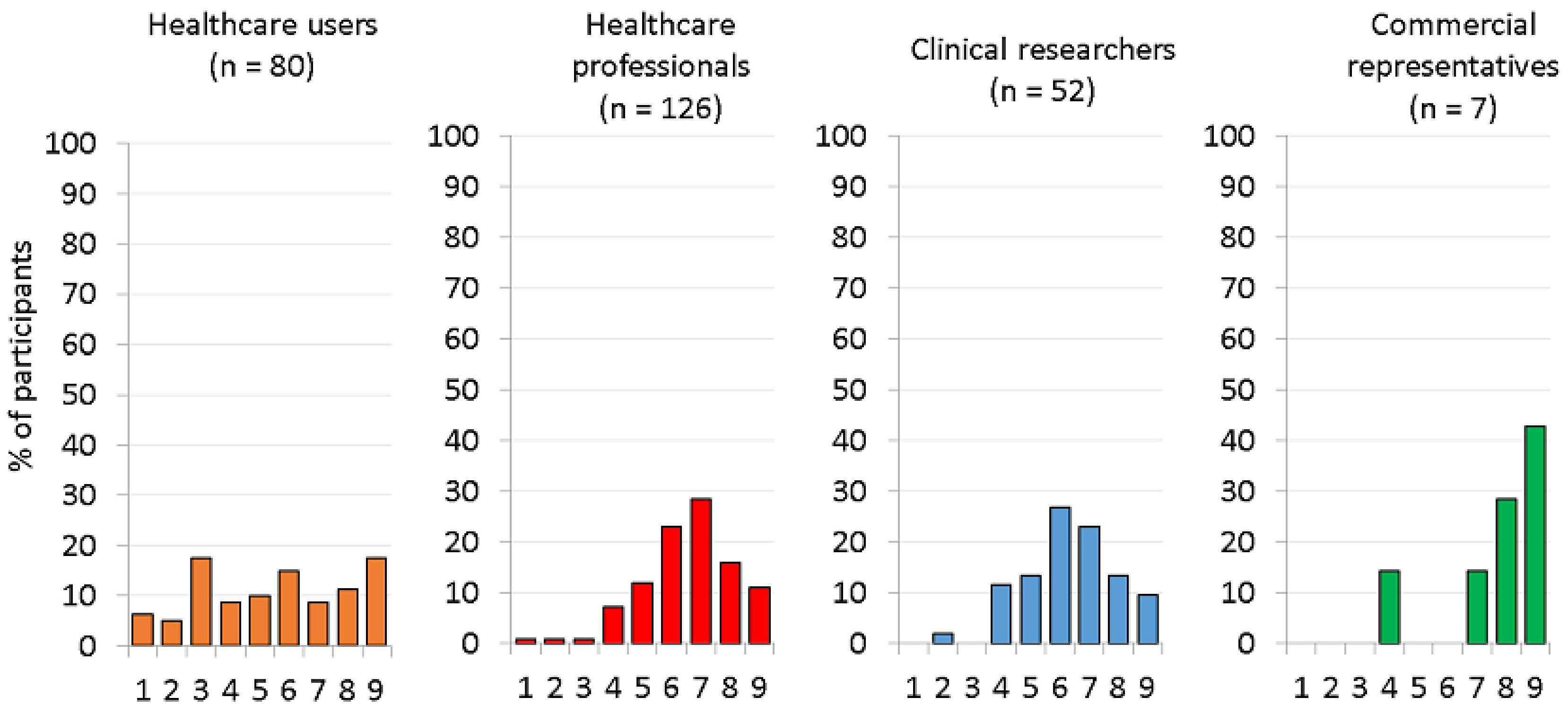

Domain Category: Self

Rating scale:

1	2	3	4	5	6	7	8	9
Not at all important			*Important but not critical*			*Critical*		

NIHR | National Institute for Health Research

34. SELF-IMAGE

Feeling incomplete or incapable because you are unable to do all the things that you want to do

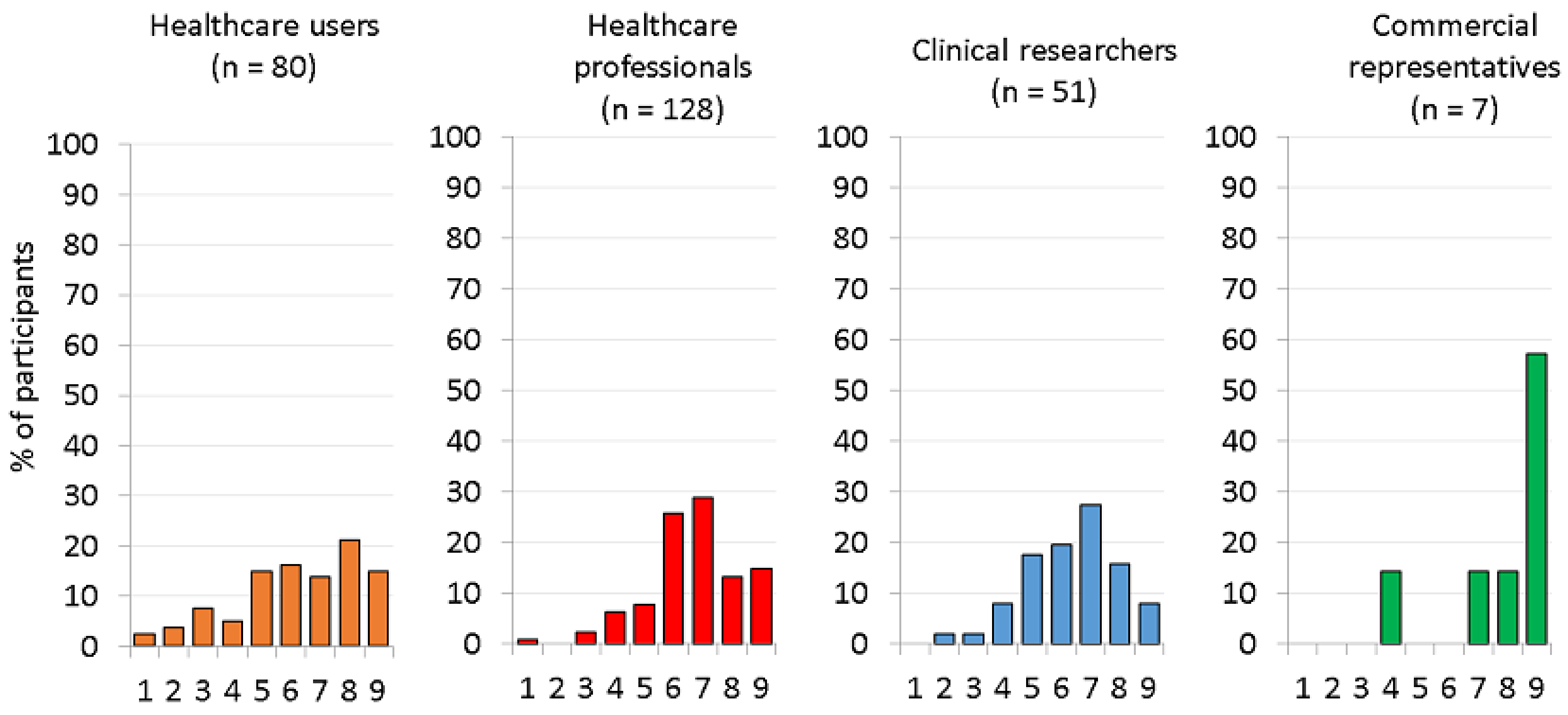

Domain Category: Self

Rating scale:

1	2	3	4	5	6	7	8	9
Not at all important			*Important but not critical*			*Critical*		

NIHR | National Institute for Health Research

35. PERSONAL SAFETY

How your hearing loss effects your awareness of potential hazards and threats in your daily life (for example; moving traffic; hazards at the workplace) and those you may not be able to see or hear (for example; other people behind you)

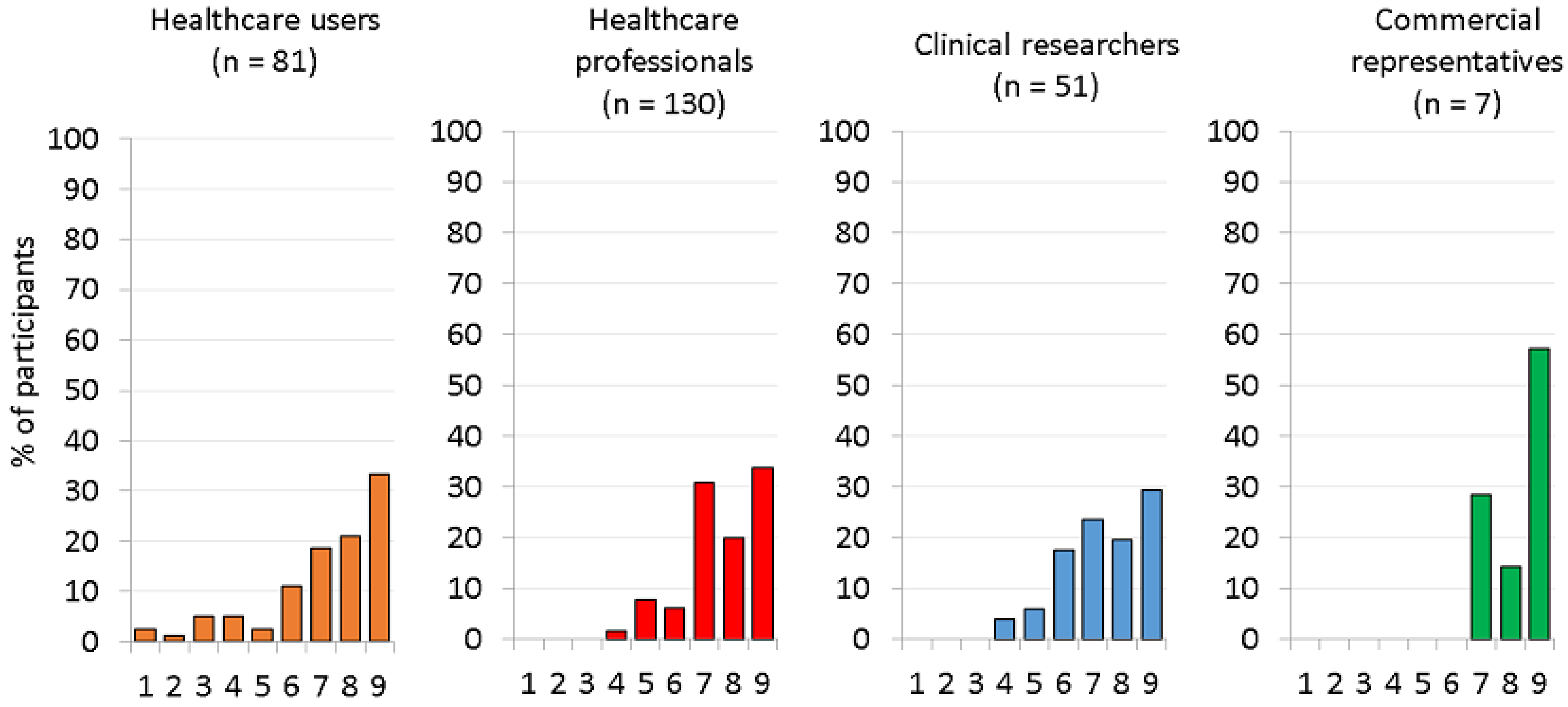

Domain Category: Self

NIHR | National Institute for Health Research

Rating scale:

1	2	3	4	5	6	7	8	9
Not at all important			*Important but not critical*			*Critical*		

36. PROTECTING YOUR HEARING

Making a conscious decision to avoid loud sounds or other risks to your hearing; or taking steps to protect your hearing

CROSSSD

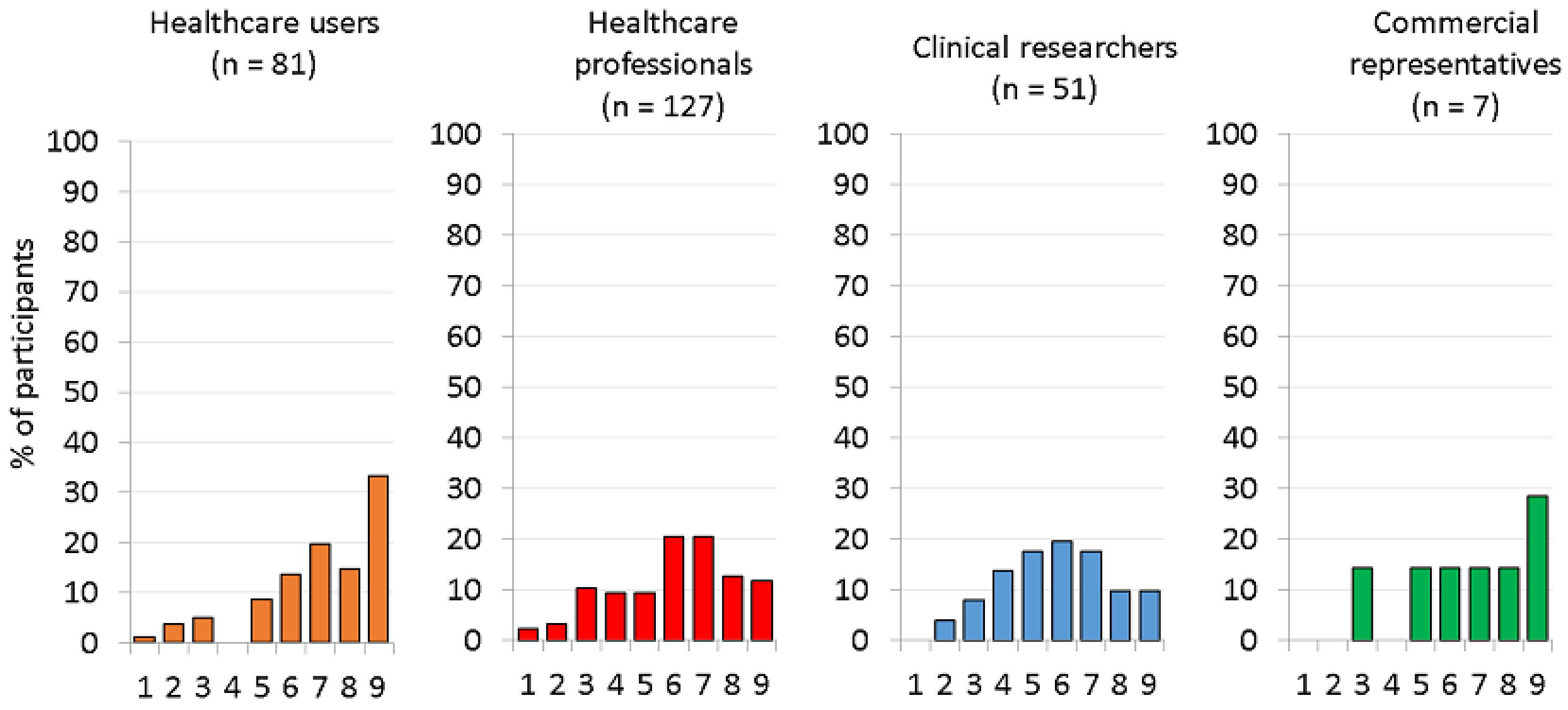

Domain Category: Self

NIHR | National Institute for Health Research

Rating scale:

1	2	3	4	5	6	7	8	9
Not at all important			*Important but not critical*			*Critical*		

37. LOUDNESS

How 'loud' a sound seems to you

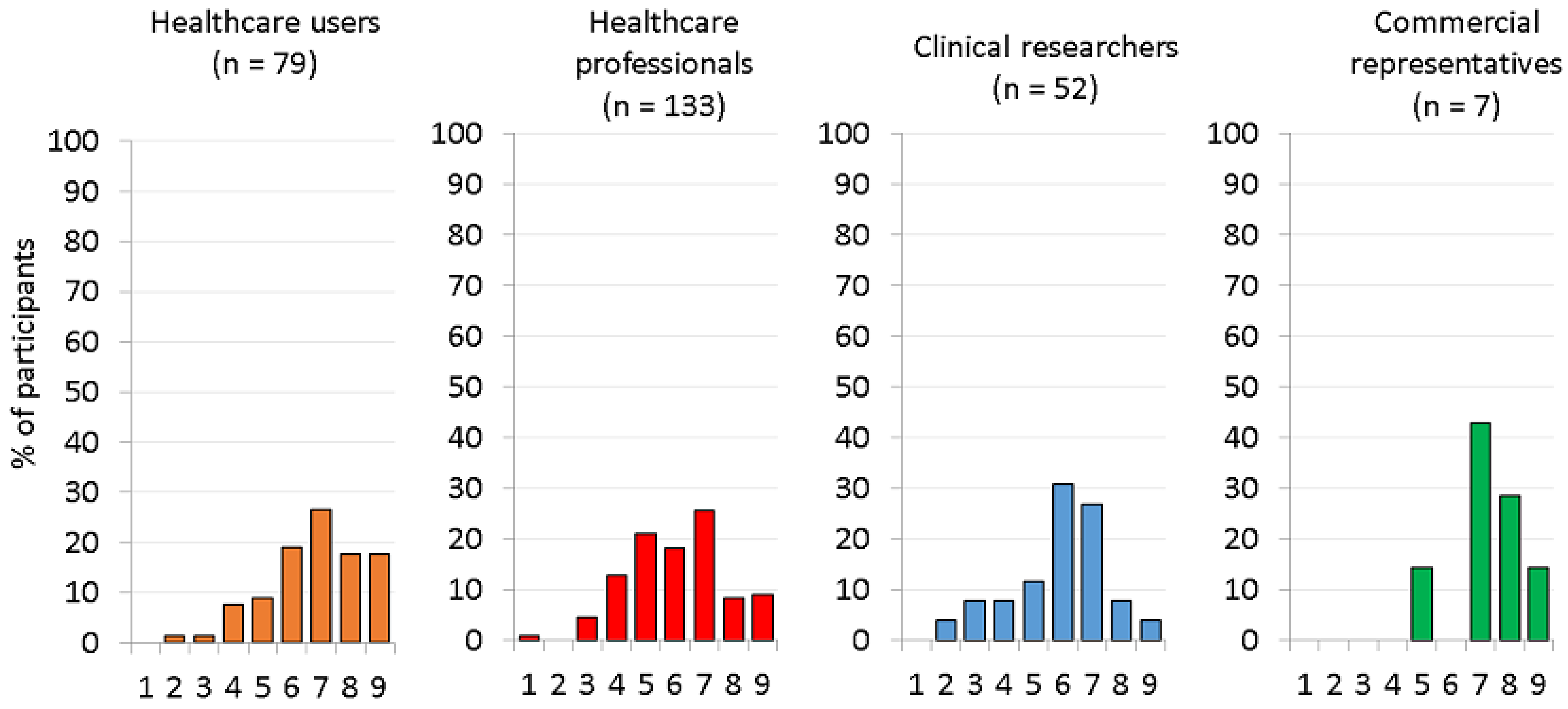

Domain Category:
Sound quality

Rating scale:

1	2	3	4	5	6	7	8	9
Not at all important			*Important but not critical*			*Critical*		

NIHR | National Institute for Health Research

38. FULLNESS

How 'full' a sound seems to you. This can also be described as the 'richness'; 'warmth' or 'depth' of a sound

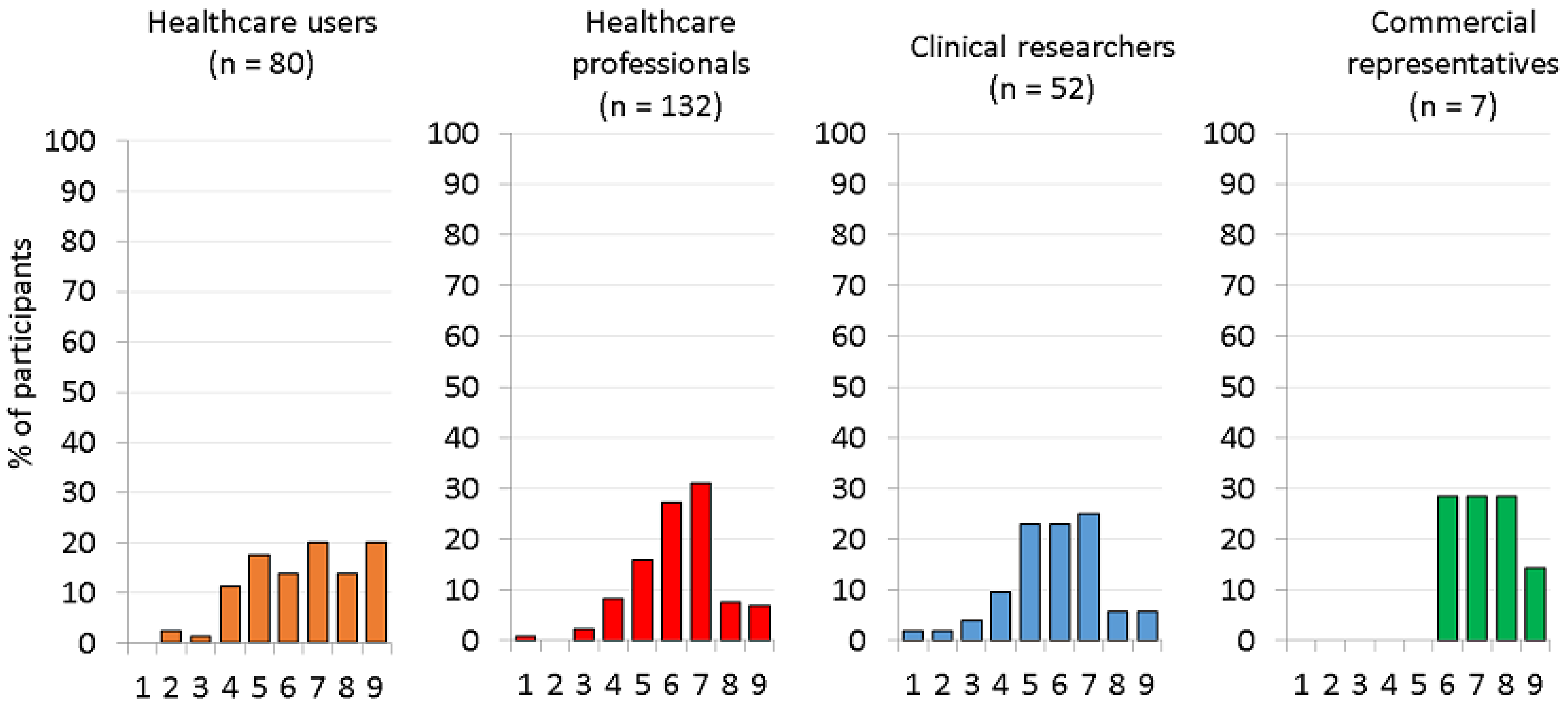

Domain Category:
Sound quality

NIHR | National Institute for Health Research

Rating scale:

1	2	3	4	5	6	7	8	9
Not at all important			*Important but not critical*			*Critical*		

39. CLARITY

How 'clear' a sound seems to you

CROSSSD

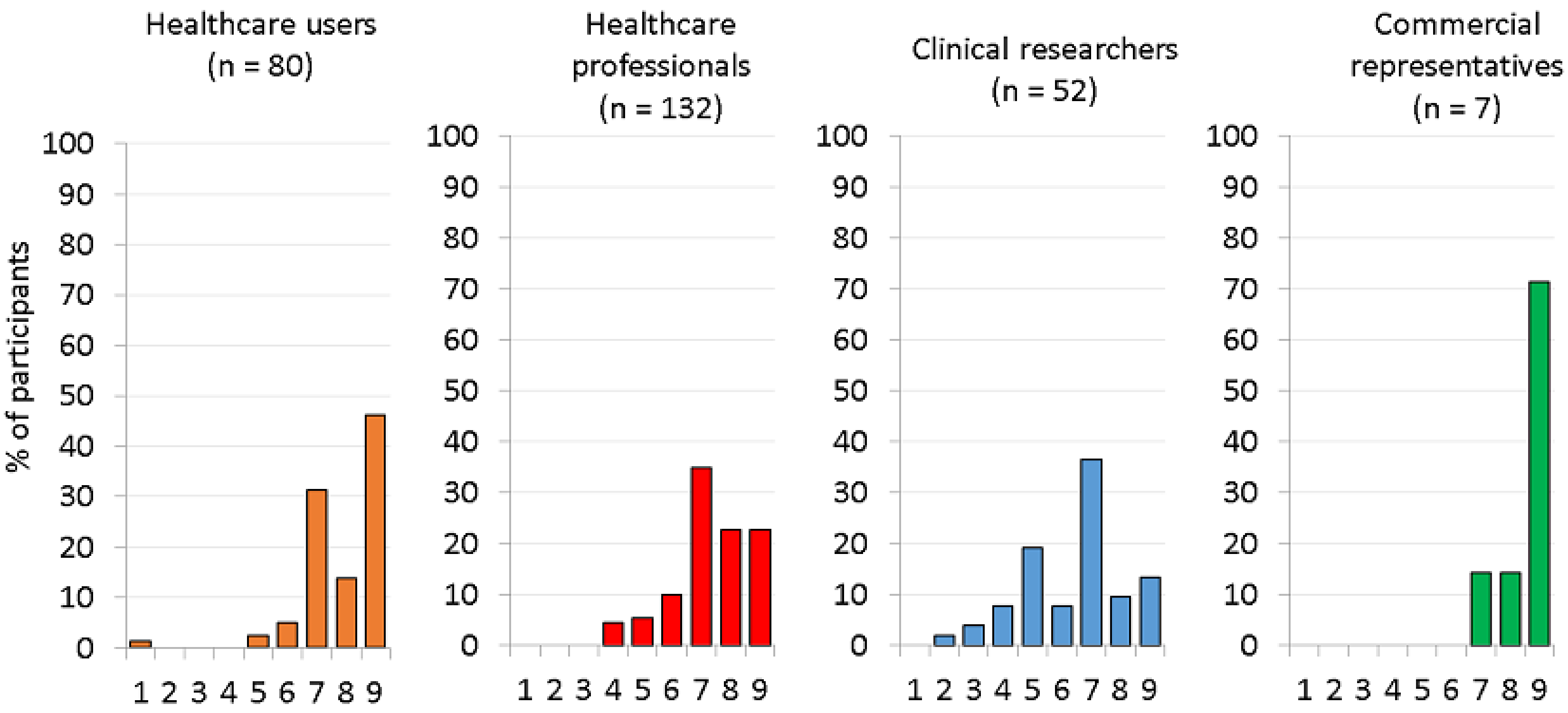

Domain Category:
Sound quality

NIHR | National Institute for Health Research

Rating scale:

1	2	3	4	5	6	7	8	9
Not at all important			*Important but not critical*			*Critical*		

40. TINNITUS AWARENESS

Noticing the sound of tinnitus is there

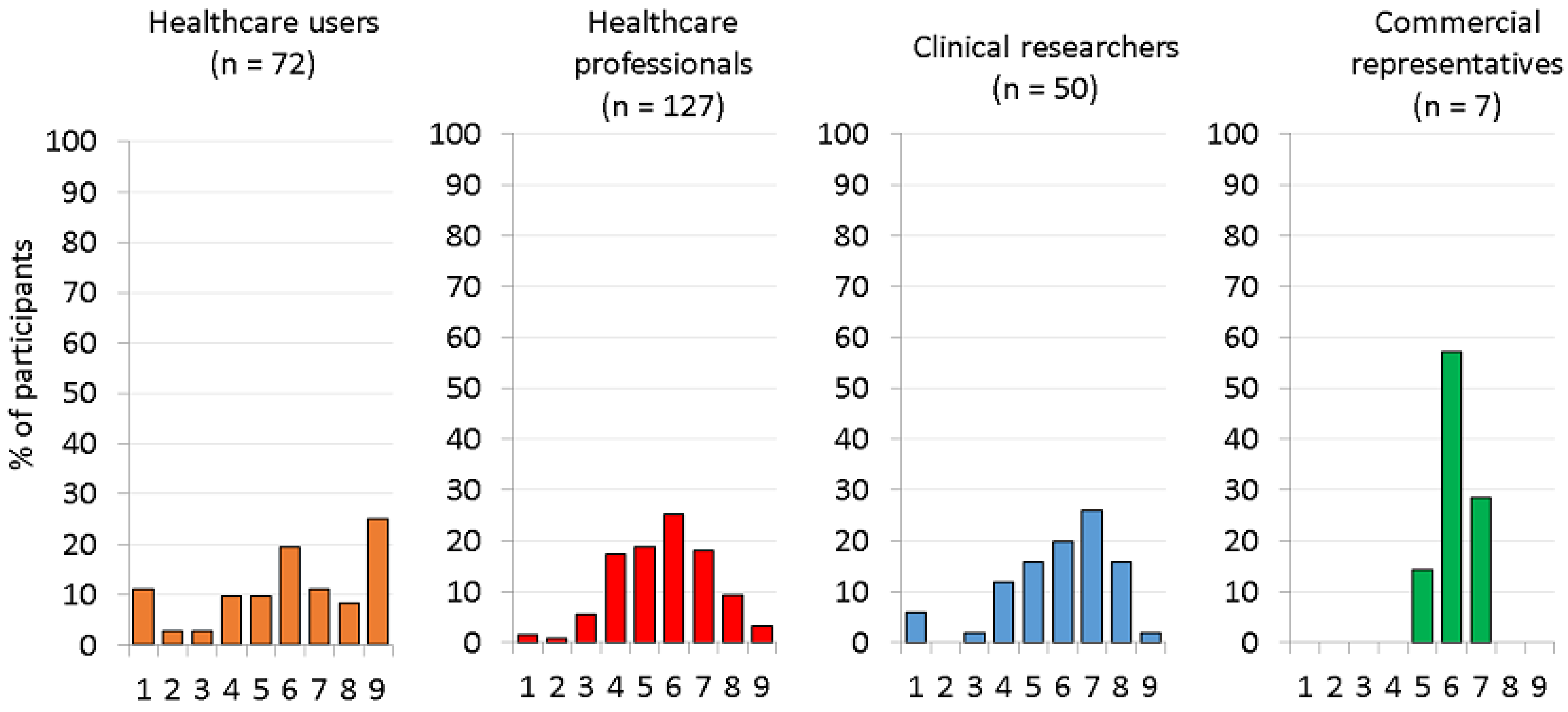

Domain Category: Tinnitus

Rating scale:

1	2	3	4	5	6	7	8	9
Not at all important			*Important but not critical*			*Critical*		

NIHR | National Institute for Health Research

41. TINNITUS INTRUSIVENESS

Being acutely aware of the sounds of tinnitus; feeling that it is invading your life or your personal space; changing your thoughts or actions and negatively impacting on your life

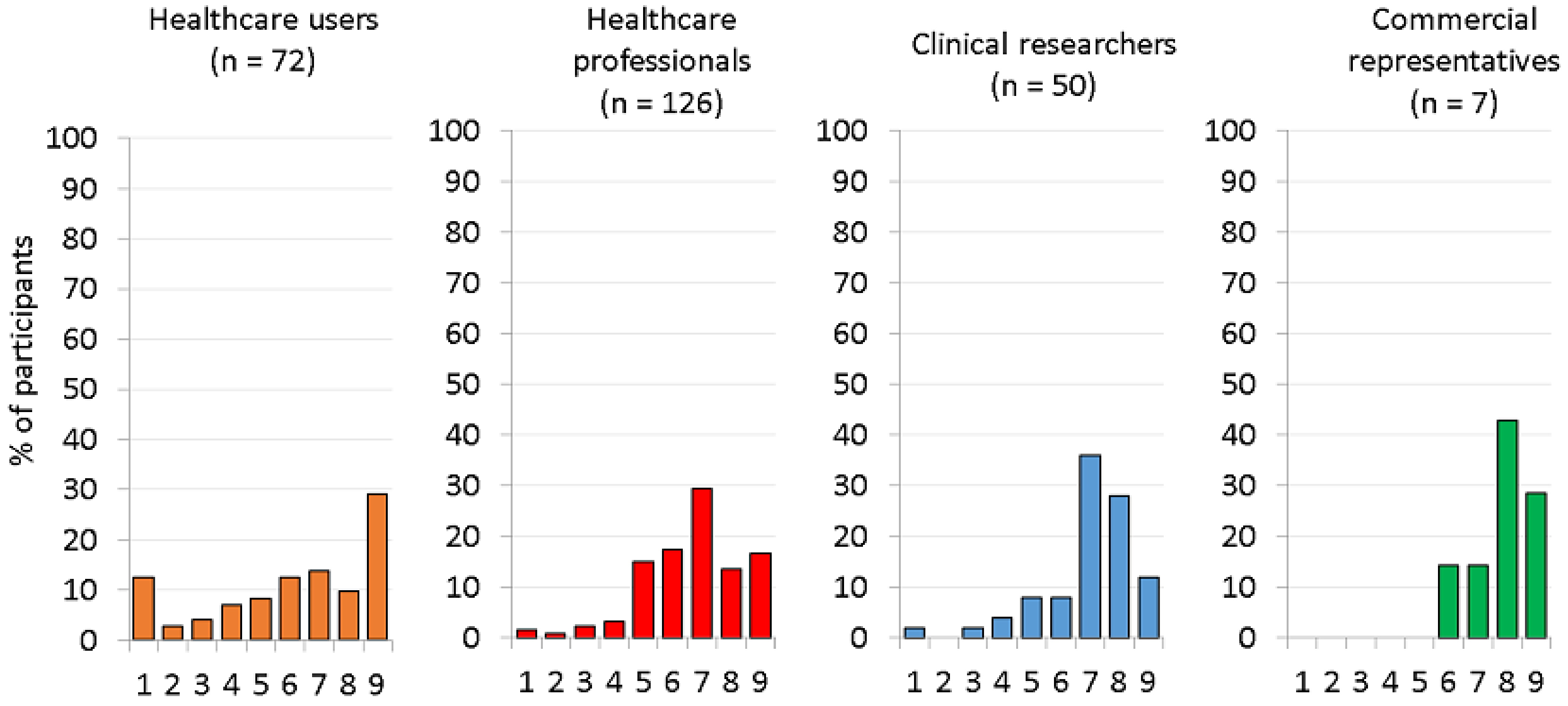

Domain Category: Tinnitus

Rating scale:

1	2	3	4	5	6	7	8	9
Not at all important			*Important but not critical*			*Critical*		

NIHR | National Institute for Health Research

42. TINNITUS LOUDNESS

How loud your tinnitus sounds

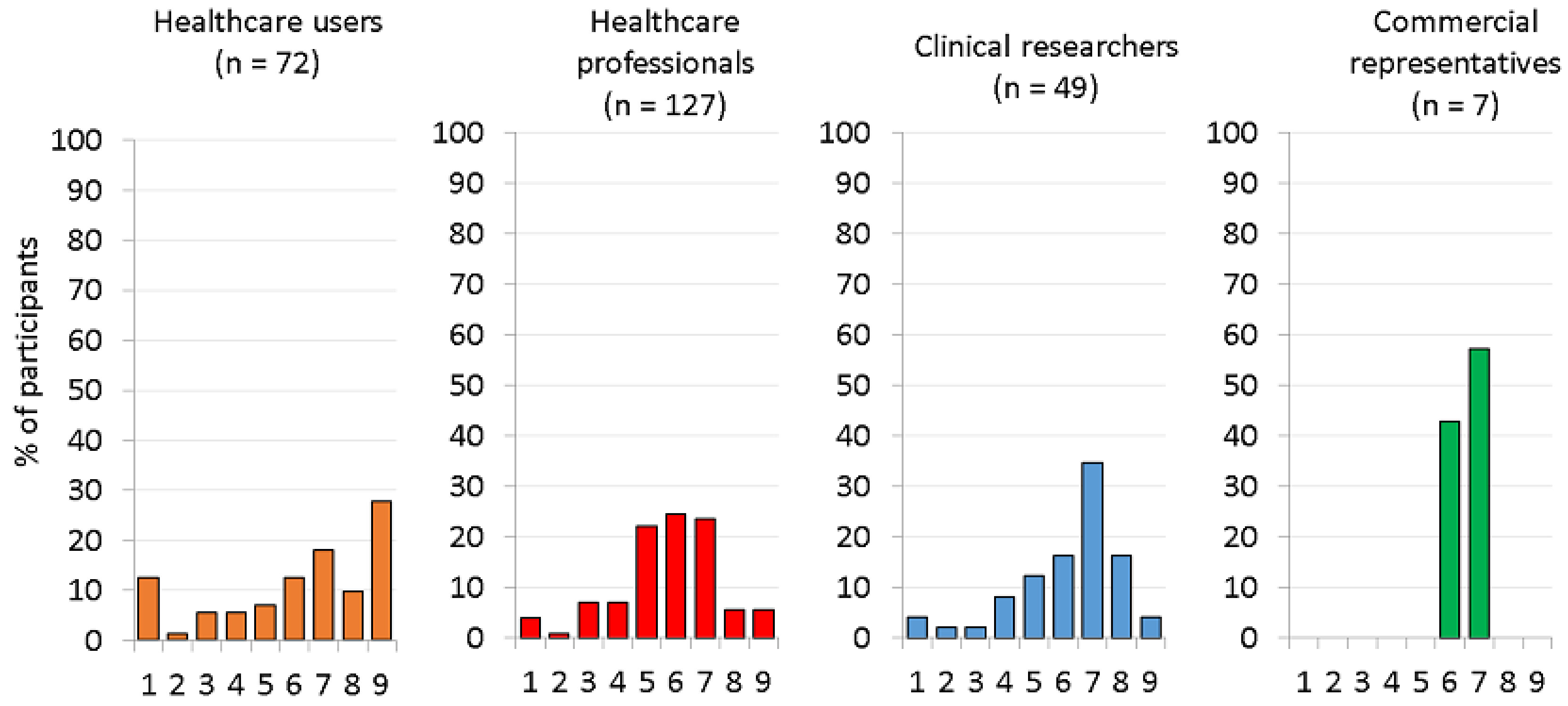

Domain Category: Tinnitus

Rating scale:

1	2	3	4	5	6	7	8	9
Not at all important			*Important but not critical*			*Critical*		

43. TINNITUS PITCH

Whether your tinnitus has a note-like quality;
for example high pitch like whistling or low pitch like humming

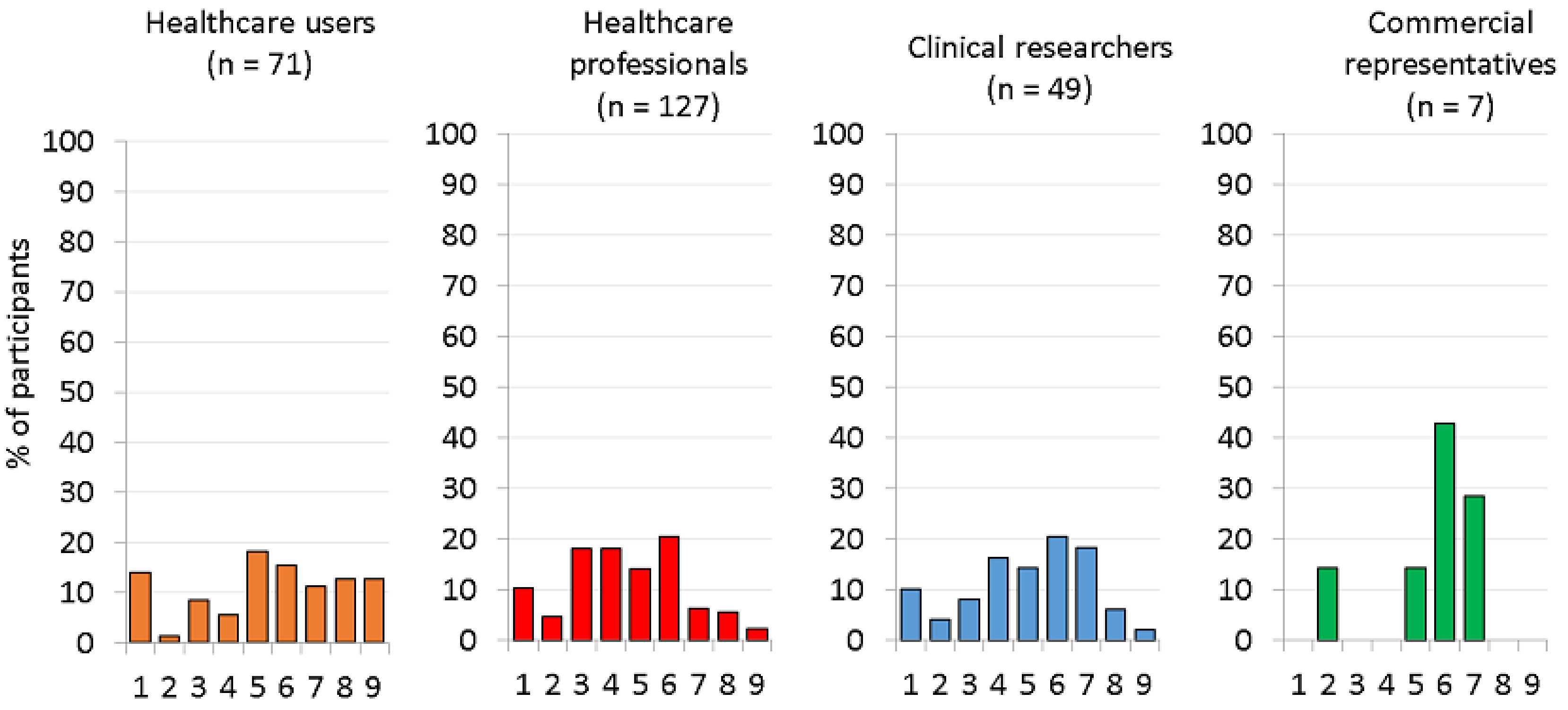

Domain Category: Tinnitus

Rating scale:

1	2	3	4	5	6	7	8	9
Not at all important			*Important but not critical*			*Critical*		

NIHR | National Institute for Health Research

44. TINNITUS QUALITY

What type of sound is heard
(for example; hissing; buzzing; ringing; whistling etc)

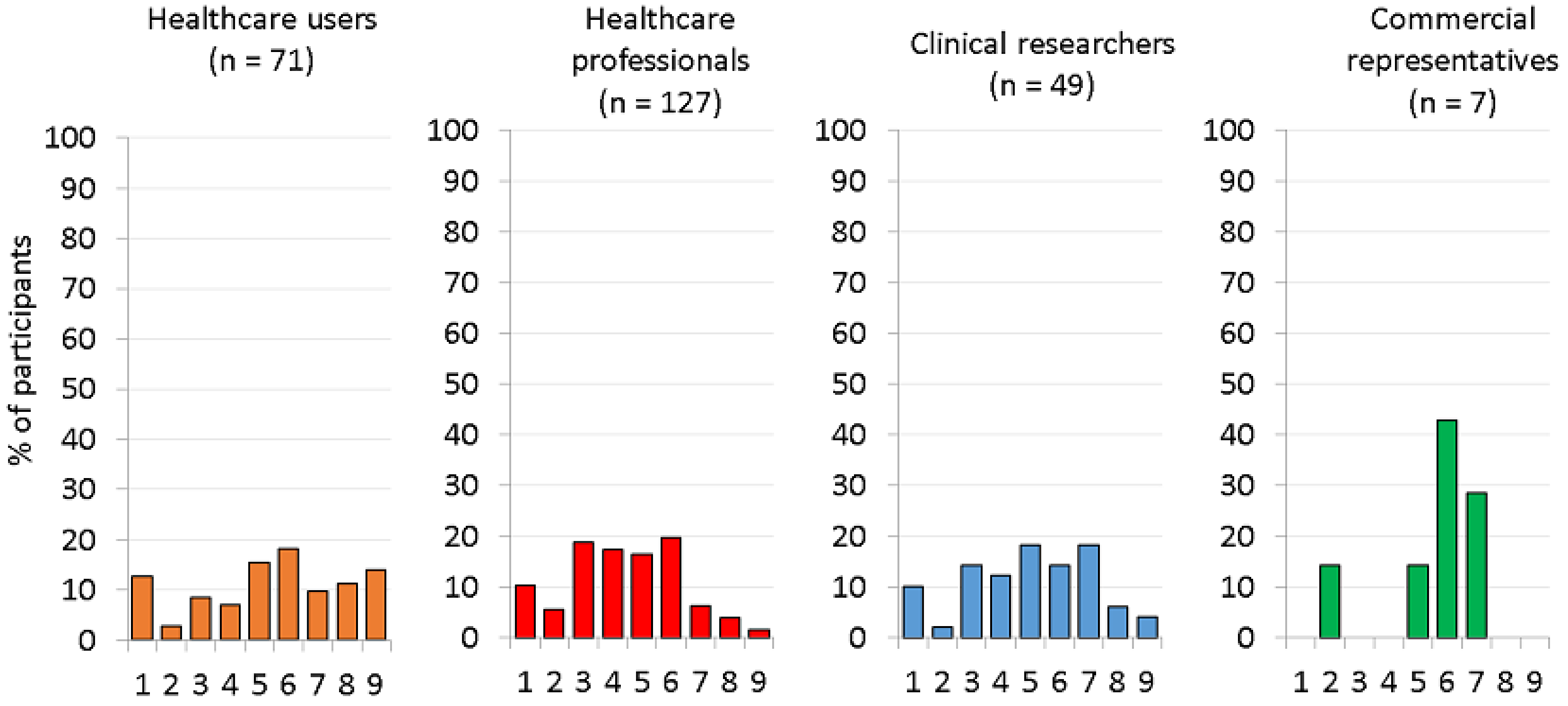

Domain Category: Tinnitus

Rating scale:

1	2	3	4	5	6	7	8	9
Not at all important			*Important but not critical*			*Critical*		

e-Delphi Round 2 outcome domain rating

CROSSSD

CORE REHABILITATION OUTCOME SET for SINGLE SIDED DEAFNESS

Identifying what is critical and important to measure when evaluating hearing interventions for adults with Single Sided Deafness (SSD)

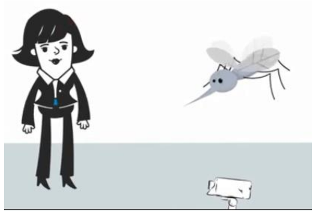

1. AVERSION TO LOUD SOUNDS

CROSSSD

Feeling uncomfortable when listening to loud sounds

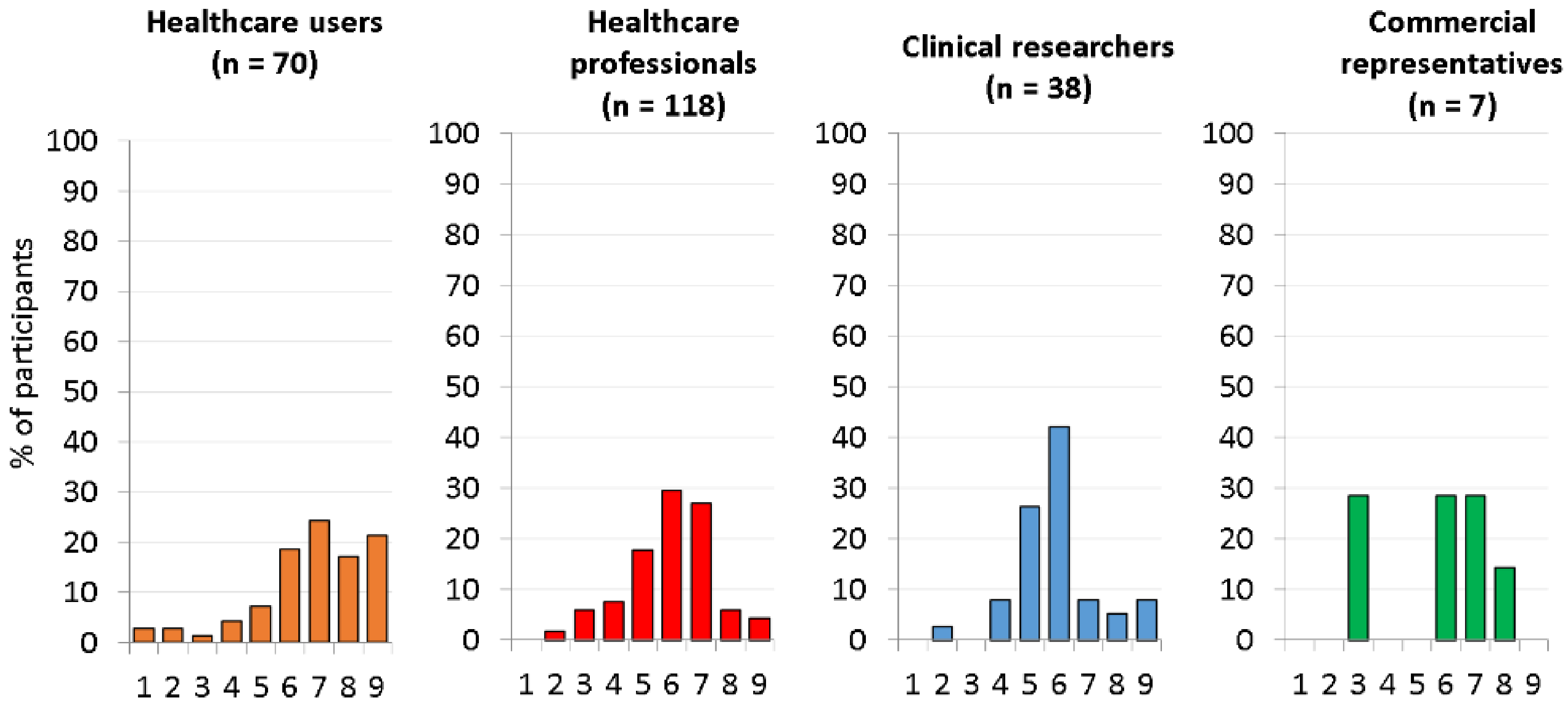

Domain Category:
Psychological effects

National Institute for Health Research

Rating scale:

1	2	3	4	5	6	7	8	9
Not at all important			*Important but not critical*			*Critical*		

2. DISCOMFORT IN LISTENING SITUATIONS

Finding yourself in listening situations that you feel you can't adequately control (for example; when you can't choose a favourable listening position); or situations in which you don't feel comfortable (for example when interacting with people who don't know you have a hearing loss)

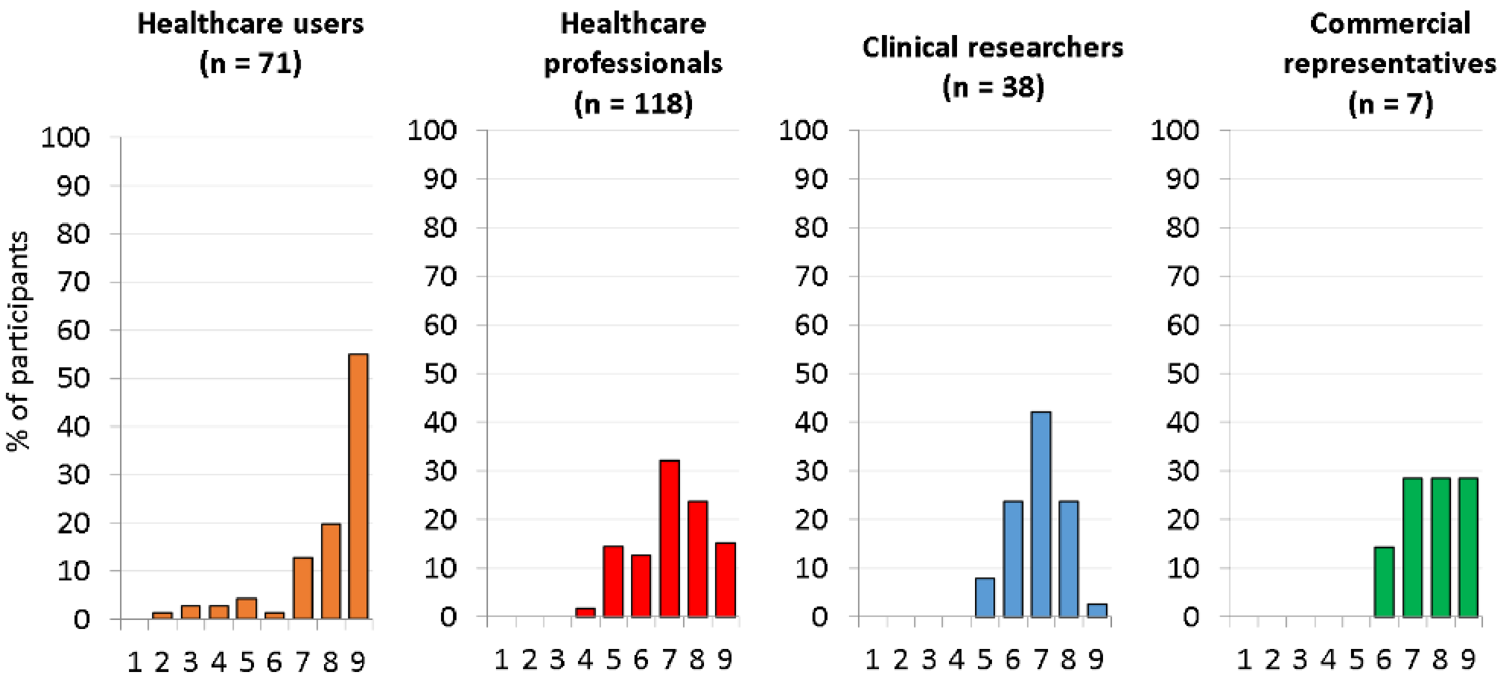

Domain Category:
Psychological effects

NIHR | National Institute for Health Research

Rating scale:

1	2	3	4	5	6	7	8	9
Not at all important			*Important but not critical*			*Critical*		

3. EMOTIONAL DISTRESS

A negative unpleasant emotional reaction which may include fear; anger; frustration; anxiety; and suffering

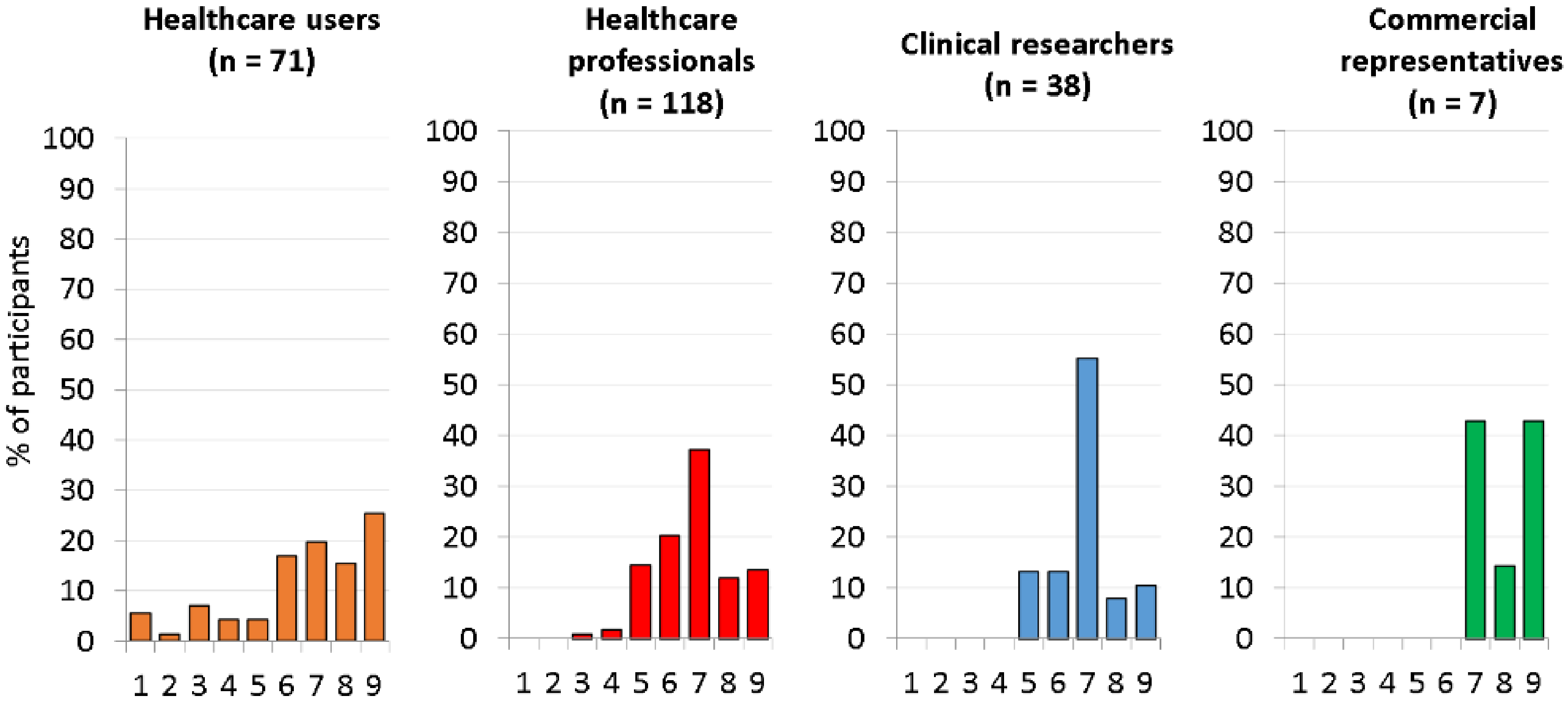

Domain Category:
Psychological effects

NIHR | National Institute for Health Research

Rating scale:

1	2	3	4	5	6	7	8	9
Not at all important			*Important but not critical*			*Critical*		

4. MOOD

General sense of well-being; ranging from feeling very low or negative to very positive

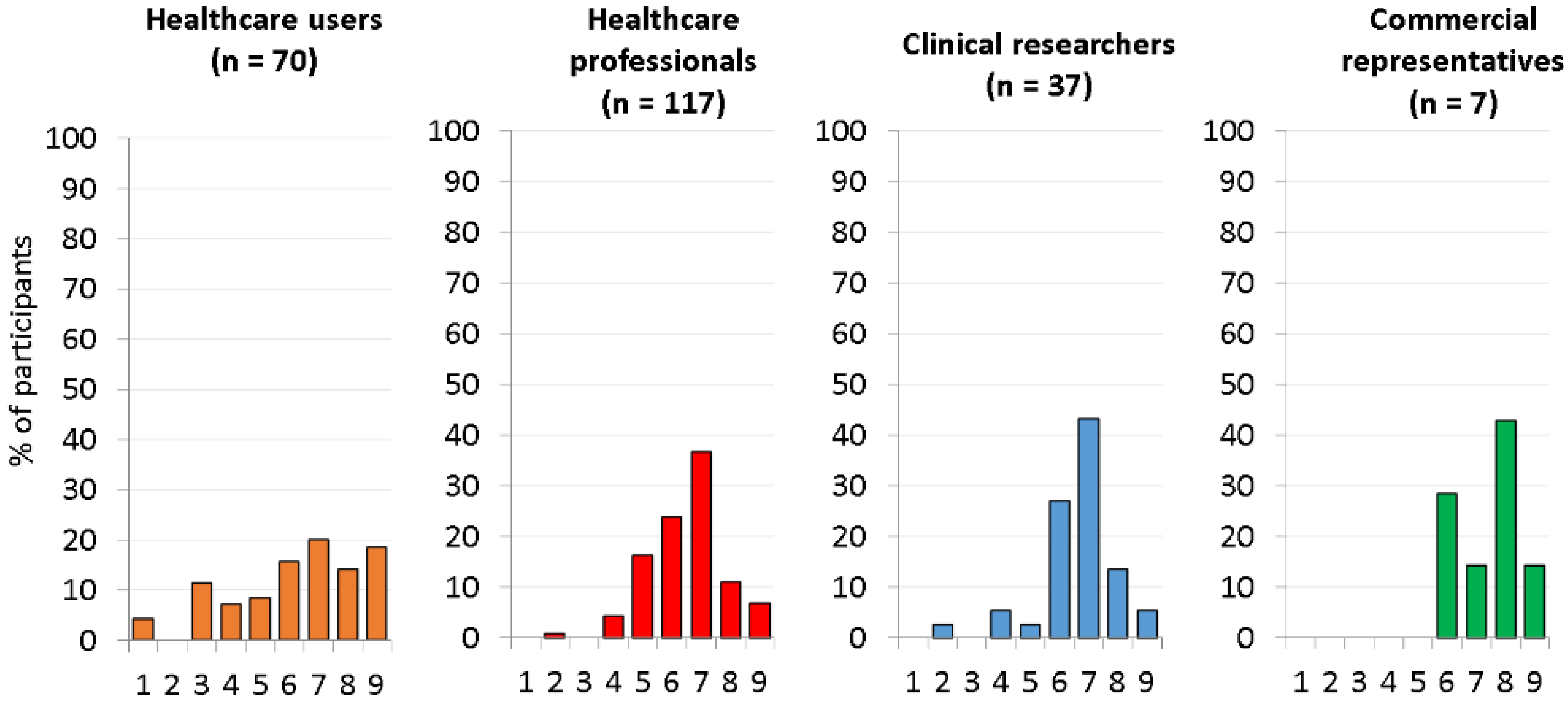

Domain Category:
Psychological effects

Rating scale:

1	2	3	4	5	6	7	8	9
Not at all important			*Important but not critical*			*Critical*		

National Institute for Health Research

5. MOTIVATION

A willingness to engage in challenging listening situations

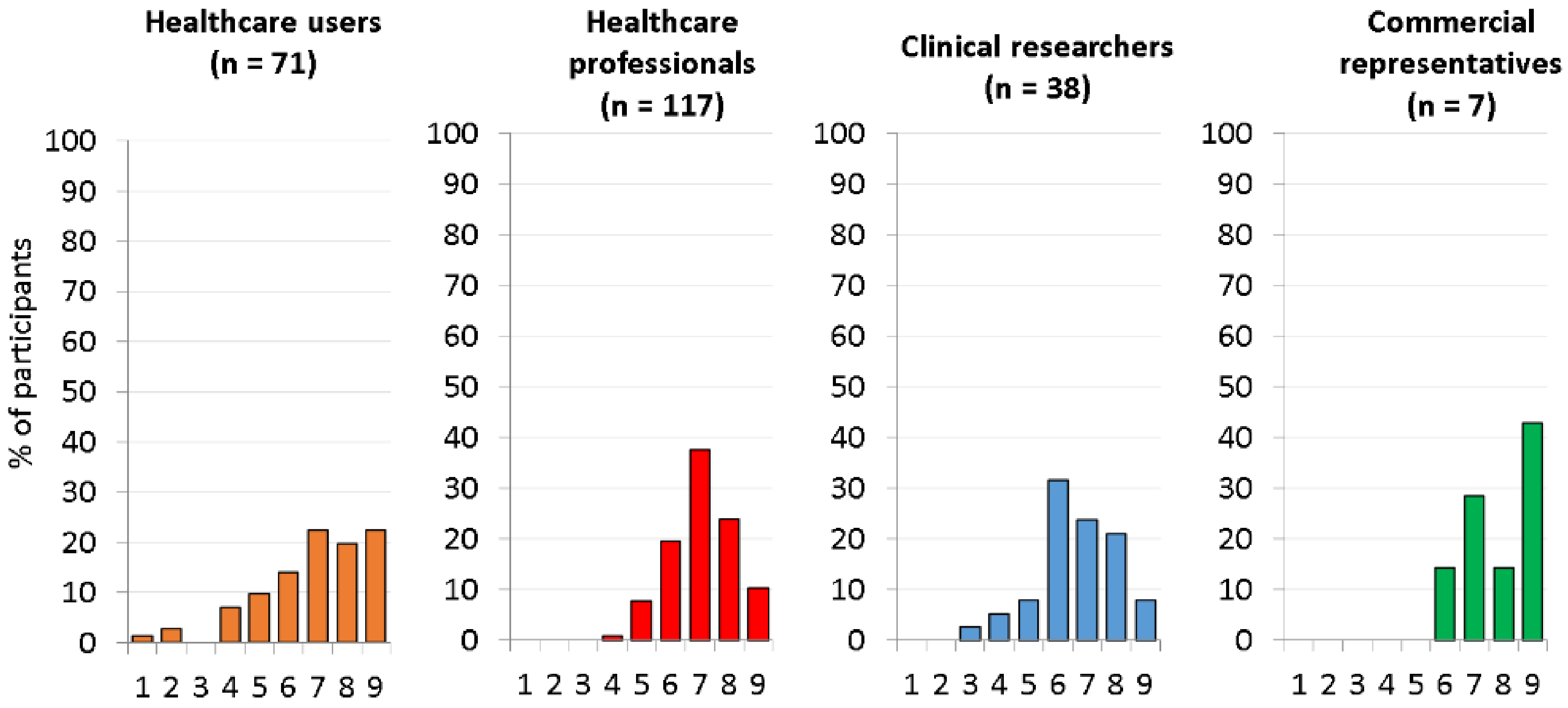

Domain Category:

Psychological effects

NIHR | National Institute for Health Research

Rating scale:

1	2	3	4	5	6	7	8	9
Not at all important			*Important but not critical*			*Critical*		

6. DISSATISFACTION WITH LIFE

Being unhappy because you feel you should be achieving or should have achieved more in your life

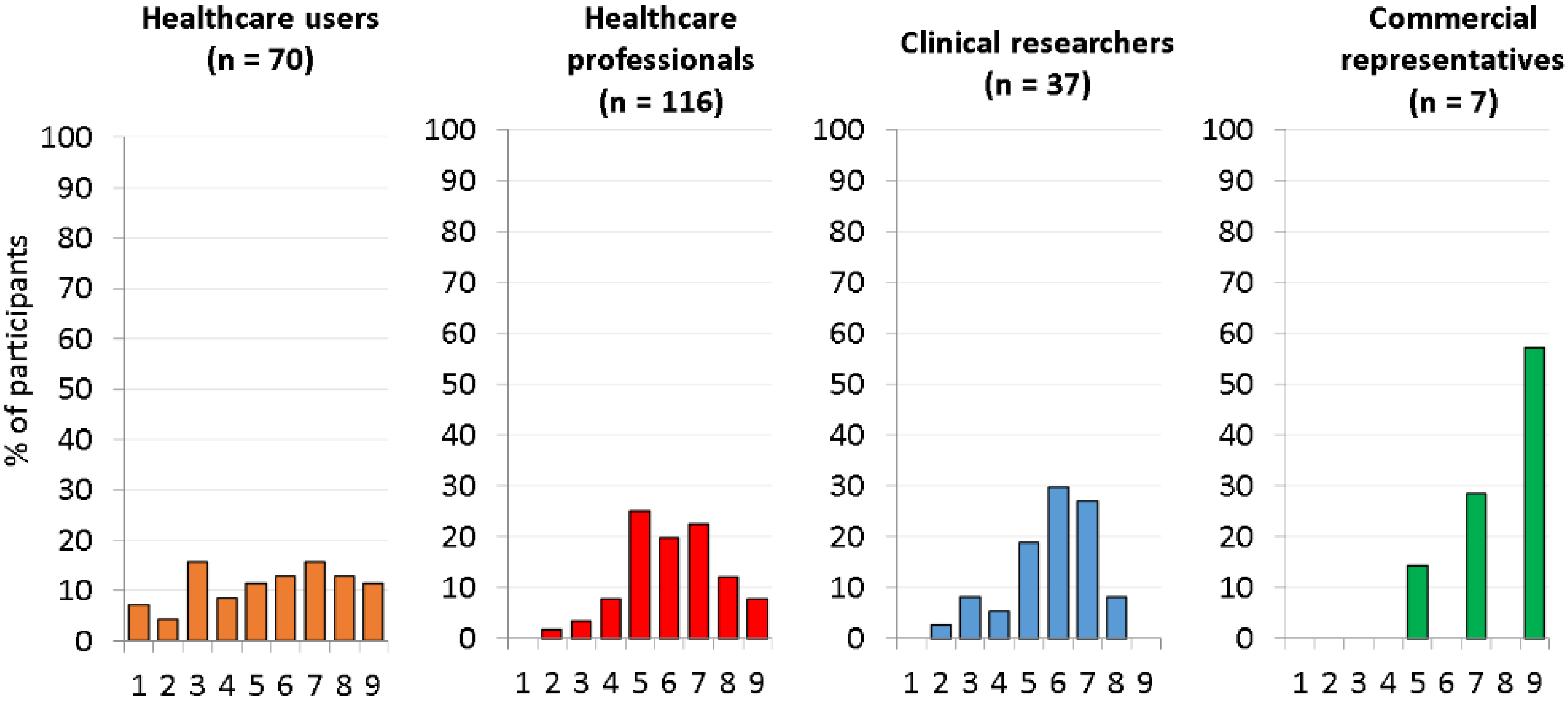

Domain Category:
Psychological effects

NIHR | National Institute for Health Research

Rating scale:

1	2	3	4	5	6	7	8	9
Not at all important			*Important but not critical*			*Critical*		

7. LISTENING EFFORT

CROSSSD

Exerting greater effort to listen and follow a conversation.
This might consequently lead to feelings of tiredness and fatigue; but those feelings would be a separate outcome domain

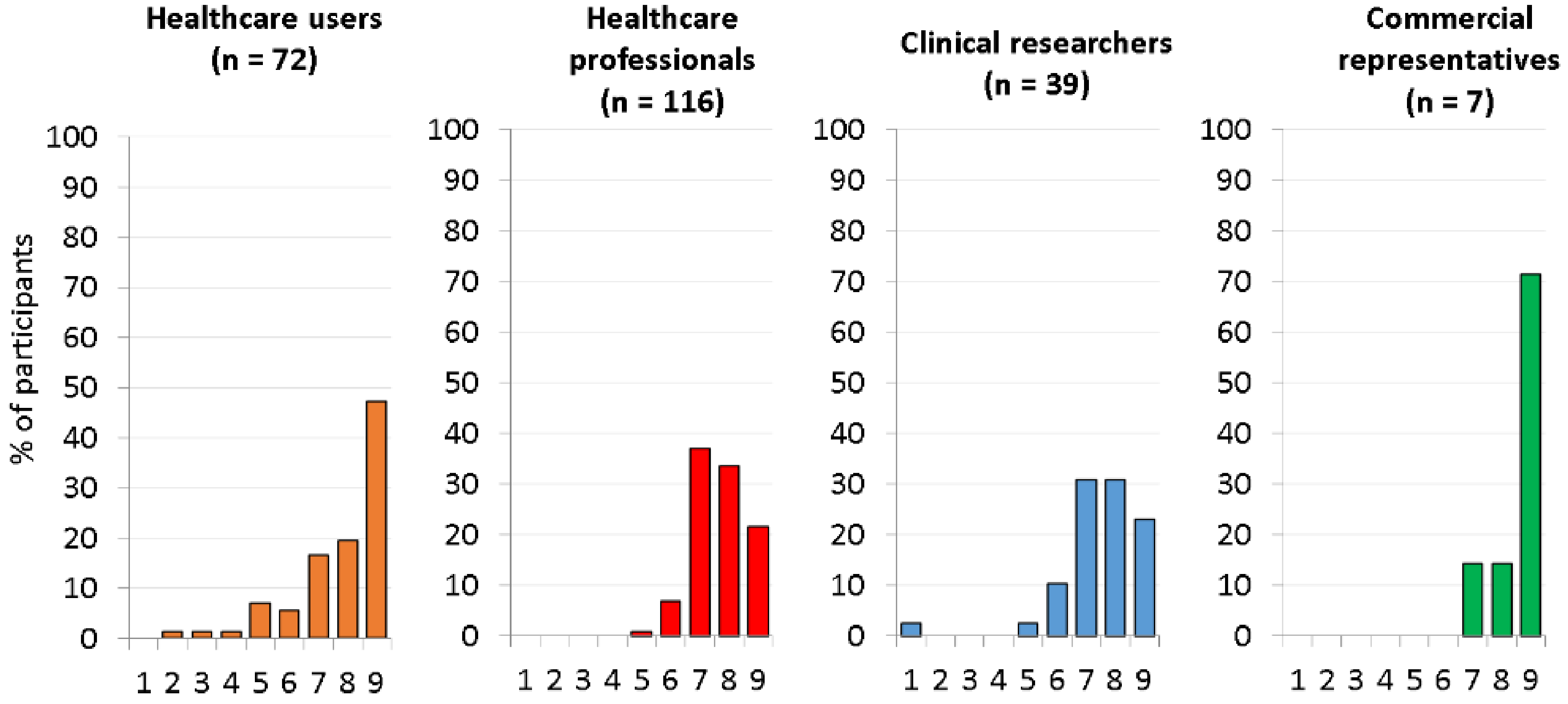

Domain Category:
Other effects

Rating scale:

1	2	3	4	5	6	7	8	9
Not at all important			*Important but not critical*			*Critical*		

NIHR | National Institute for Health Research

8. TREATMENT SATISFACTION

How the treatment meets your expectations or how pleased you are after receiving the treatment; or how likely you are to recommend the treatment

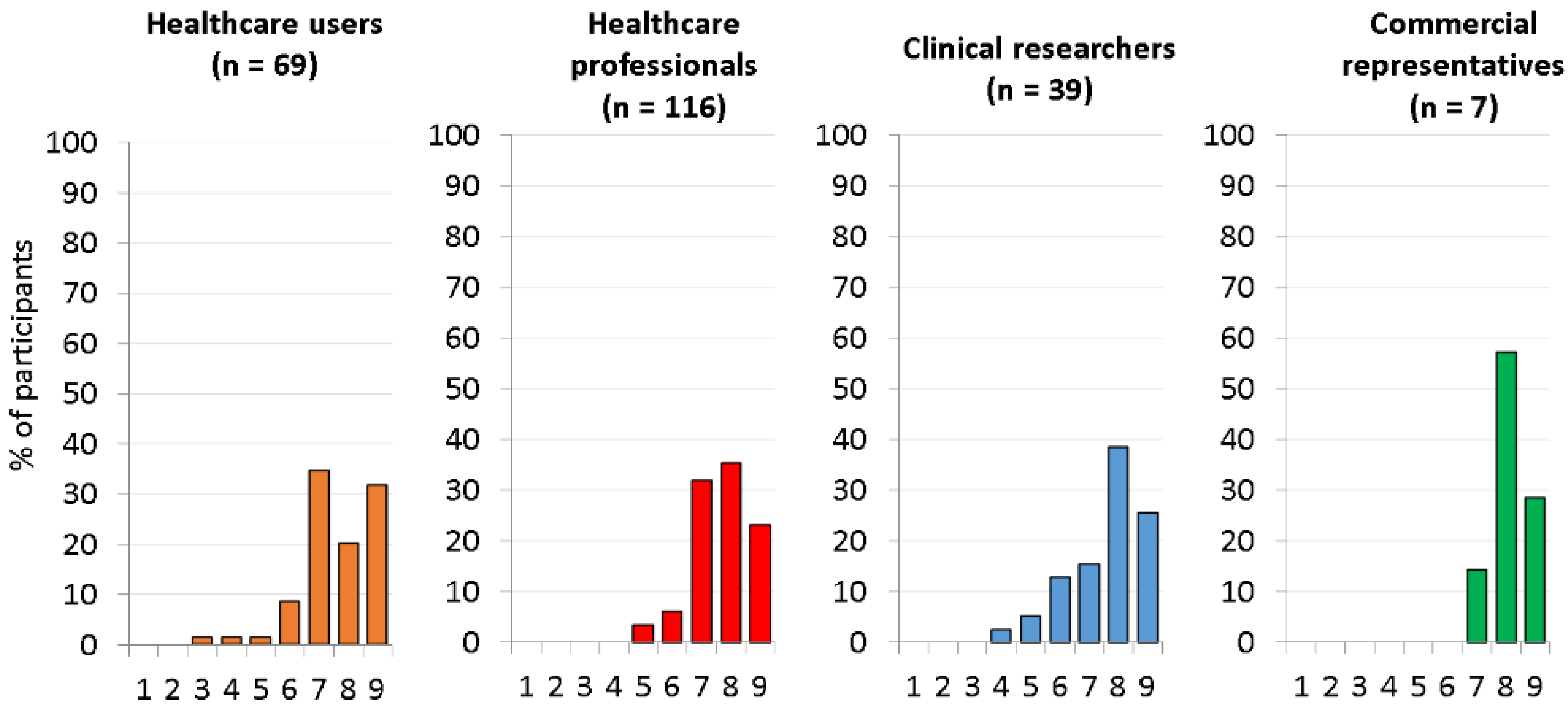

Domain Category: Factors related to the treatment being tested

Rating scale:

1	2	3	4	5	6	7	8	9
Not at all important			*Important but not critical*			*Critical*		

National Institute for Health Research

9. DEVICE USAGE

How you use the device
(for example; in what situations; for how long)

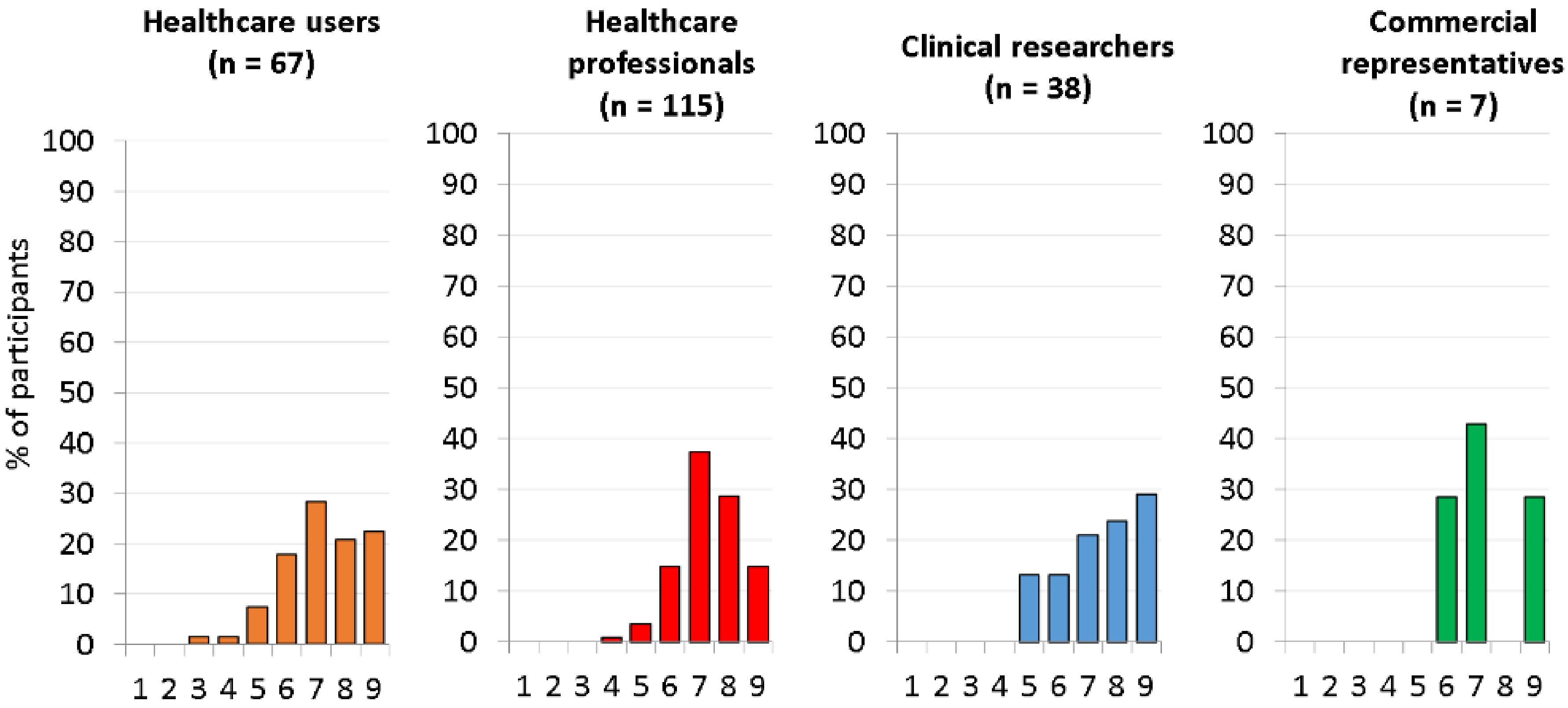

Domain Category: Factors related to the treatment being tested

National Institute for Health Research

Rating scale:

1	2	3	4	5	6	7	8	9
Not at all important			*Important but not critical*			*Critical*		

10. DEVICE MALFUNCTION

The device does not work as it should or it stops working

CROSSSD

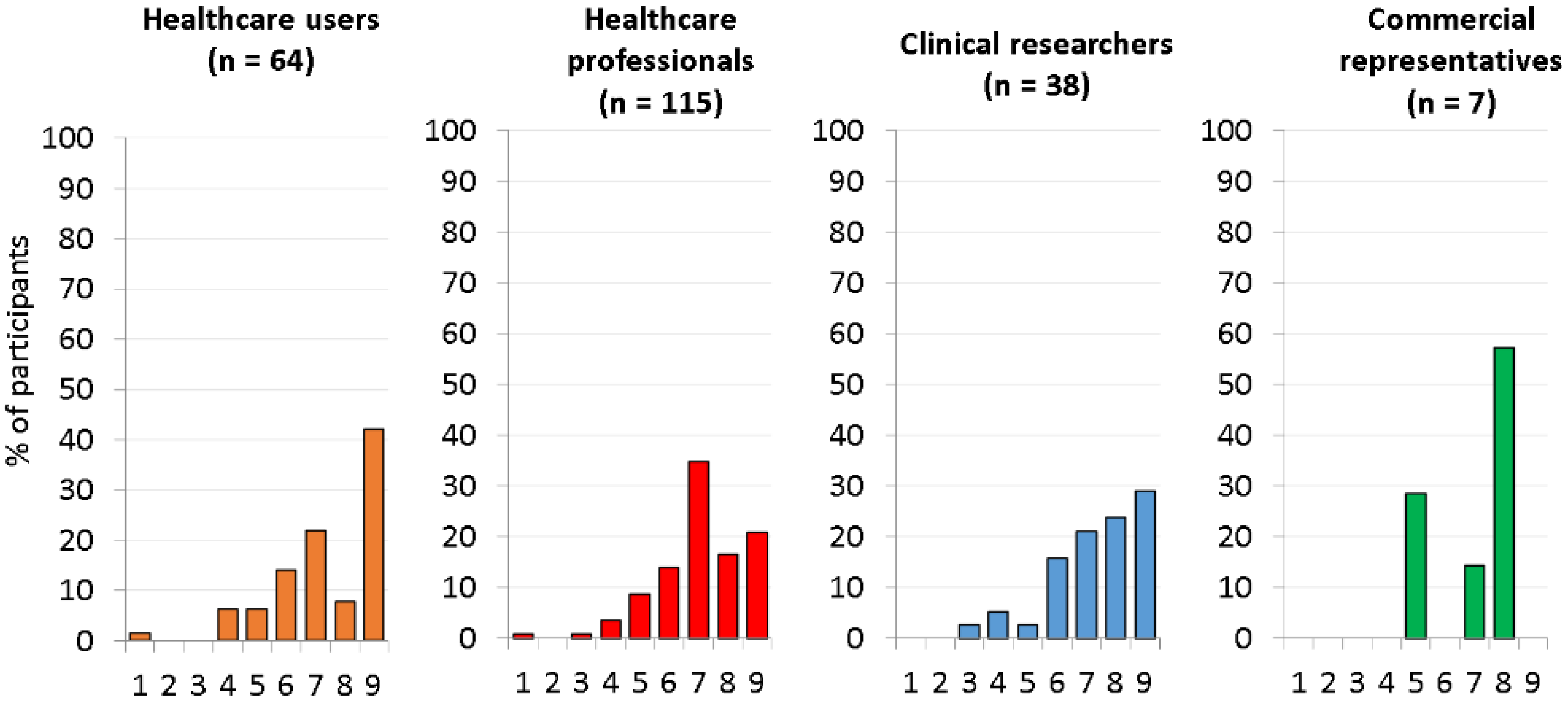

Domain Category: Factors related to the treatment being tested

NIHR | National Institute for Health Research

Rating scale:

1	2	3	4	5	6	7	8	9
Not at all important			*Important but not critical*			*Critical*		

11. ADVERSE EVENTS

Any bad or unexpected thing that happens during the time a treatment is being tested in a clinical trial

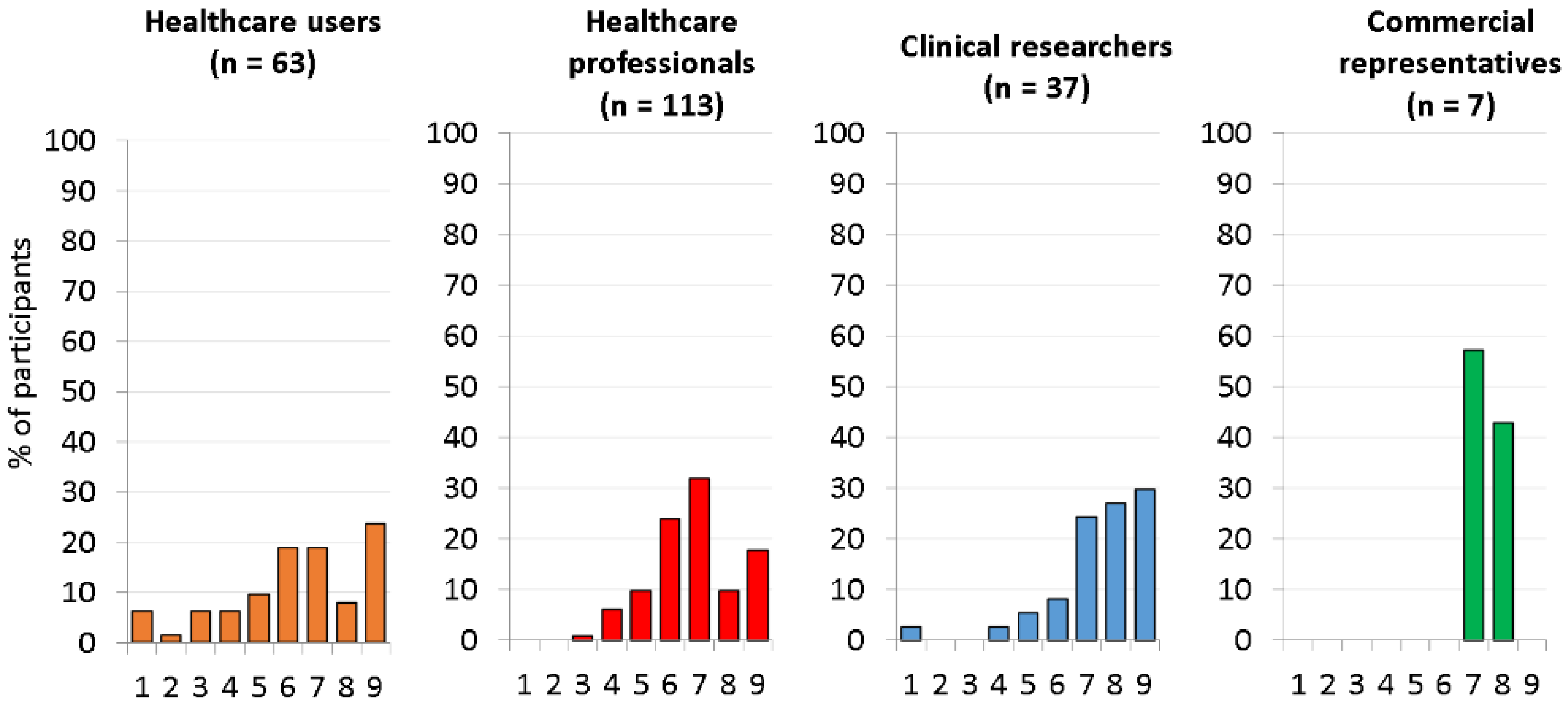

Domain Category: Factors related to the treatment being tested

Rating scale:

1	2	3	4	5	6	7	8	9
Not at all important			*Important but not critical*			*Critical*		

NIHR | National Institute for Health Research

12. AVOIDING SOCIAL SITUATIONS

Choosing not to go to particular social situations because of your hearing loss

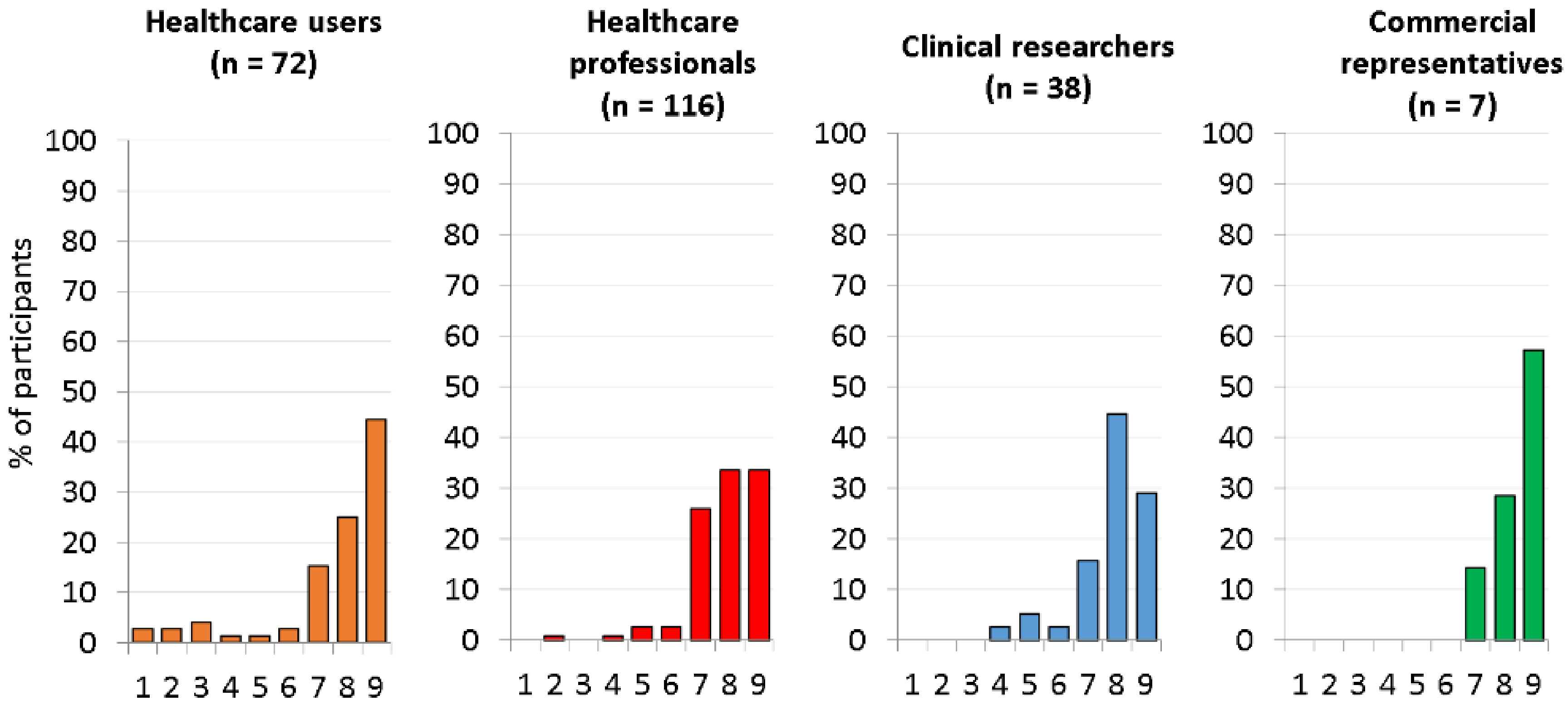

Domain Category: Health-related quality of life

Rating scale:

1	2	3	4	5	6	7	8	9
Not at all important			*Important but not critical*			*Critical*		

NIHR | National Institute for Health Research

13. IMPACT ON INDIVIDUAL ACTIVITIES

Effect of your hearing loss or your device on your choice to engage in individual activities (for example; travelling alone; swimming or watching TV / films / movies)

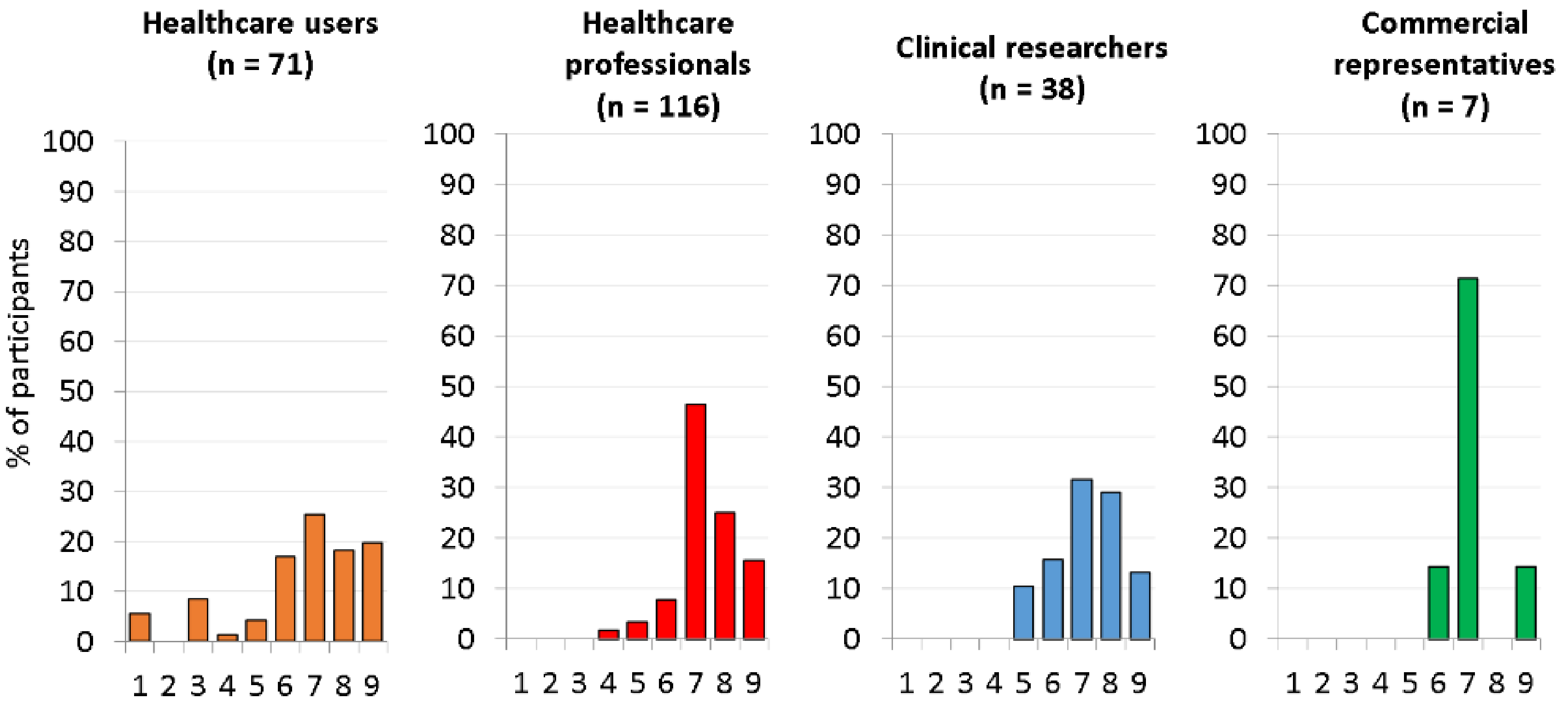

Domain Category: Health-related quality of life

Rating scale:

1	2	3	4	5	6	7	8	9
Not at all important			*Important but not critical*			*Critical*		

NIHR | National Institute for Health Research

14. IMPACT ON RELATIONSHIPS

CROSSSD

Effect of your hearing loss or your device on making new relationships and maintaining relationships with a spouse or partner; family; friends and colleagues

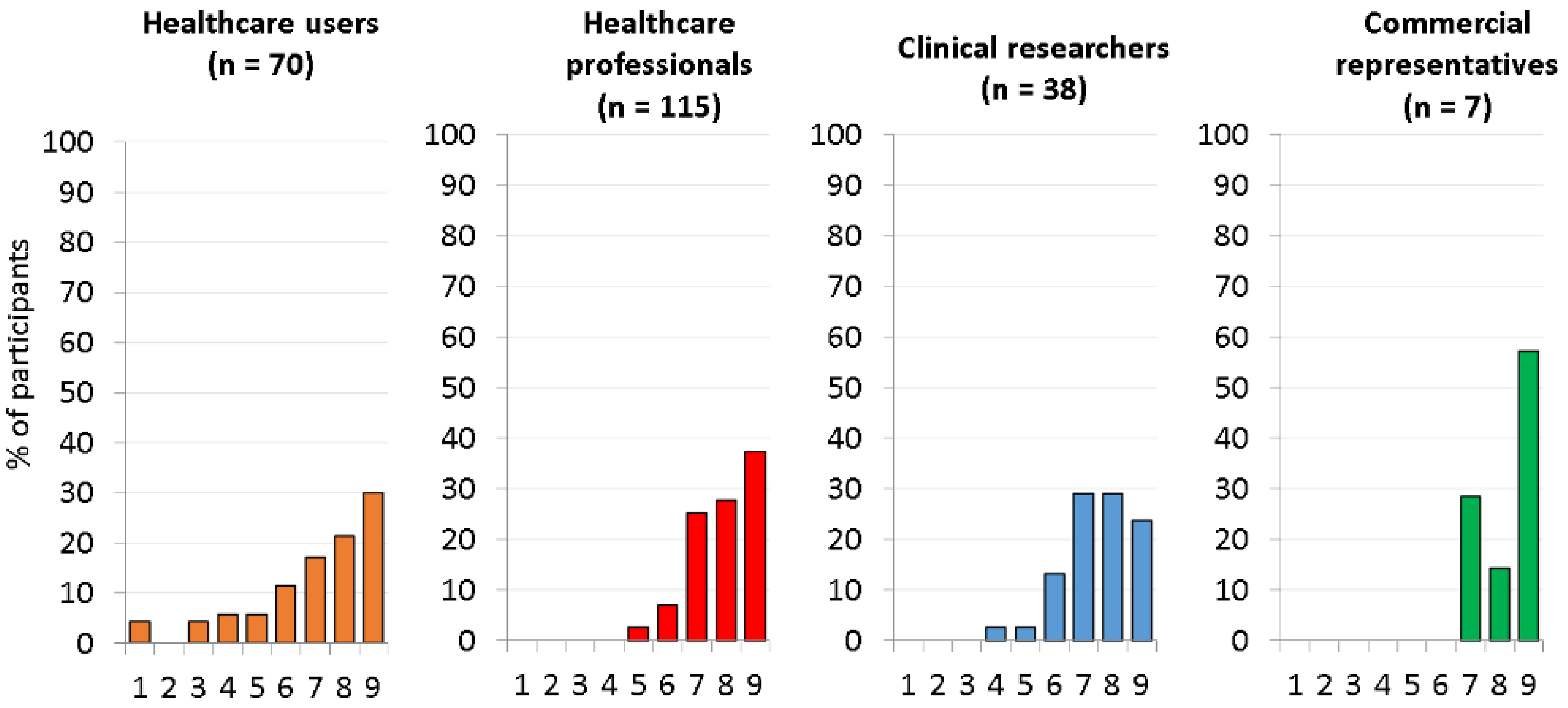

Domain Category: Health-related quality of life

NIHR | National Institute for Health Research

Rating scale:

1	2	3	4	5	6	7	8	9
Not at all important			*Important but not critical*			*Critical*		

15. IMPACT ON SOCIAL SITUATIONS

Your hearing loss or device limiting your ability to fully participate in the social world; especially in challenging situations or where a lot of effort is needed to follow the conversation (for example; at a restaurant; at the park; in a bar or at a party)

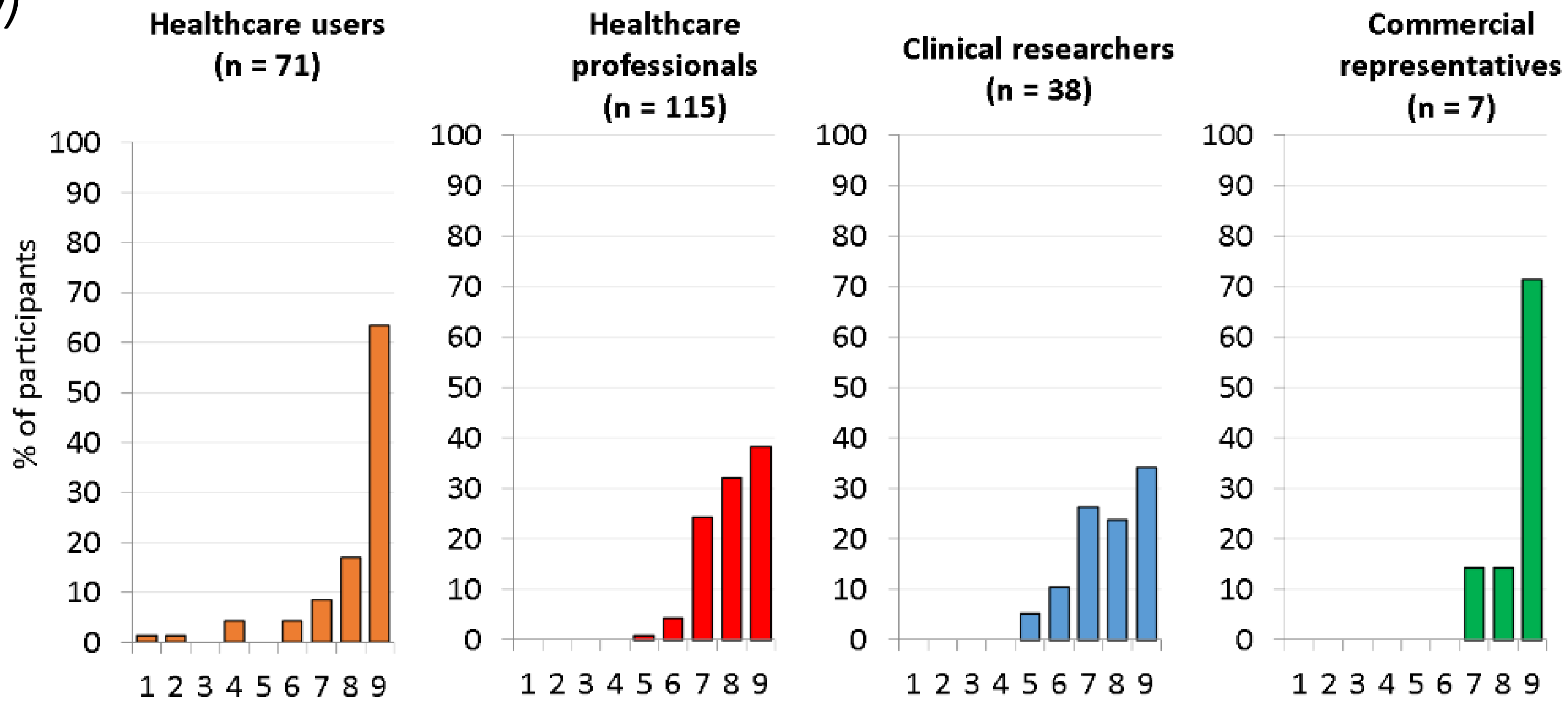

Domain Category: Health-related quality of life

Rating scale:

1	2	3	4	5	6	7	8	9
Not at all important			*Important but not critical*			*Critical*		

NIHR | National Institute for Health Research

16. IMPACT ON WORK

Effect of your hearing loss or device on your ability to carry out work tasks or job roles; or advancing your career

CROSSSD

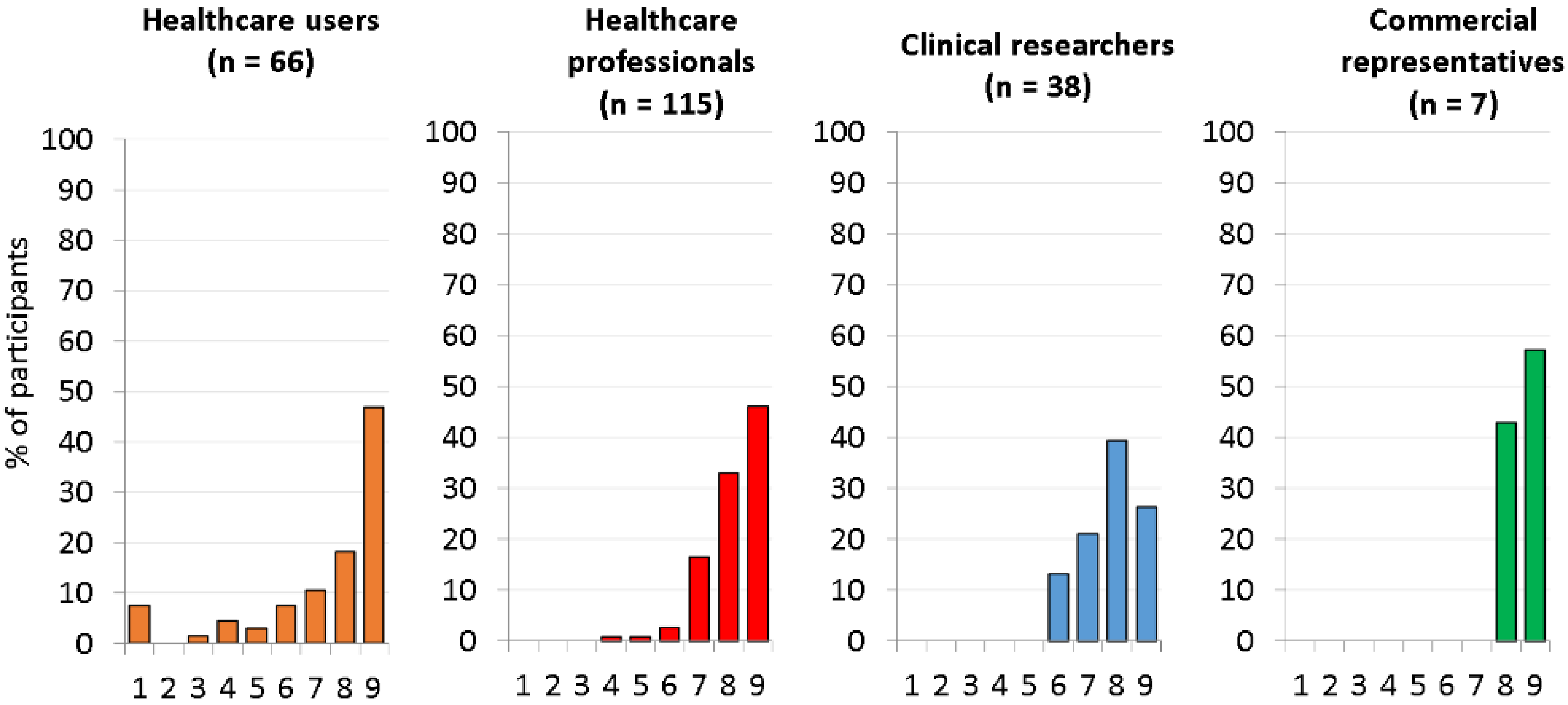

Domain Category: Health-related quality of life

NIHR | National Institute for Health Research

Rating scale:

1	2	3	4	5	6	7	8	9
Not at all important			*Important but not critical*			*Critical*		

17. BEING AWARE OF A SOUND

Being aware of a sound and recognising what that sound is (for example; being aware that someone has started to speak)

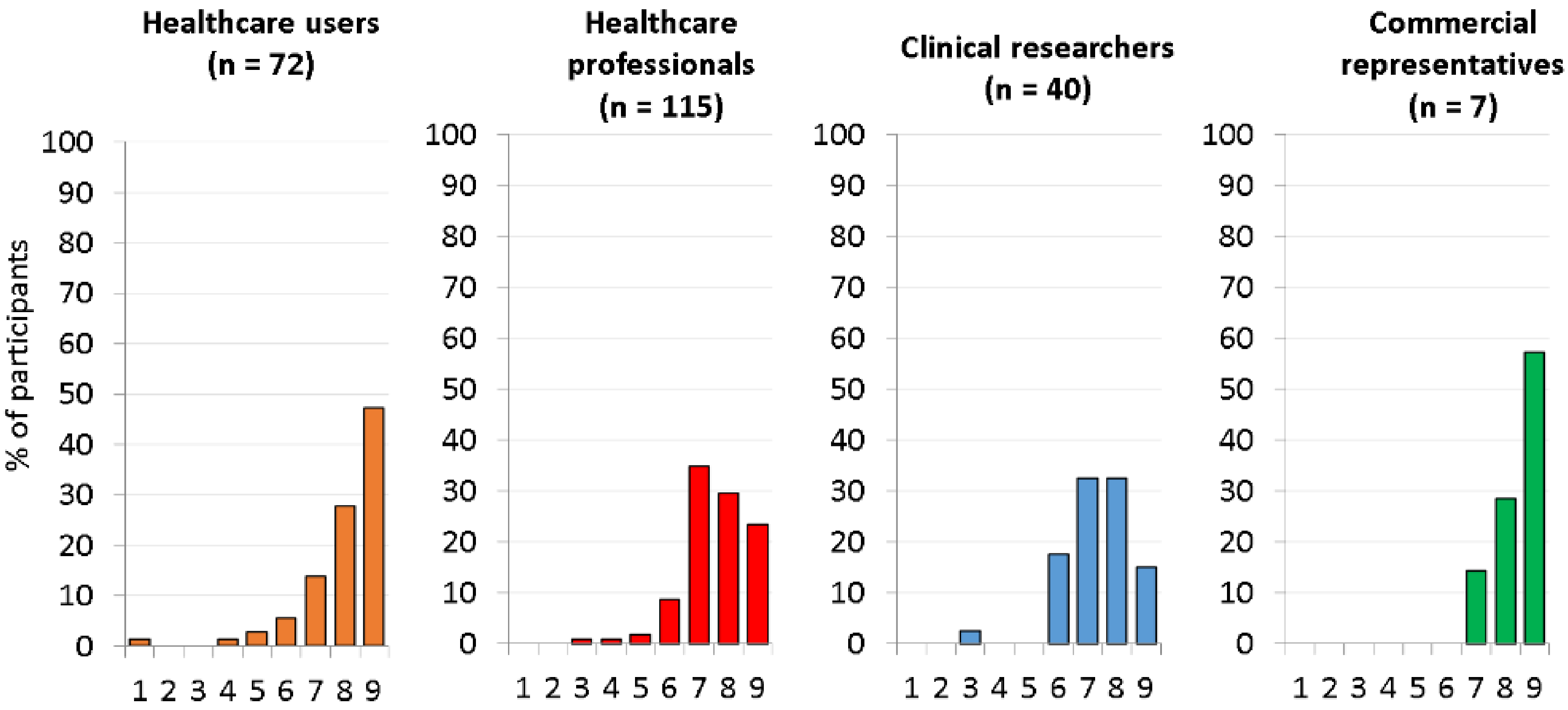

Domain Category: Hearing disability

Rating scale:

1	2	3	4	5	6	7	8	9
Not at all important			*Important but not critical*			*Critical*		

NIHR | National Institute for Health Research

18. LISTENING IN COMPLEX SITUATIONS

The difficulty experienced when listening to a sound while separating it out from a background of other sounds

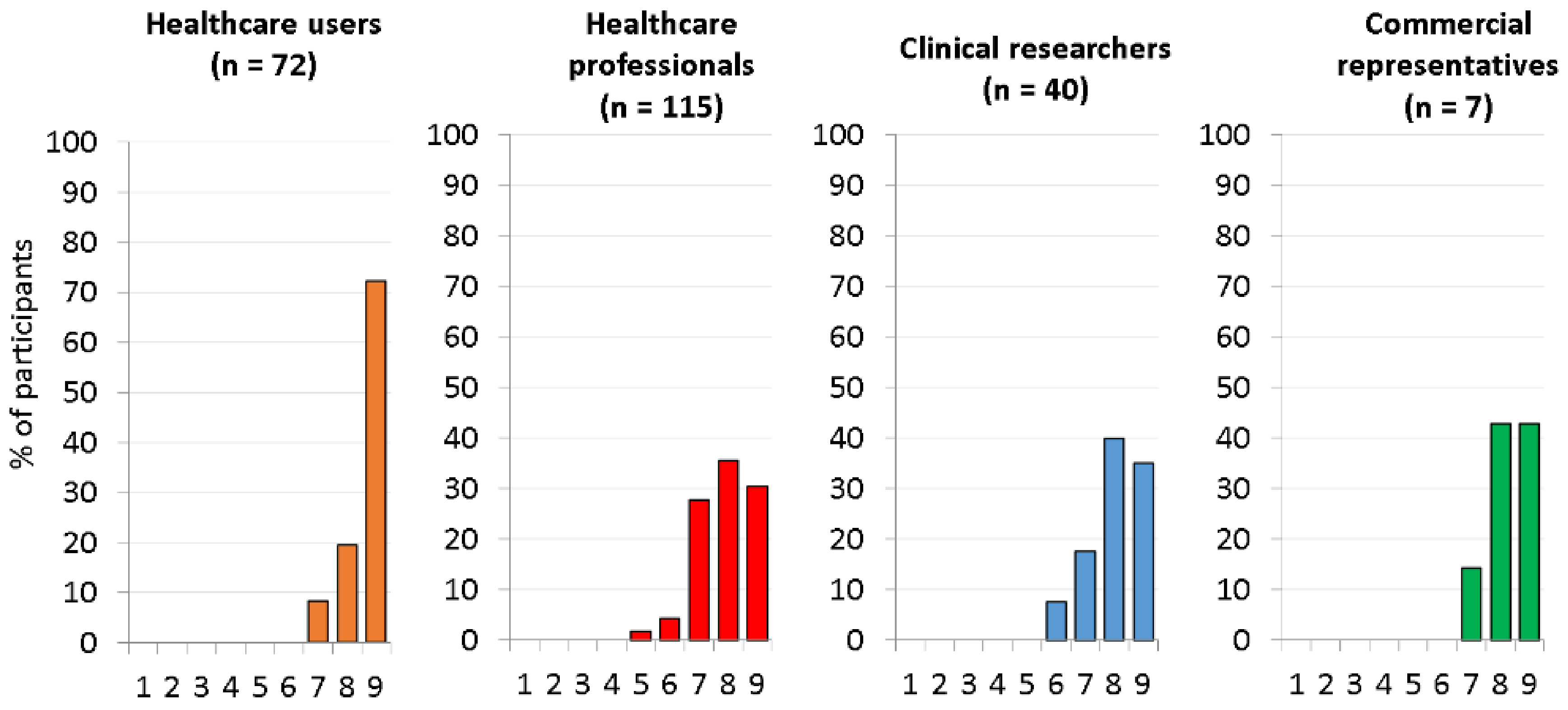

Domain Category:
Hearing disability

Rating scale:

1	2	3	4	5	6	7	8	9
Not at all important			*Important but not critical*			*Critical*		

NIHR | National Institute for Health Research

19. LISTENING IN REVERBERANT CONDITIONS

The difficulty experienced when listening in places where the sound reflects off the walls; floor or ceiling (echoes); creating a blurred sound. For example; understanding announcements in train stations or airports

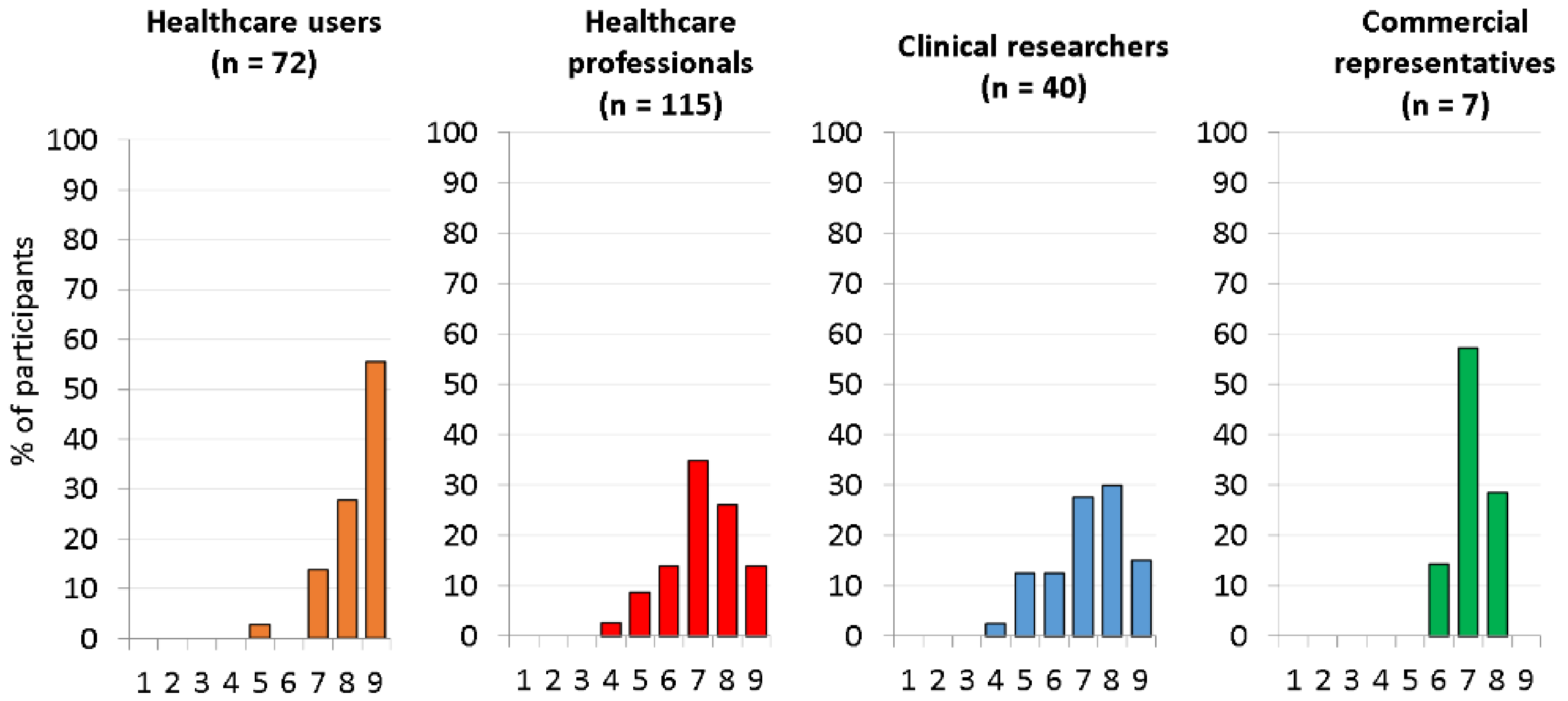

Domain Category:
Hearing disability

Rating scale:

1	2	3	4	5	6	7	8	9
Not at all important			*Important but not critical*			*Critical*		

NIHR National Institute for Health Research

20. ONE-TO-ONE CONVERSATION IN QUIET

Listening and understanding one person; in a quiet environment

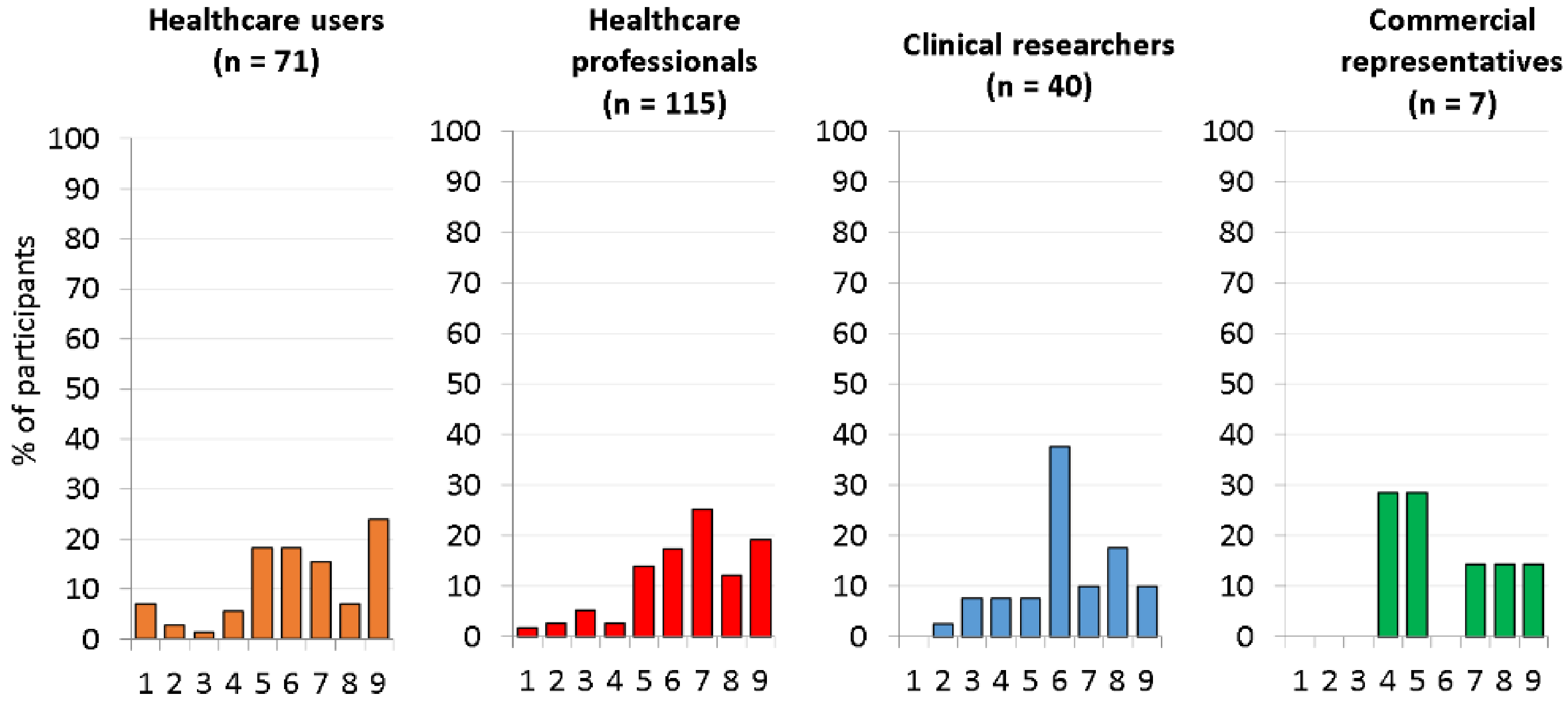

Domain Category:
Hearing disability

NIHR | National Institute for Health Research

Rating scale:

1	2	3	4	5	6	7	8	9
Not at all important			*Important but not critical*			*Critical*		

21. GROUP CONVERSATION IN QUIET

Listening and following a conversation between a group of people; in a quiet environment

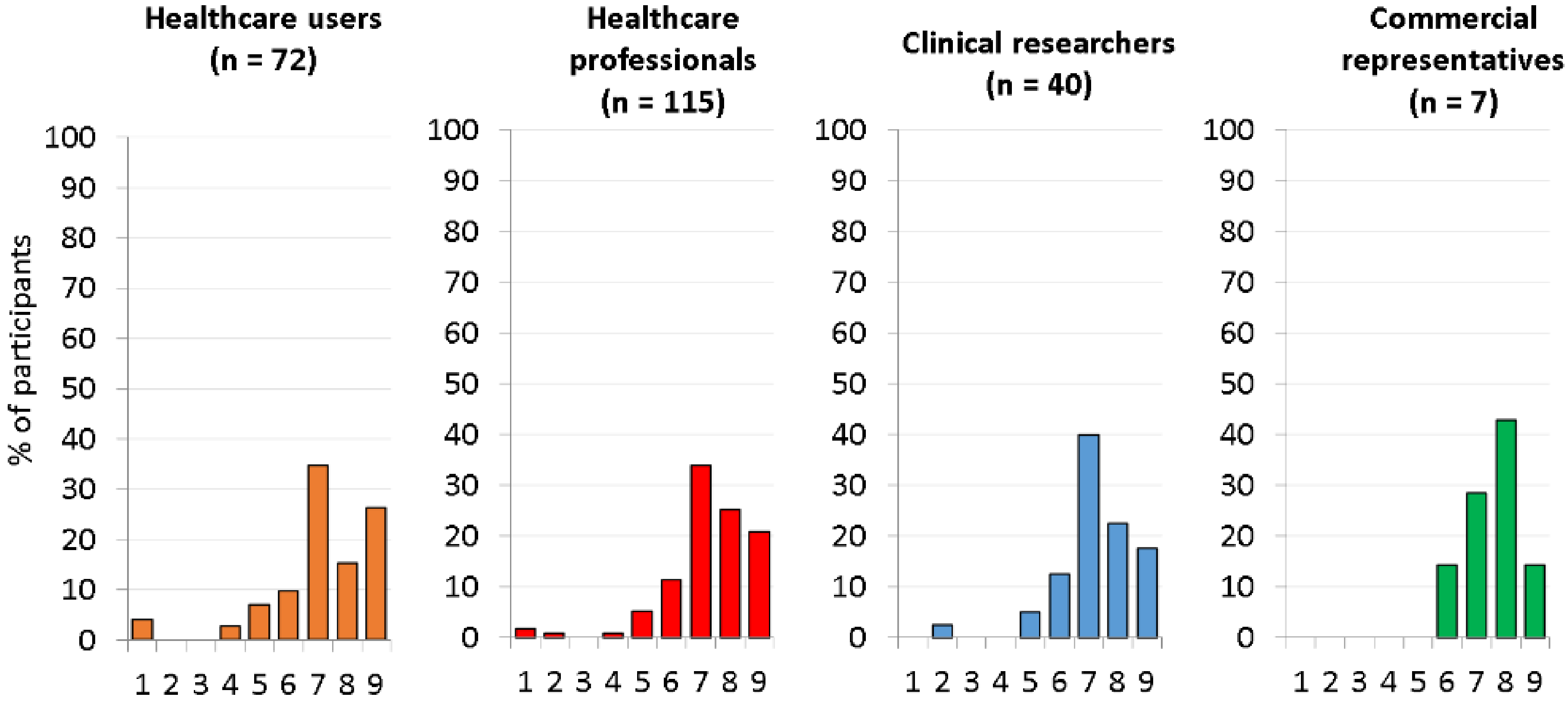

Domain Category:

Hearing disability

NIHR | National Institute for Health Research

Rating scale:

1	2	3	4	5	6	7	8	9
Not at all important			*Important but not critical*			*Critical*		

22. ONE-TO-ONE CONVERSATION IN GENERAL NOISE

Listening and understanding one person; in a noisy environment

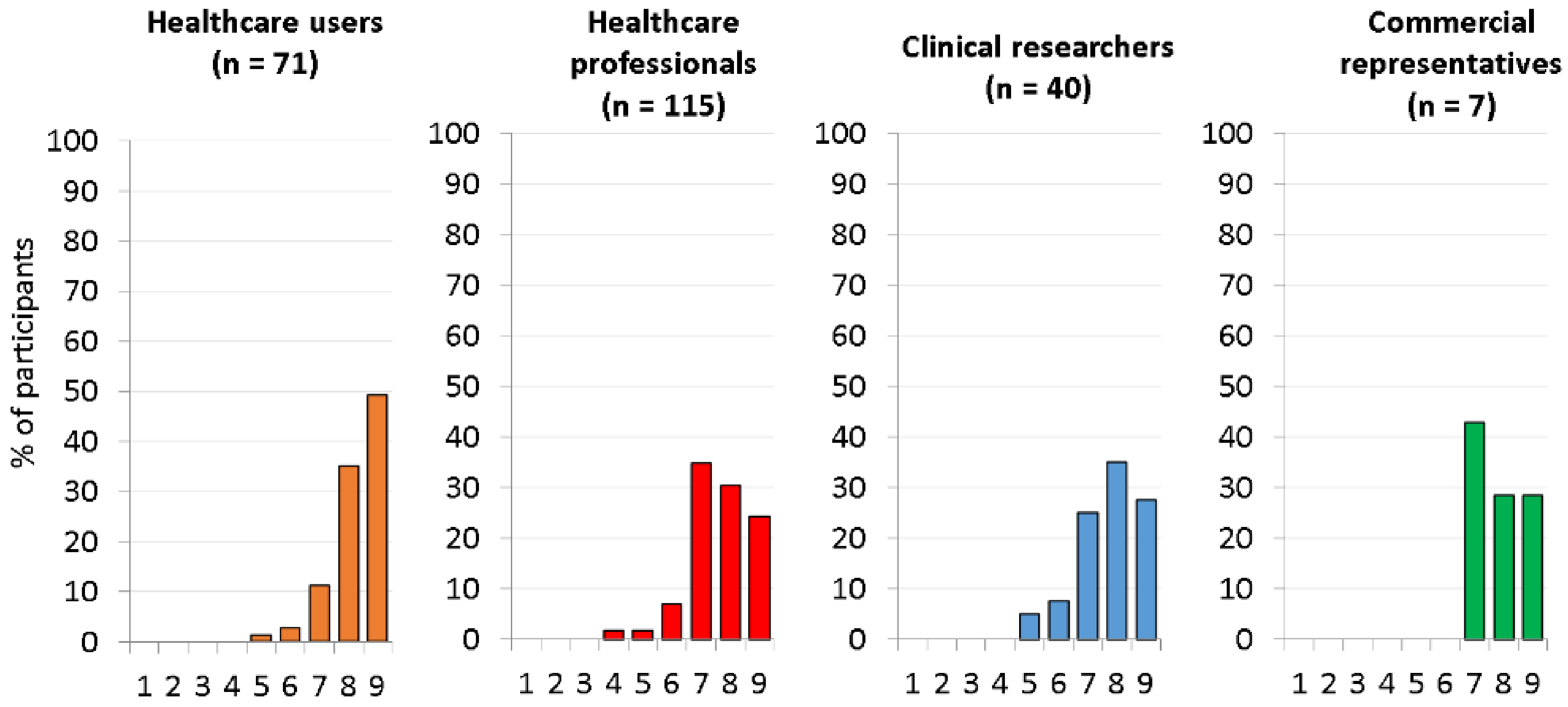

Domain Category:
Hearing disability

NIHR | National Institute for Health Research

Rating scale:

1	2	3	4	5	6	7	8	9
Not at all important			*Important but not critical*			*Critical*		

23. GROUP CONVERSATION IN NOISY SOCIAL SITUATIONS

Listening and following a conversation between a group of people; when others are talking in the background

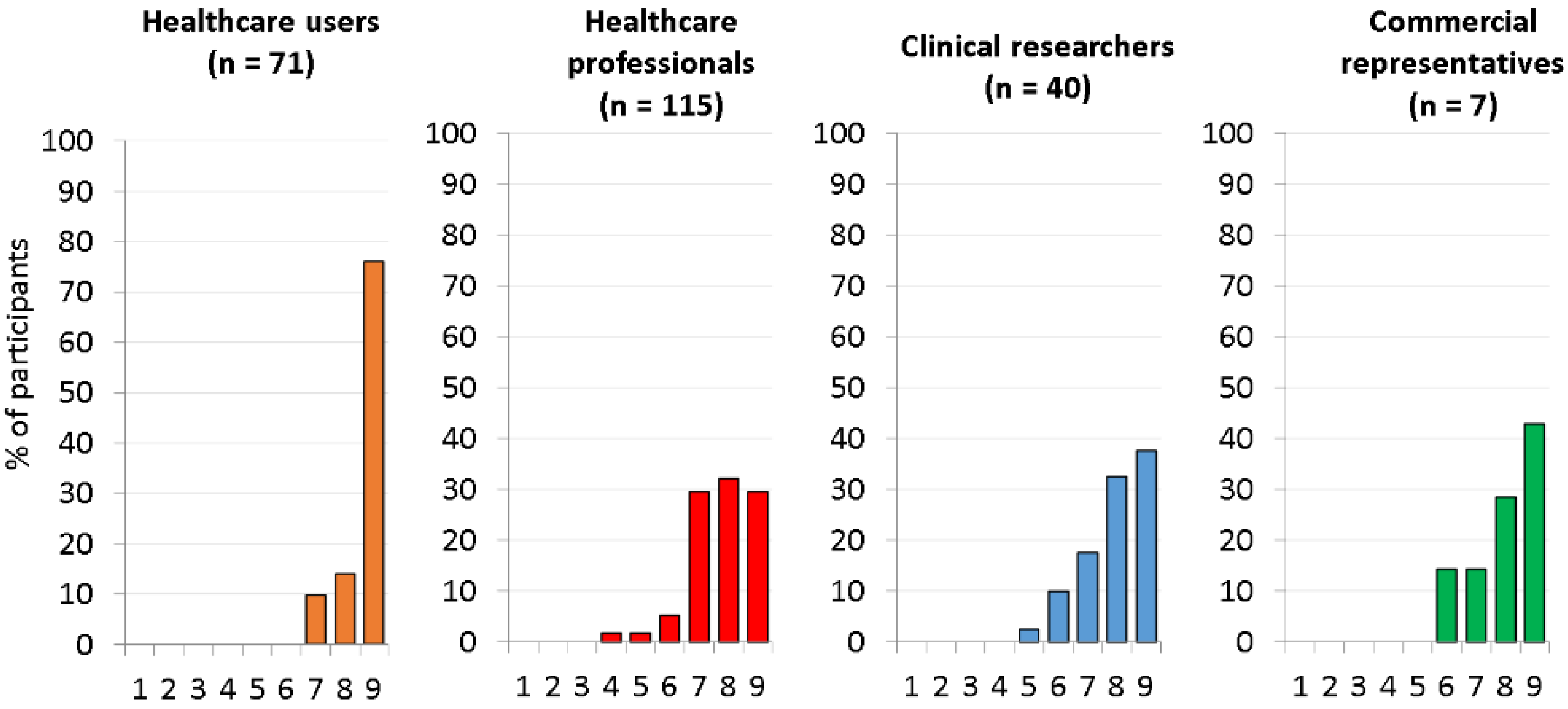

Domain Category:
Hearing disability

NIHR | National Institute for Health Research

Rating scale:

1	2	3	4	5	6	7	8	9
Not at all important			*Important but not critical*			*Critical*		

24. SOUND LOCALISATION

Knowing where a sound is coming from

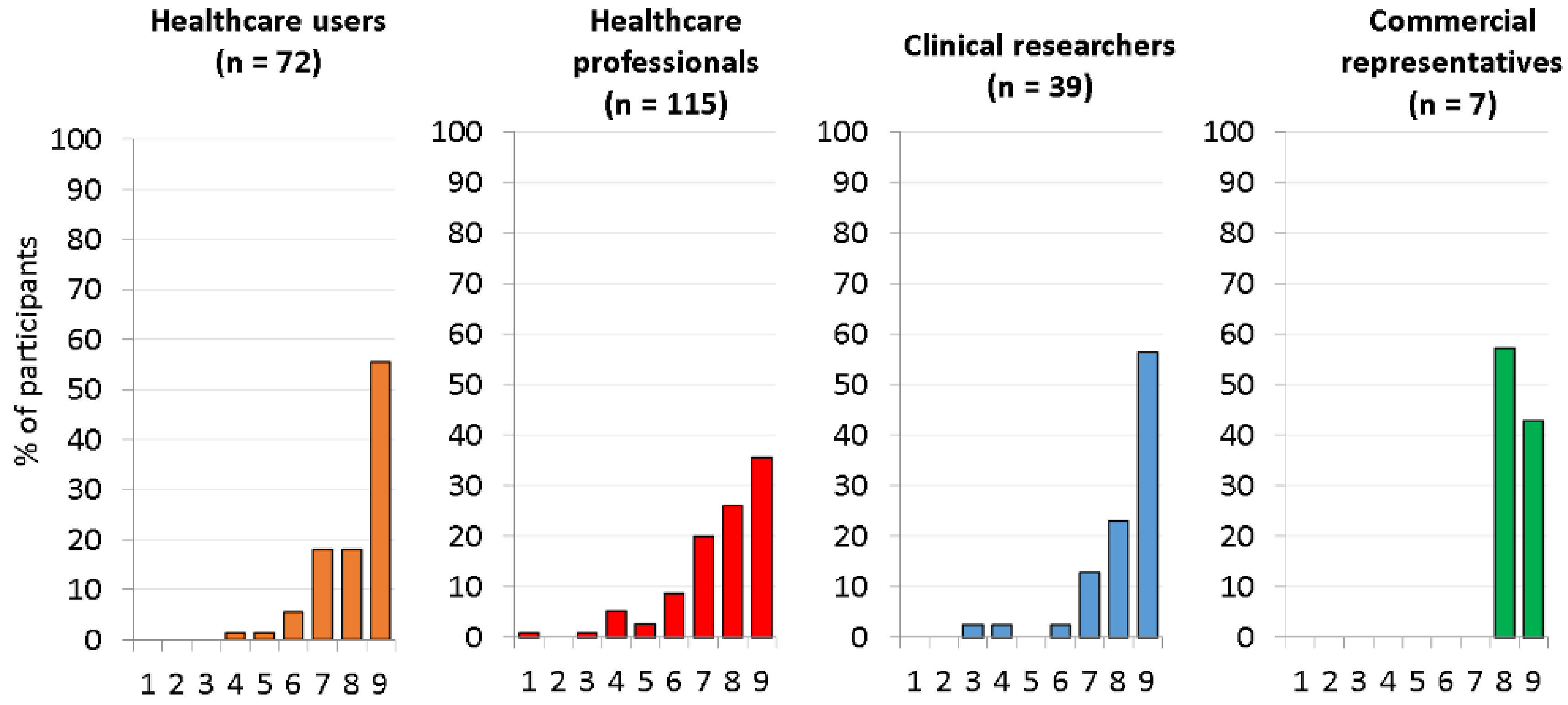

Domain Category:

Spatial hearing

National Institute for Health Research

Rating scale:

1	2	3	4	5	6	7	8	9
Not at all important			*Important but not critical*			*Critical*		

25. SOUND DISTANCE

Knowing if a sound is close by or far away

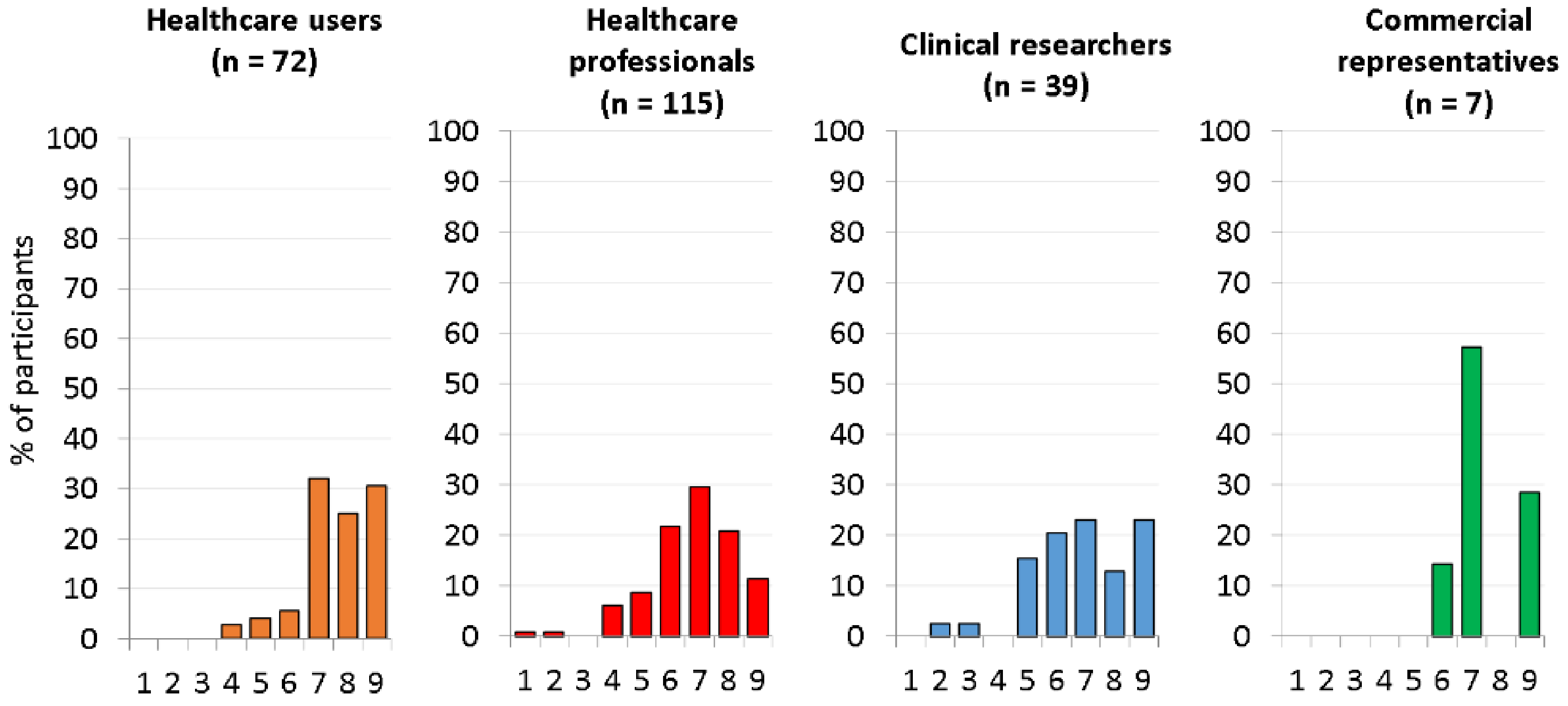

Domain Category:

Spatial hearing

NIHR National Institute for Health Research

Rating scale:

1	2	3	4	5	6	7	8	9
Not at all important			*Important but not critical*			*Critical*		

26. SPATIAL ORIENTATION

Knowing where you are in relation to the position of a sound source

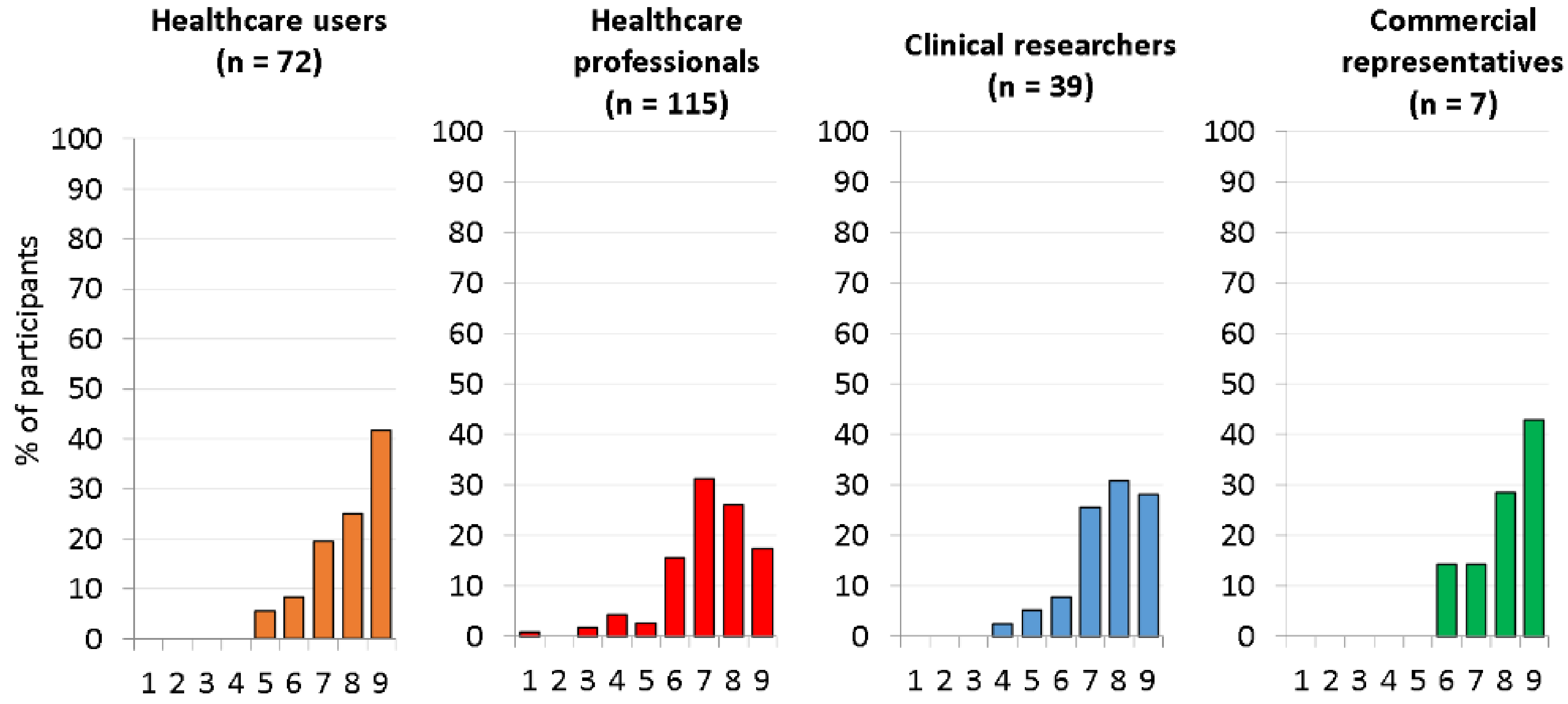

Domain Category:

Spatial hearing

National Institute for Health Research

Rating scale:

1	2	3	4	5	6	7	8	9
Not at all important			*Important but not critical*			*Critical*		

27. ENJOYMENT OF LISTENING TO MUSIC

Appreciating 'stereo'; '3-dimensional' or 'surround sound' quality of live or recorded music

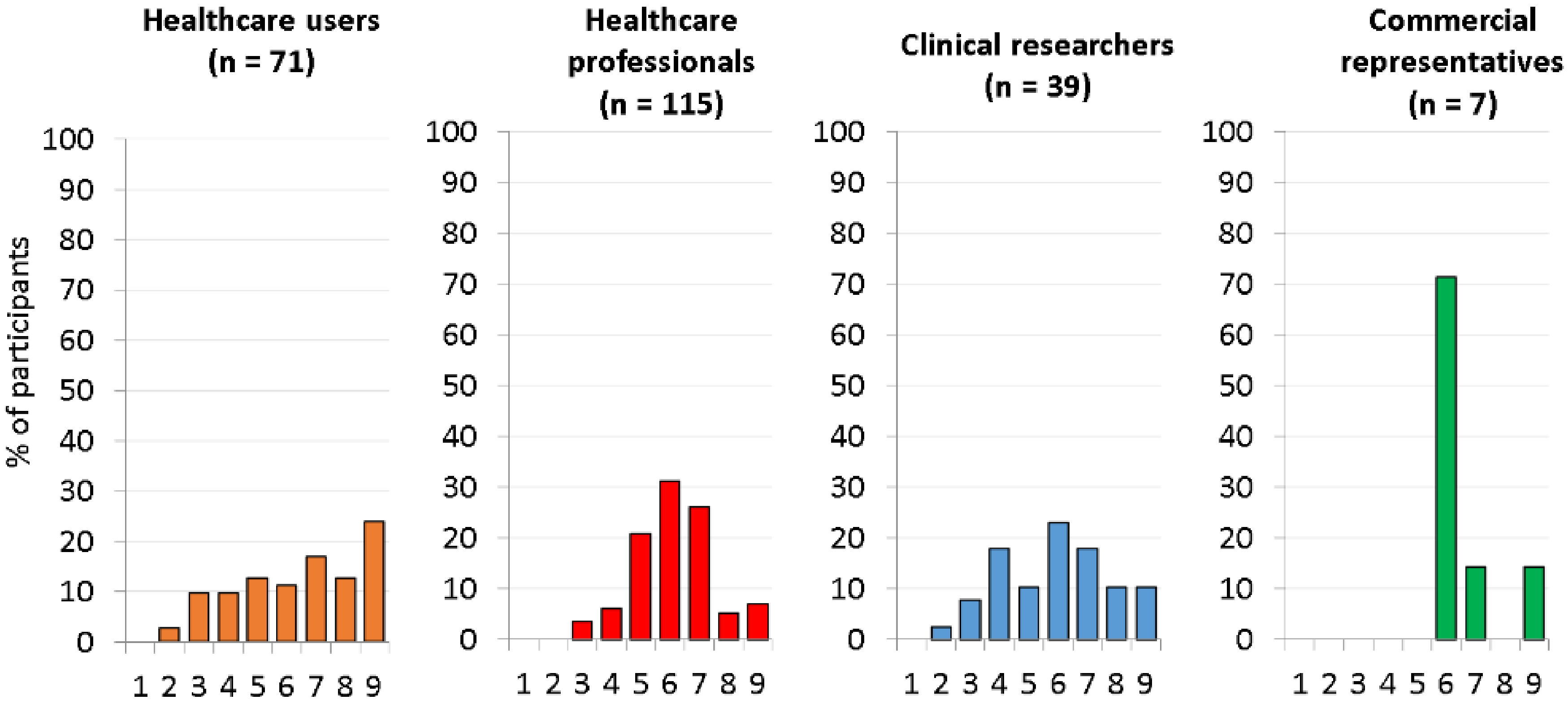

Domain Category:

Spatial hearing

Rating scale:

1	2	3	4	5	6	7	8	9
Not at all important			*Important but not critical*			*Critical*		

NIHR | National Institute for Health Research

28. PHYSICAL TIREDNESS

Tiredness or fatigue from the effort of listening or when you need to turn your head repeatedly to listen in social situations

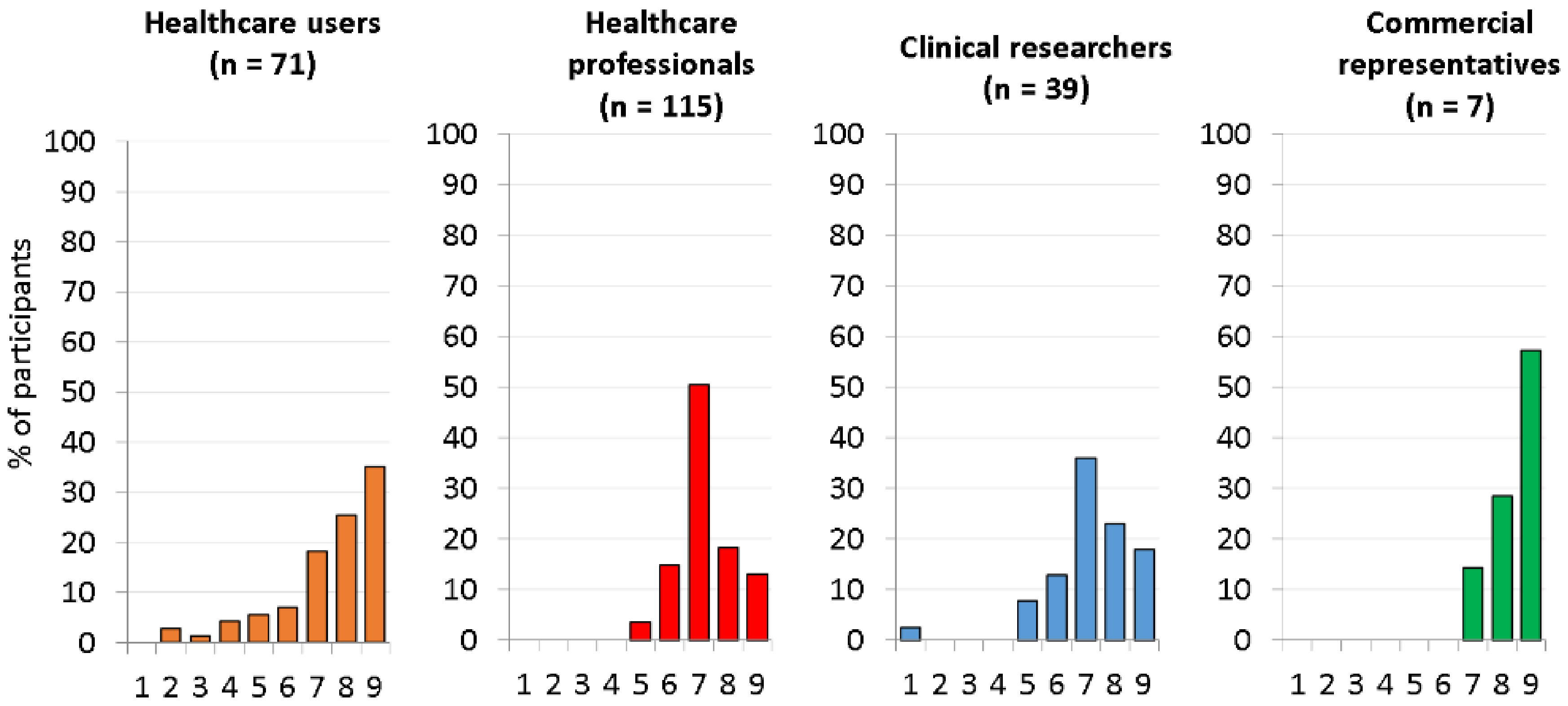

Domain Category:
Physical effects

NIHR | National Institute for Health Research

Rating scale:

1	2	3	4	5	6	7	8	9
Not at all important			*Important but not critical*			*Critical*		

29. BALANCE PROBLEMS

Feeling unbalanced and the effect it has on your ability to walk or move normally

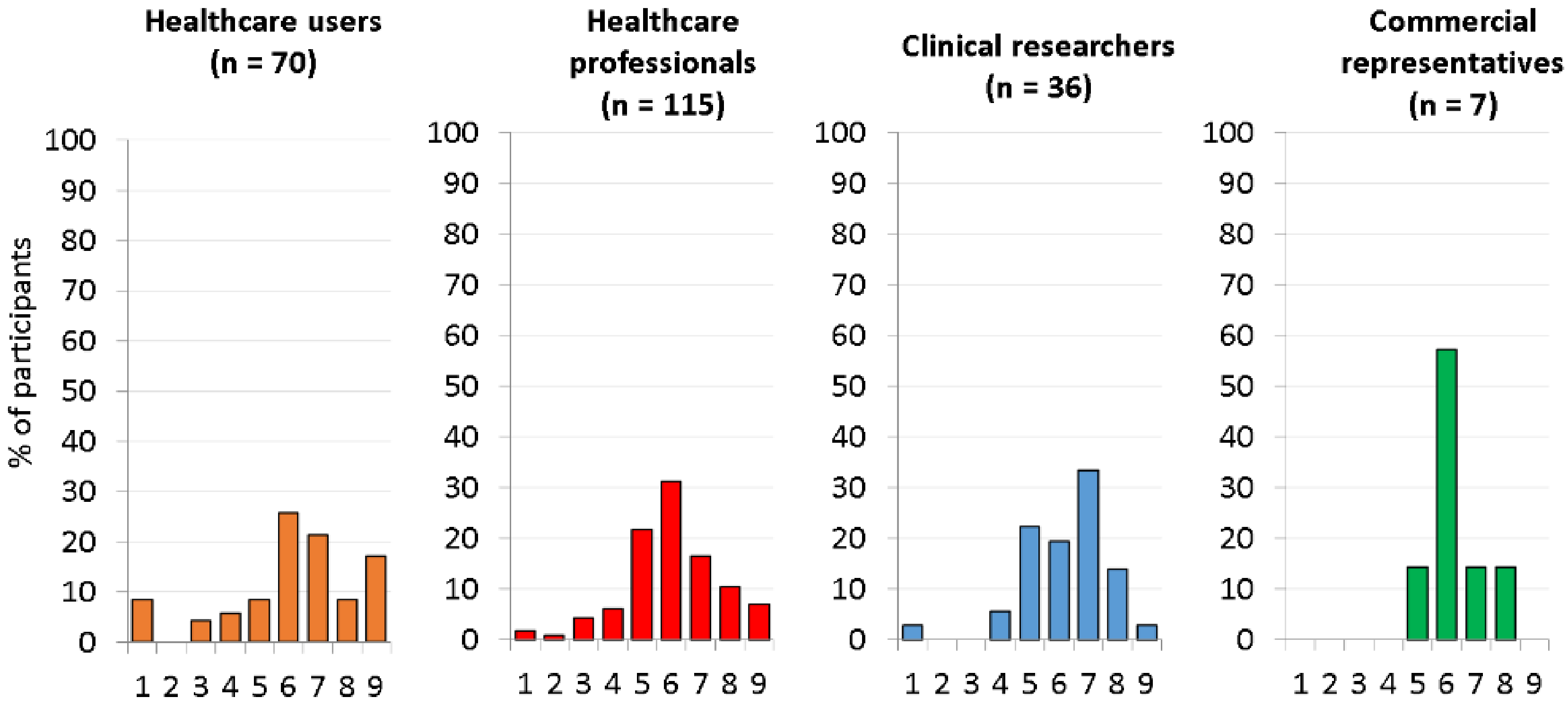

Domain Category:
Physical effects

National Institute for Health Research

Rating scale:

1	2	3	4	5	6	7	8	9
Not at all important			*Important but not critical*			*Critical*		

30. MANUAL DEXTERITY

Having the fine motor skills needed to use your device effectively (for example; putting the device on; changing the batteries)

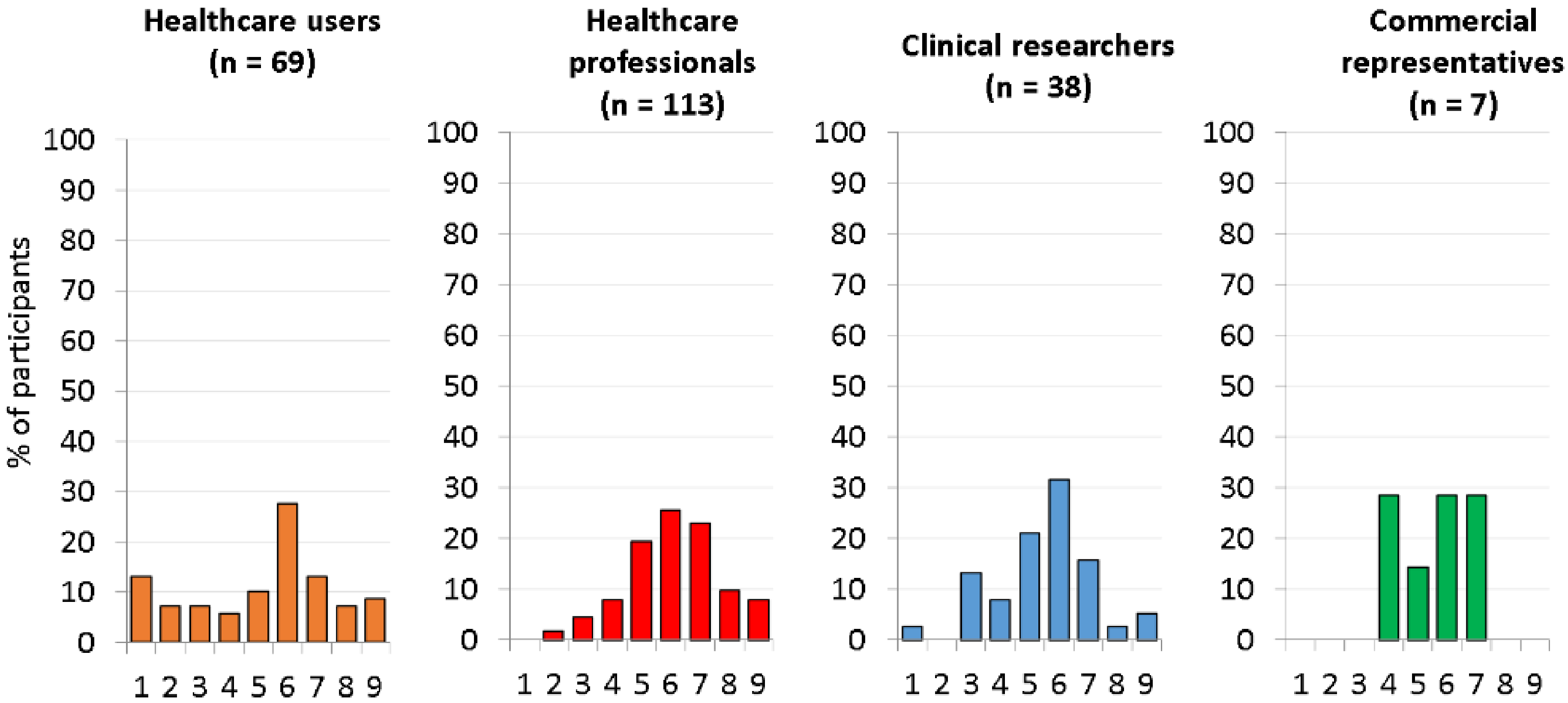

Domain Category:
Physical effects

NIHR | National Institute for Health Research

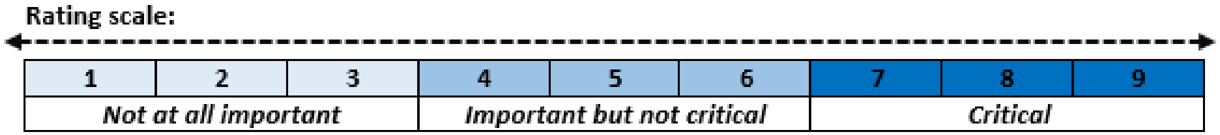

31. TINNITUS-RELATED BRAIN CHANGES

Changes in brain structure or function associated with tinnitus

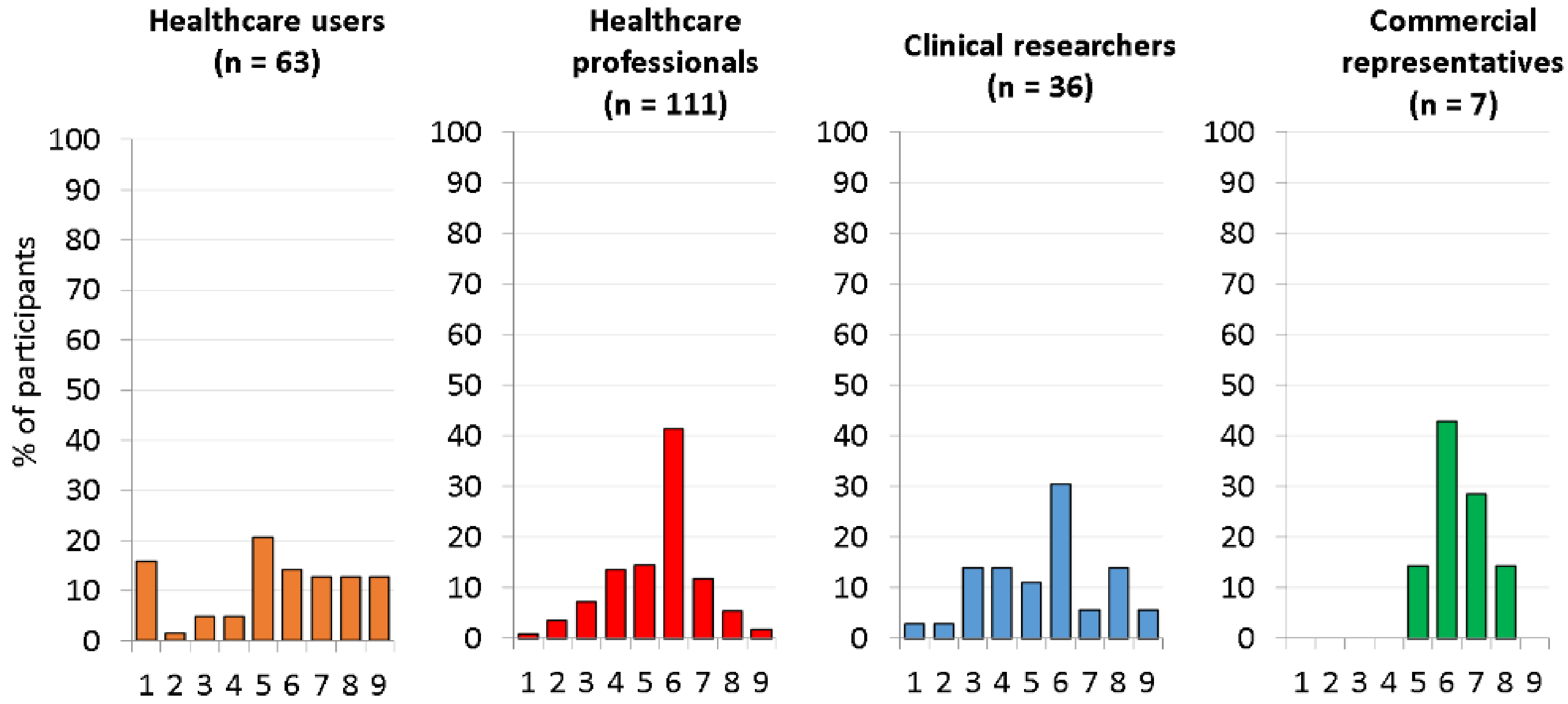

Domain Category:
Physical effects

NIHR | National Institute for Health Research

Rating scale:

1	2	3	4	5	6	7	8	9
Not at all important			*Important but not critical*			*Critical*		

32. HEARING-RELATED BRAIN CHANGES

Changes in brain structure or function associated with hearing loss

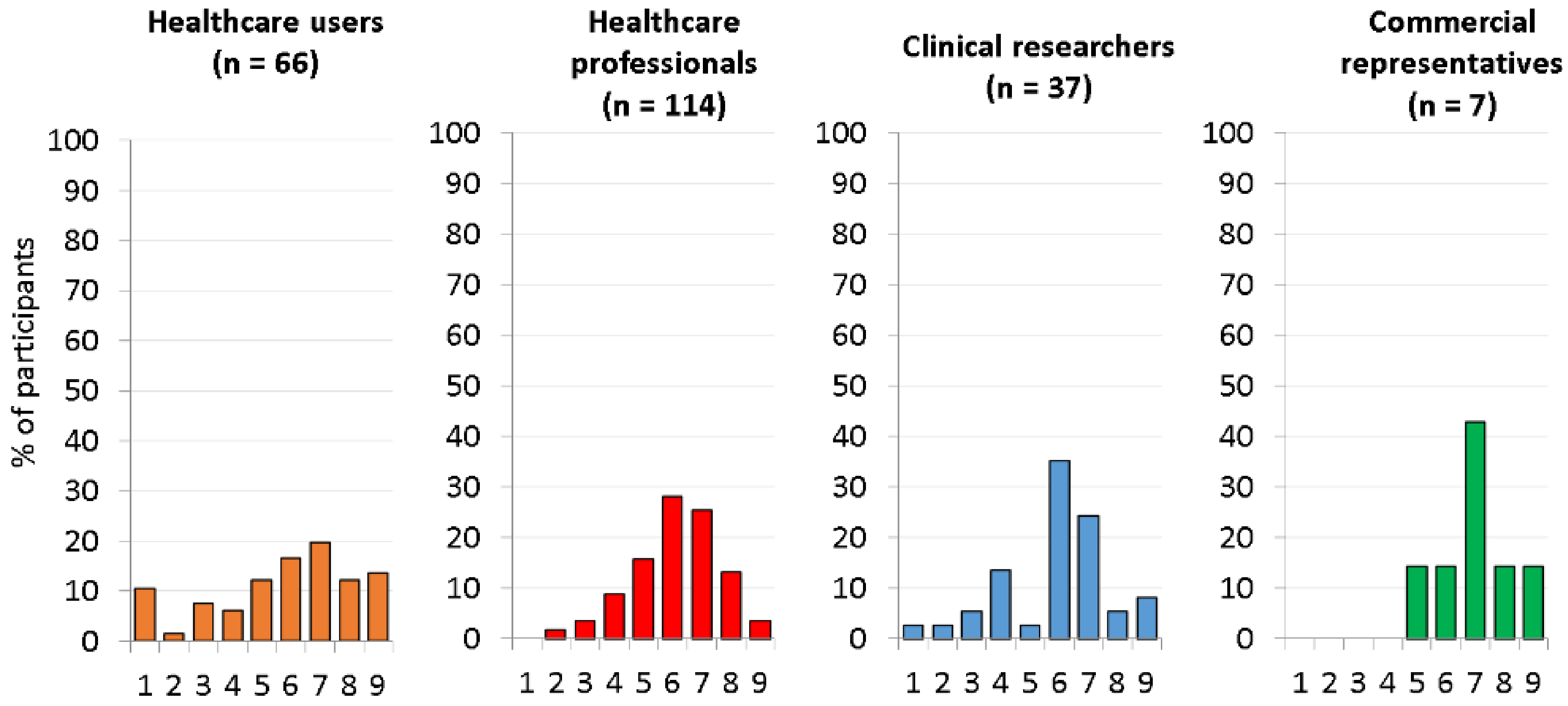

Domain Category:
Physical effects

NIHR | National Institute for Health Research

Rating scale:

1	2	3	4	5	6	7	8	9
Not at all important			*Important but not critical*			*Critical*		

33. SELF-STIGMA

Negative perception of yourself due to your hearing loss and feeling stigmatised for using a hearing aid

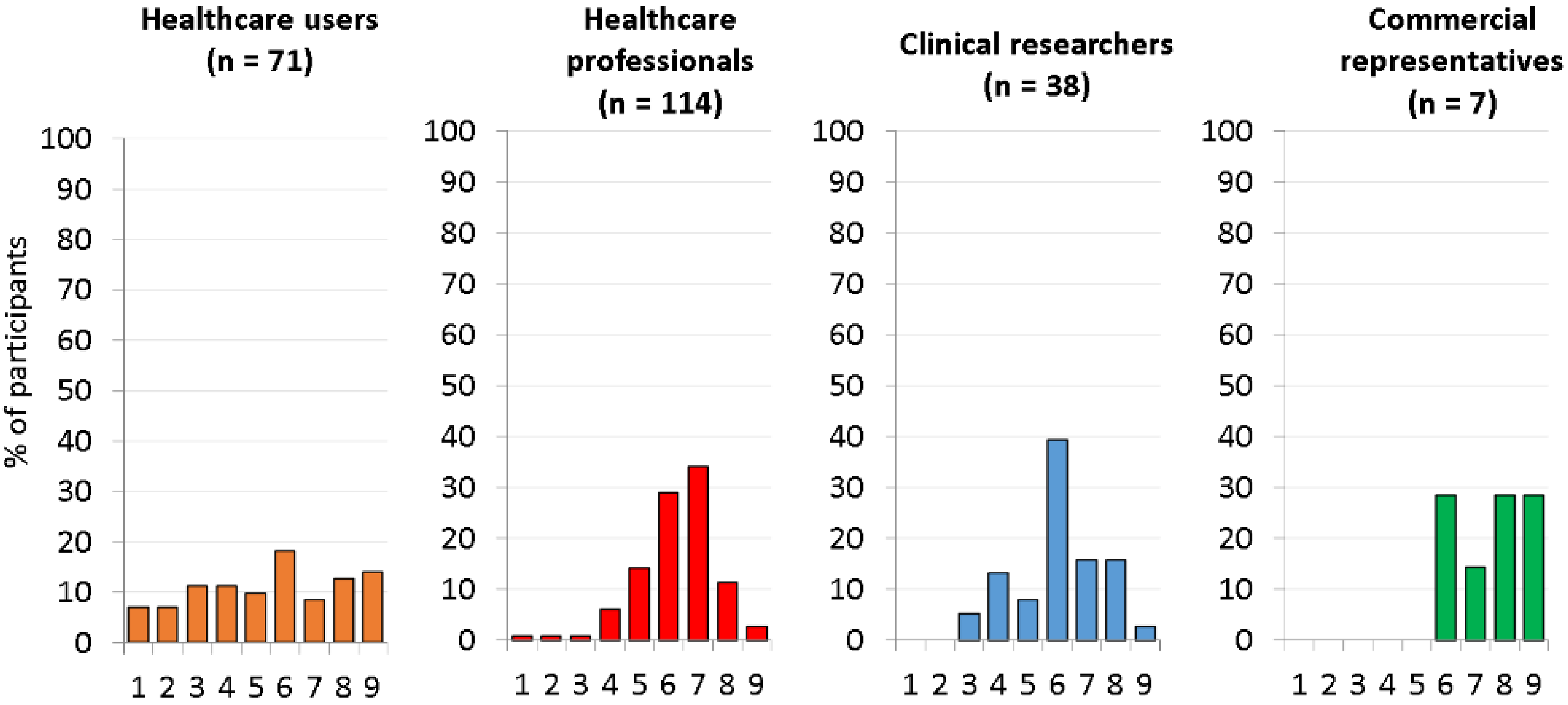

Domain Category: Self

Rating scale:

1	2	3	4	5	6	7	8	9
Not at all important			*Important but not critical*			*Critical*		

NIHR | National Institute for Health Research

34. SELF-IMAGE

Feeling incomplete or incapable because you are unable to do all the things that you want to do

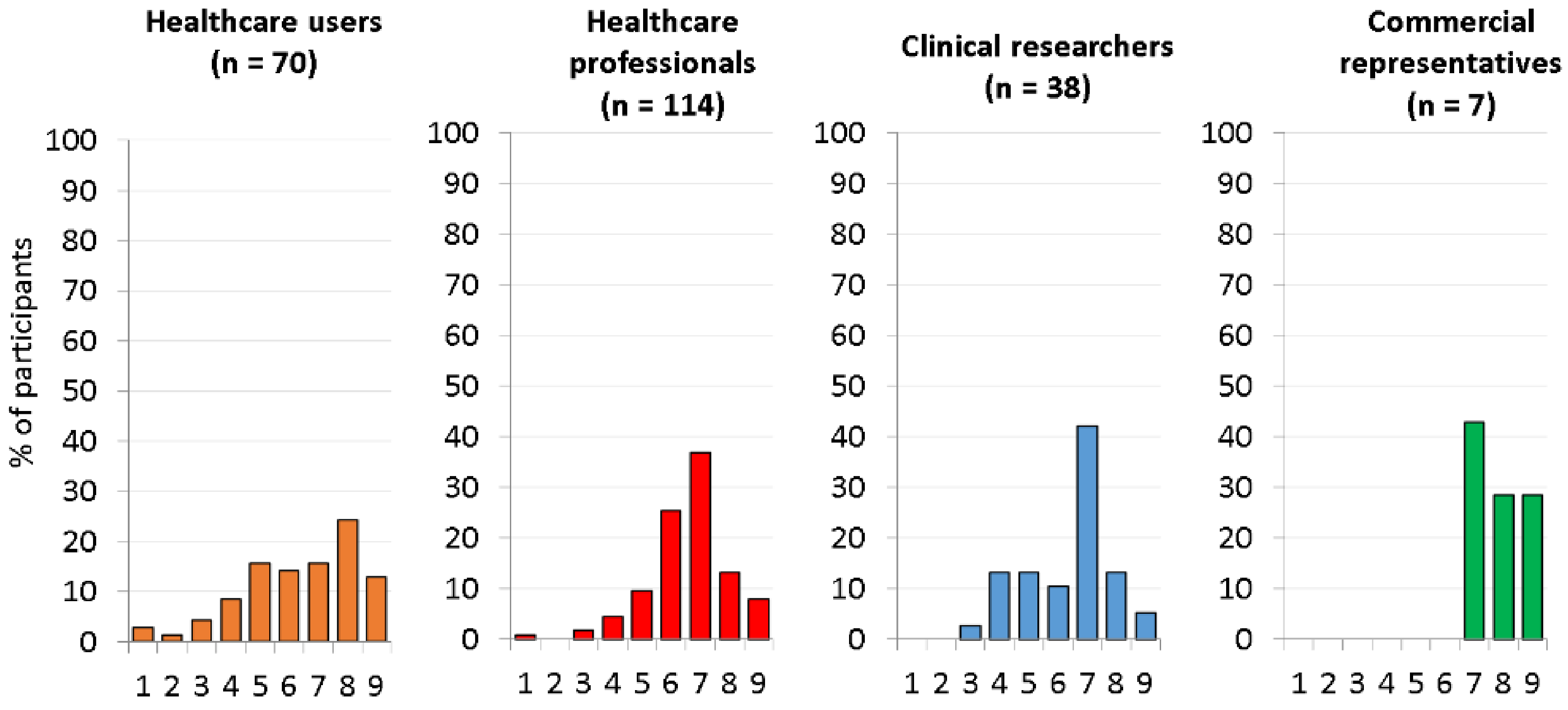

Domain Category: Self

NIHR | National Institute for Health Research

Rating scale:

1	2	3	4	5	6	7	8	9
Not at all important			*Important but not critical*			*Critical*		

35. PERSONAL SAFETY

How your hearing loss effects your awareness of potential hazards and threats in your daily life (for example; moving traffic; hazards at the workplace) and those you may not be able to see or hear (for example; other people behind you)

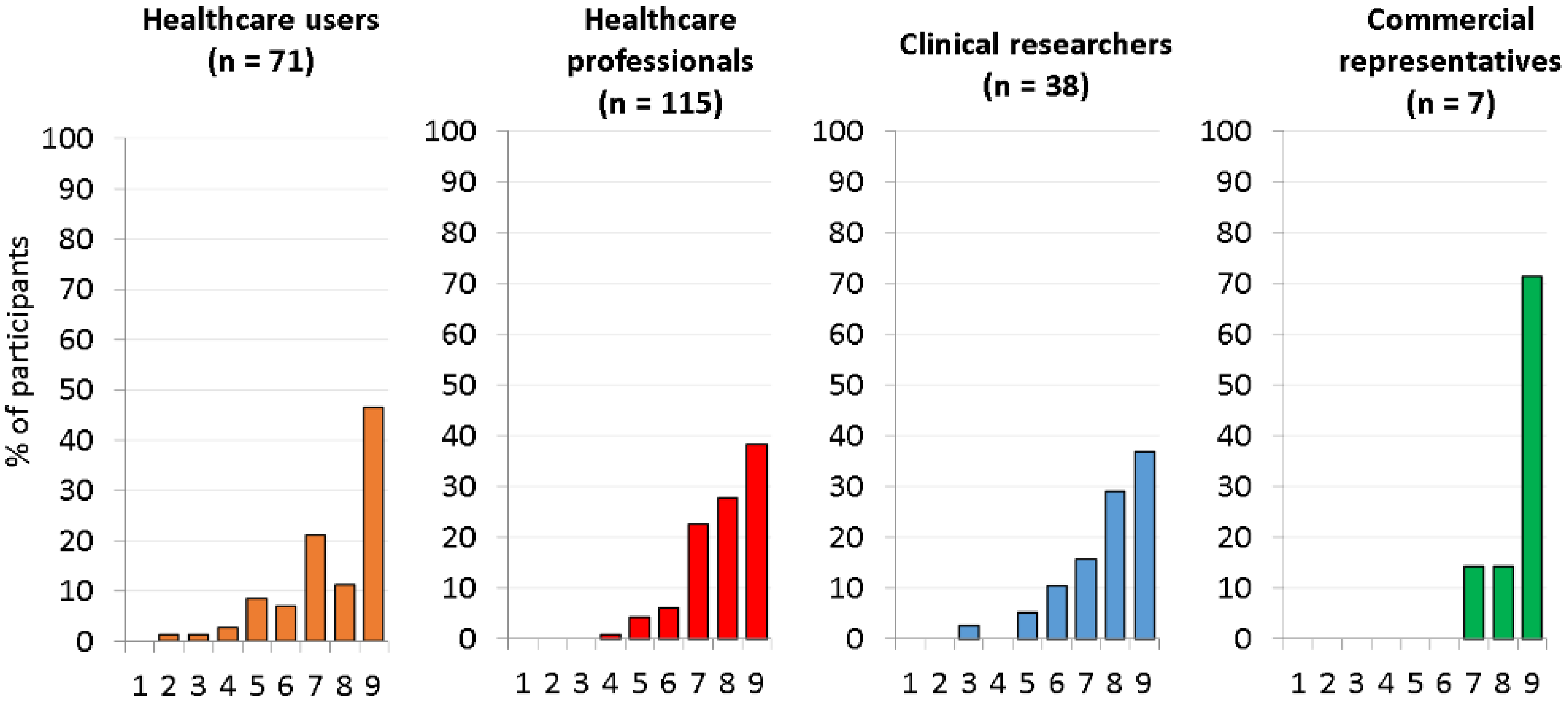

Domain Category: Self

Rating scale:

1	2	3	4	5	6	7	8	9
Not at all important			*Important but not critical*			*Critical*		

NIHR | National Institute for Health Research

36. PROTECTING YOUR HEARING

Making a conscious decision to avoid loud sounds or other risks to your hearing; or taking steps to protect your hearing

CROSSSD

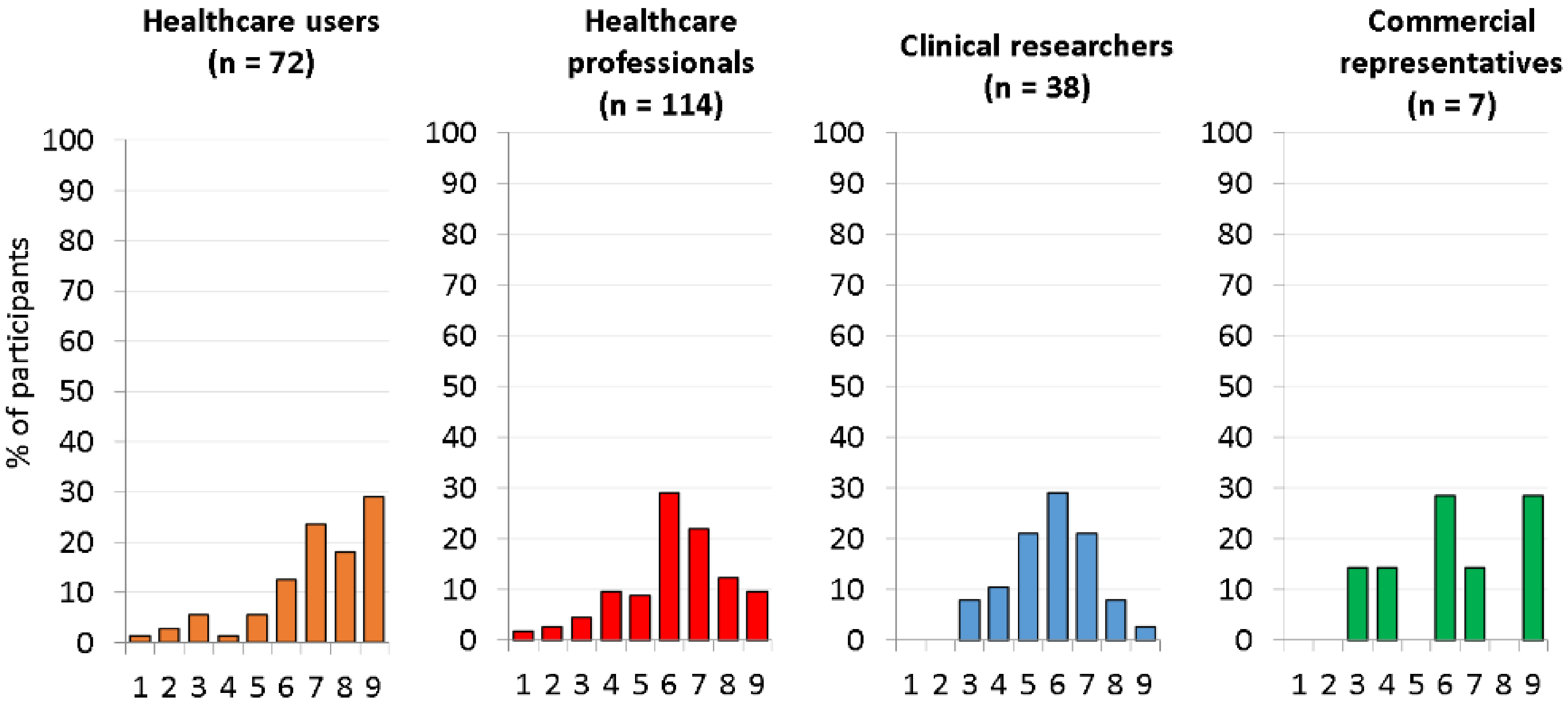

Domain Category: Self

Rating scale:

1	2	3	4	5	6	7	8	9
Not at all important			*Important but not critical*			*Critical*		

NIHR | National Institute for Health Research

37. LOUDNESS

How 'loud' a sound seems to you

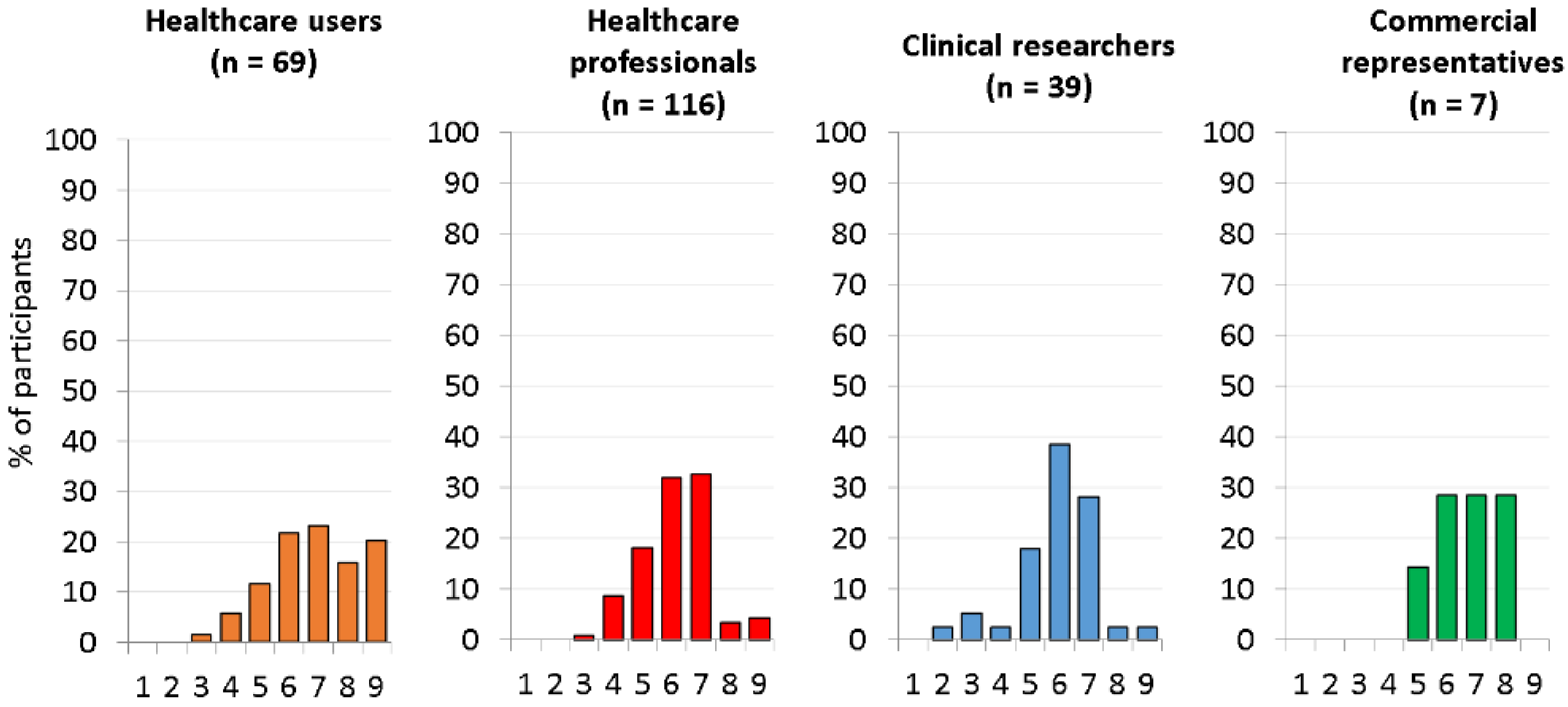

Domain Category:
Sound quality

Rating scale:

1	2	3	4	5	6	7	8	9
Not at all important			*Important but not critical*			*Critical*		

NIHR | National Institute for Health Research

38. FULLNESS

How 'full' a sound seems to you. This can also be described as the 'richness'; 'warmth' or 'depth' of a sound

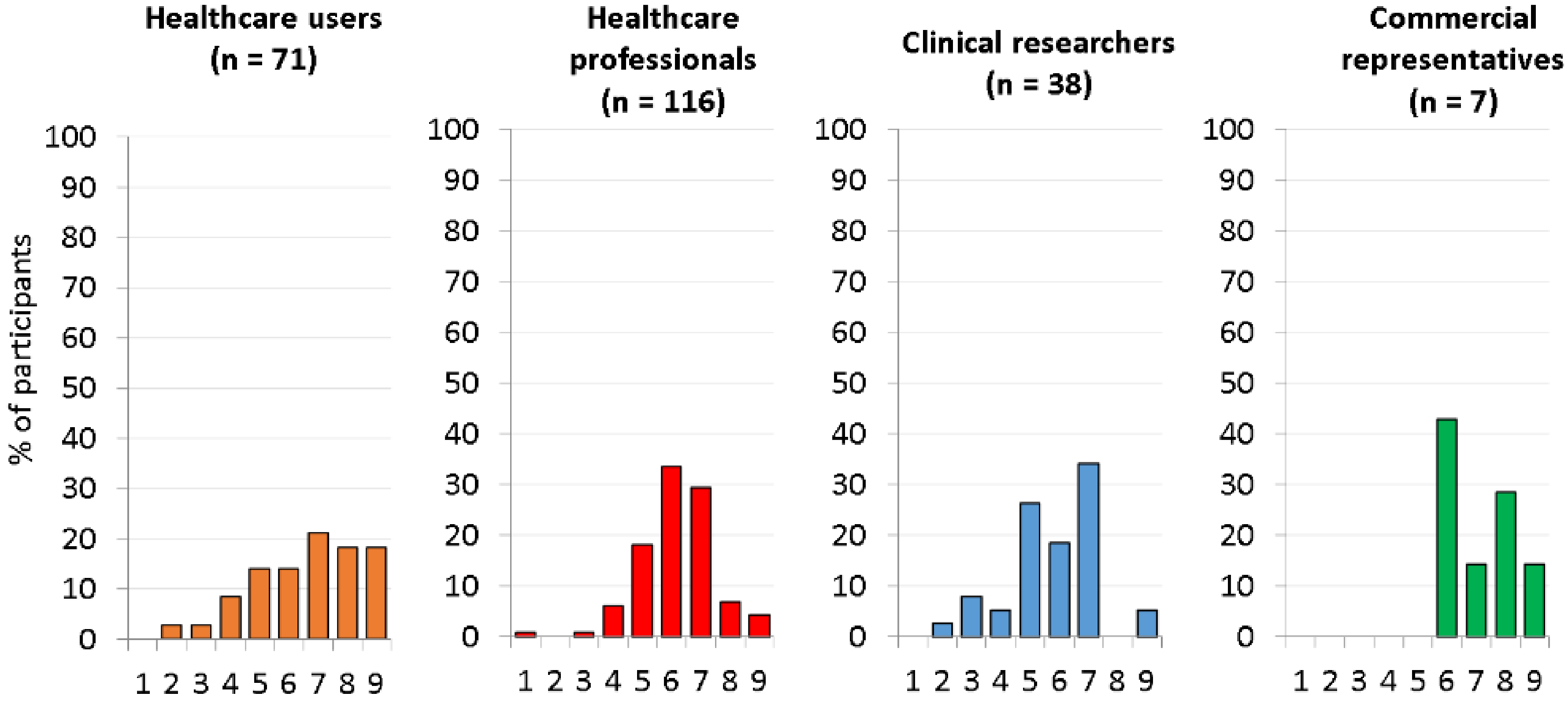

Domain Category:
Sound quality

Rating scale:

1	2	3	4	5	6	7	8	9
Not at all important			*Important but not critical*			*Critical*		

39. CLARITY

How 'clear' a sound seems to you

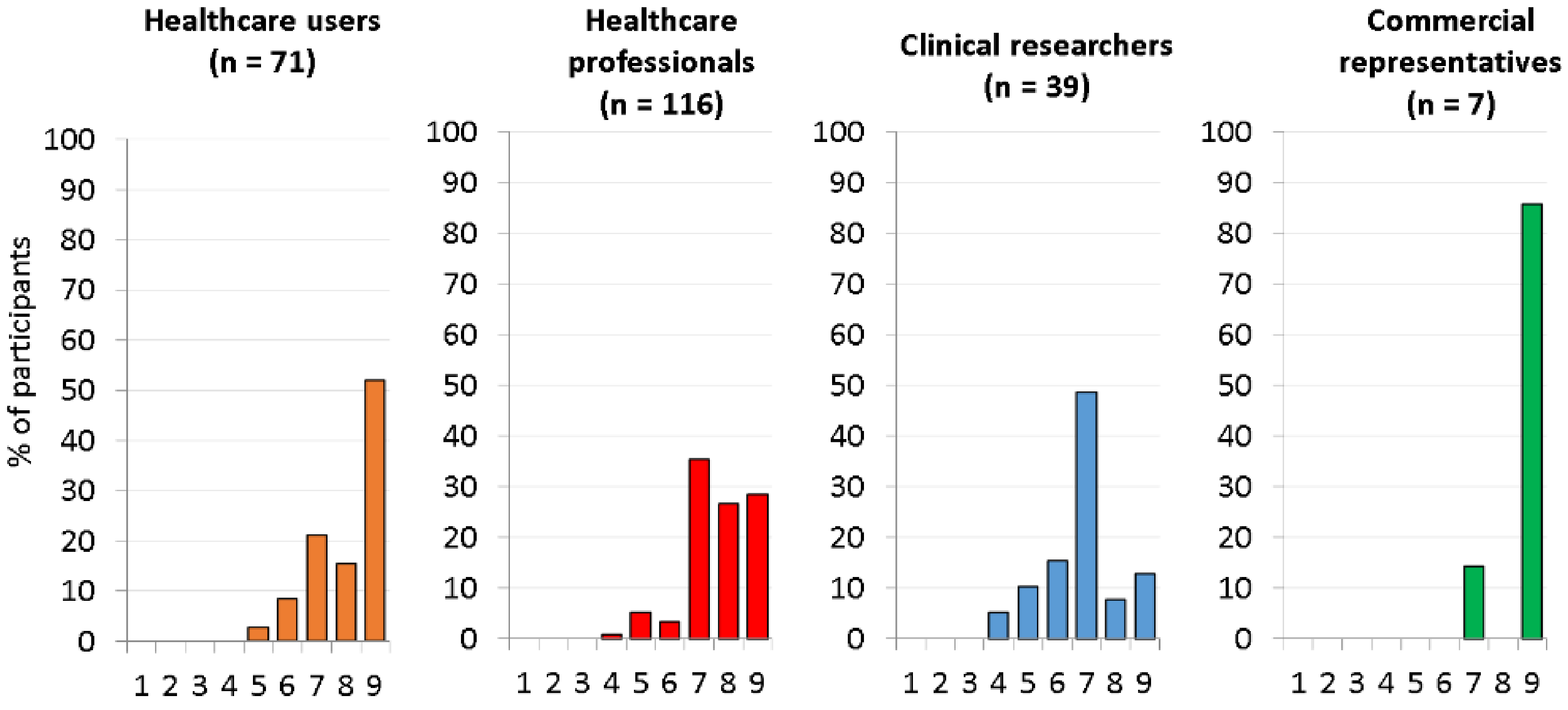

Domain Category:
Sound quality

NIHR | National Institute for Health Research

Rating scale:

1	2	3	4	5	6	7	8	9
Not at all important			*Important but not critical*			*Critical*		

40. TINNITUS AWARENESS

Noticing the sound of tinnitus is there

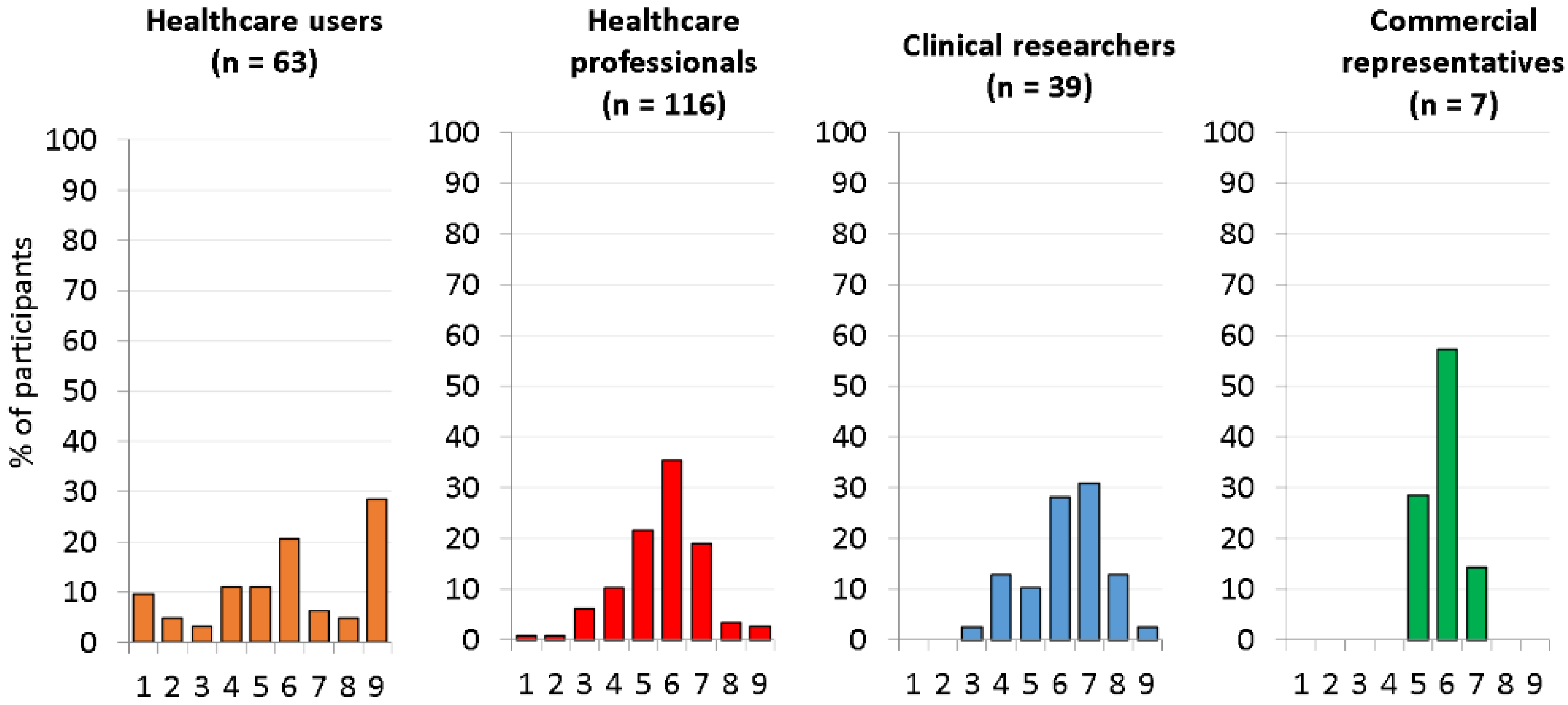

Domain Category: Tinnitus

Rating scale:

1	2	3	4	5	6	7	8	9
Not at all important			*Important but not critical*			*Critical*		

41. TINNITUS INTRUSIVENESS

CROSSSD
CORE REHABILITATION OUTCOME SET for SINGLE SIDED DEAFNESS

Being acutely aware of the sounds of tinnitus; feeling that it is invading your life or your personal space; changing your thoughts or actions and negatively impacting on your life

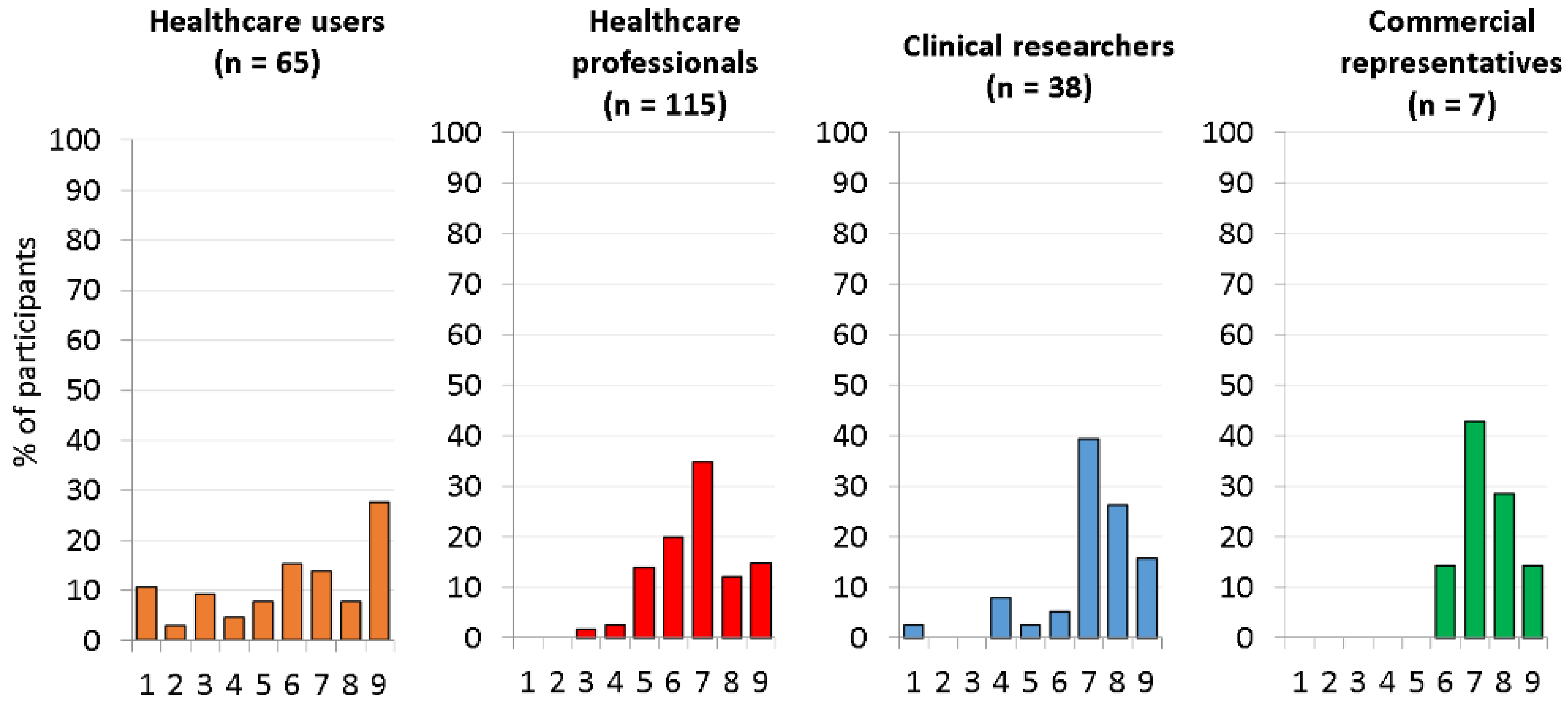

Domain Category: Tinnitus

NIHR | National Institute for Health Research

Rating scale:

1	2	3	4	5	6	7	8	9
Not at all important			*Important but not critical*			*Critical*		

42. TINNITUS LOUDNESS

How loud your tinnitus sounds

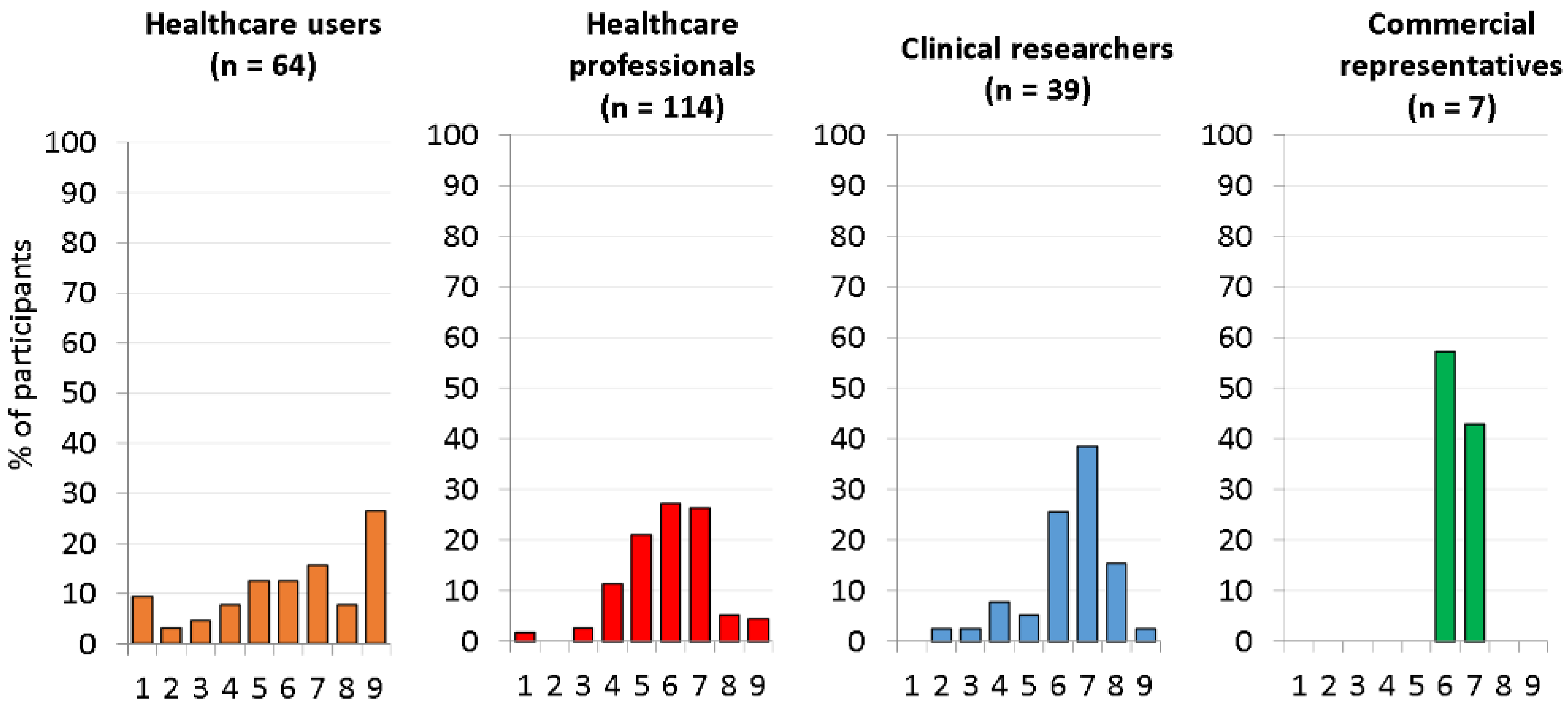

Domain Category: Tinnitus

NIHR | National Institute for Health Research

Rating scale:

1	2	3	4	5	6	7	8	9
Not at all important			*Important but not critical*			*Critical*		

43. TINNITUS PITCH

Whether your tinnitus has a note-like quality;
for example high pitch like whistling or low pitch like humming

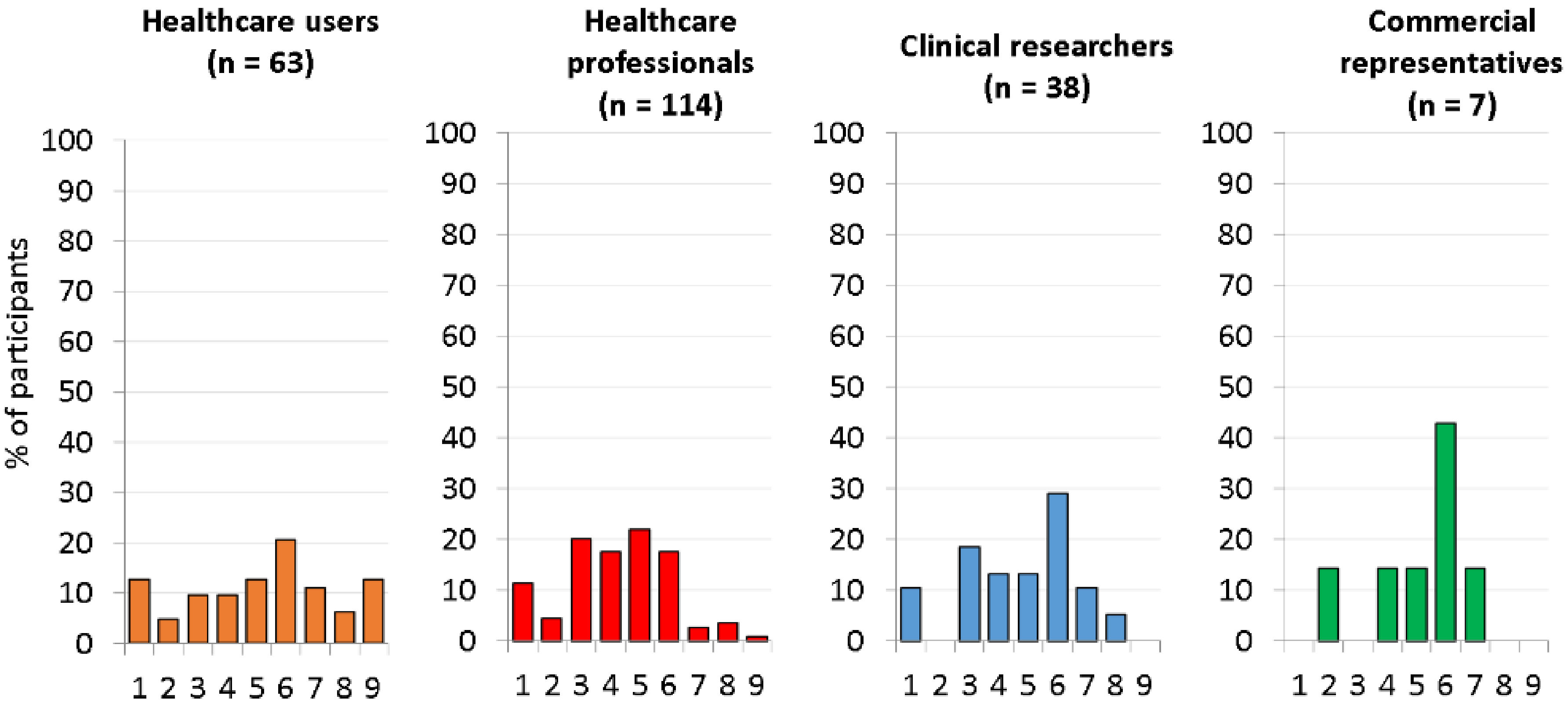

Domain Category: Tinnitus

NIHR | National Institute for Health Research

Rating scale:

1	2	3	4	5	6	7	8	9
Not at all important			*Important but not critical*			*Critical*		

44. TINNITUS QUALITY

What type of sound is heard
(for example; hissing; buzzing; ringing; whistling etc)

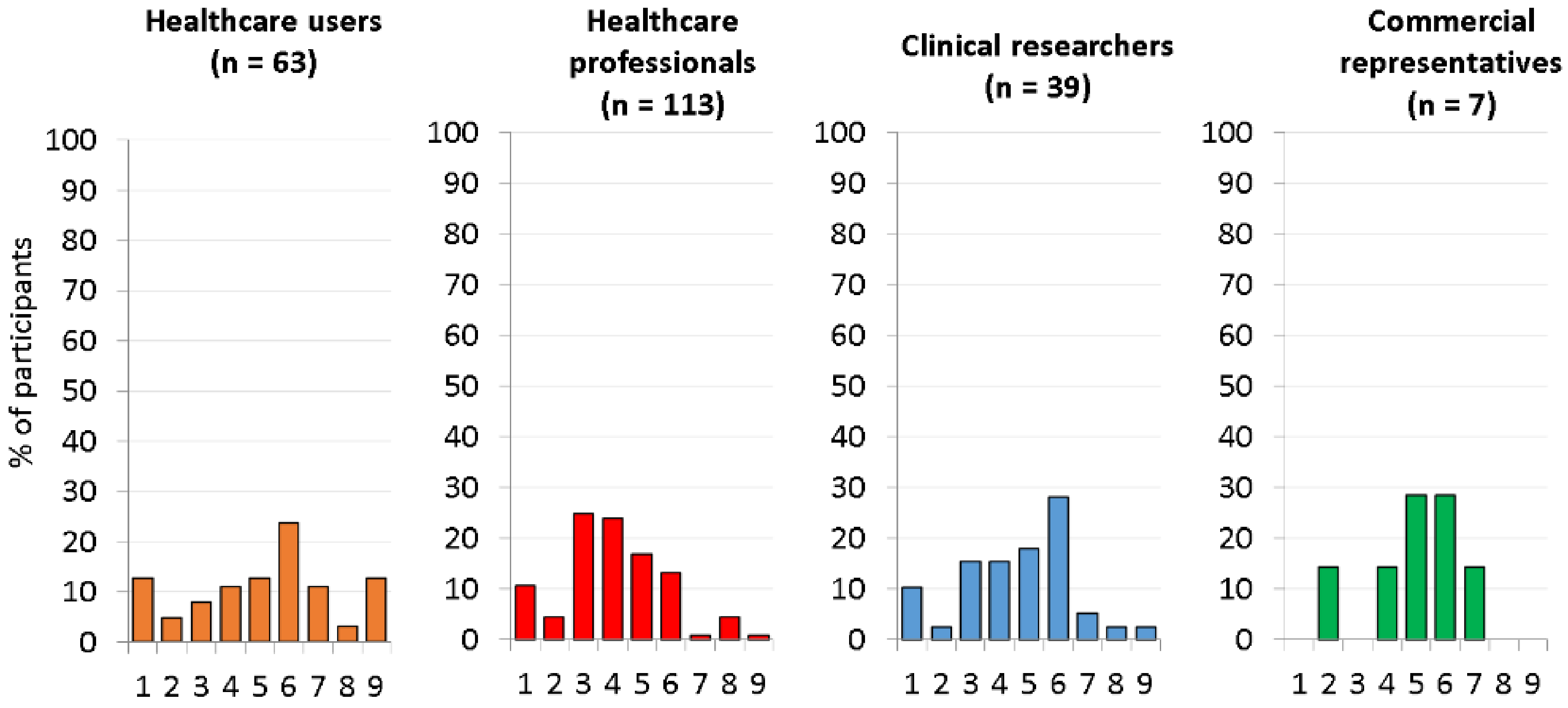

Domain Category: Tinnitus

NIHR | National Institute for Health Research

Rating scale:

1	2	3	4	5	6	7	8	9
Not at all important			*Important but not critical*			*Critical*		

45. DEVICE USABILITY

How easy it is to learn, use, and maintain the device (for example, changing the batteries, cleaning)

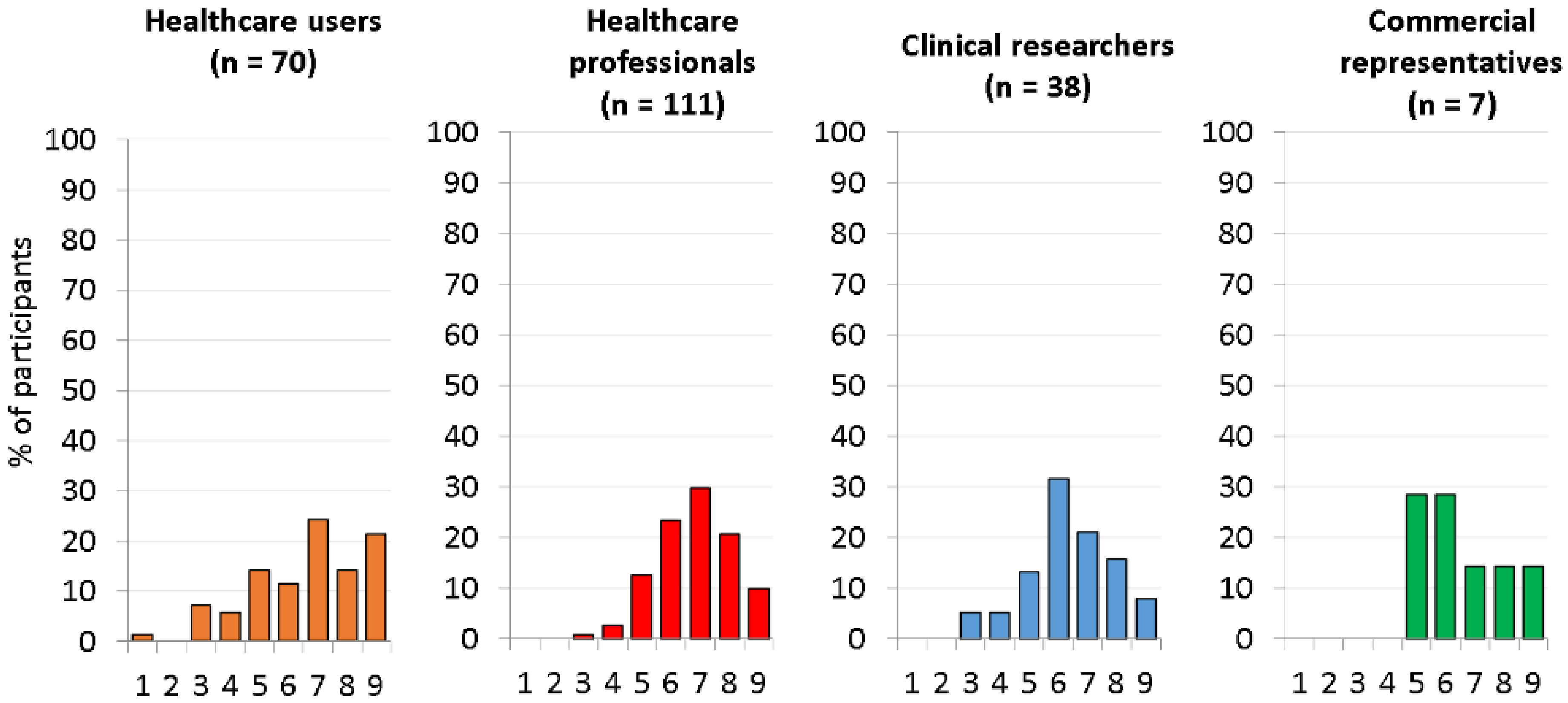

Domain Category: Factors related to the treatment being tested

Rating scale:

1	2	3	4	5	6	7	8	9
Not at all important			*Important but not critical*			*Critical*		

NIHR | National Institute for Health Research

46. IMPACT ON LEARNING

Effect of your hearing loss or device on your ability to acquire new knowledge or skills, or further your education

CROSSSD

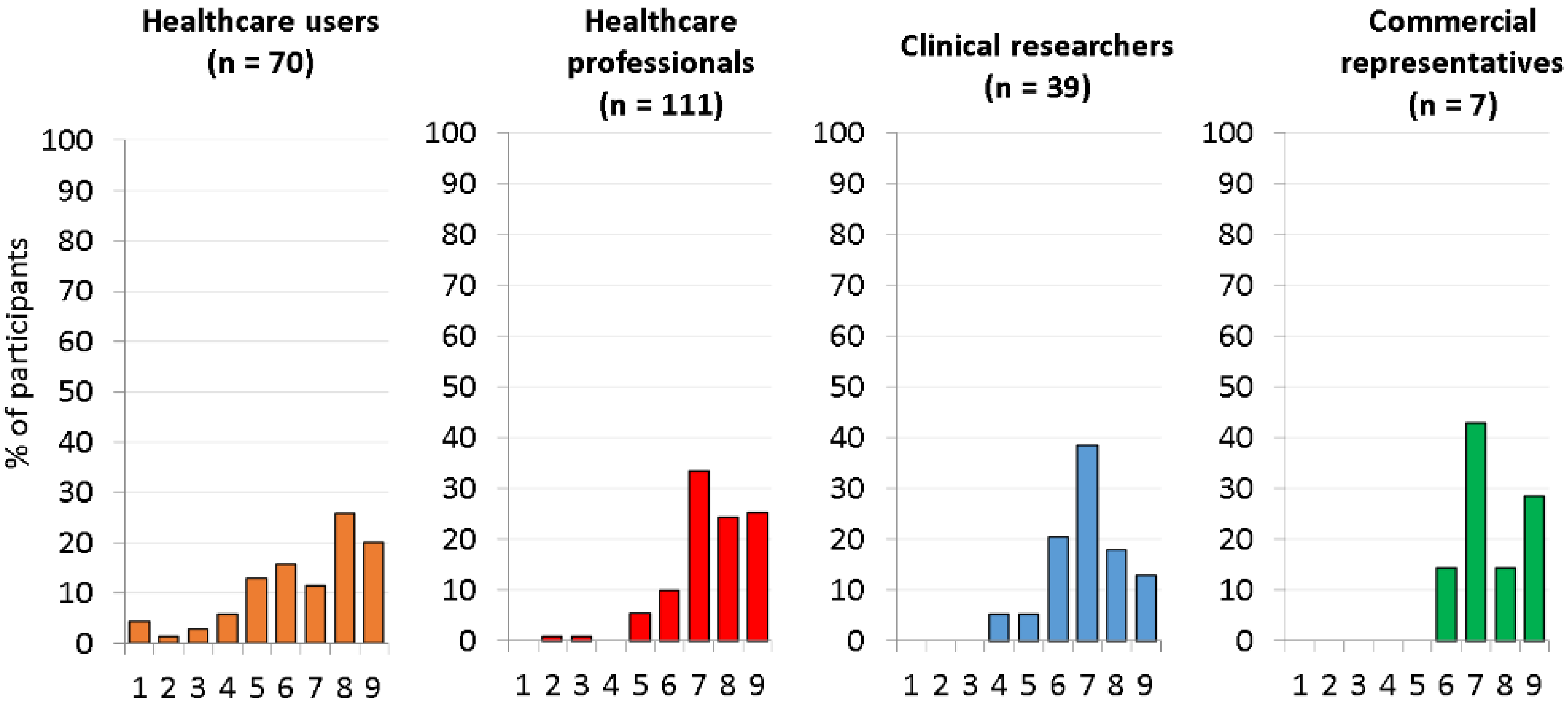

Domain Category: Health-related quality of life

NIHR | National Institute for Health Research

Rating scale:

1	2	3	4	5	6	7	8	9
Not at all important			*Important but not critical*			*Critical*		

47. INDEPENDENCE

How your hearing loss affects how much you need to rely on other people in daily life

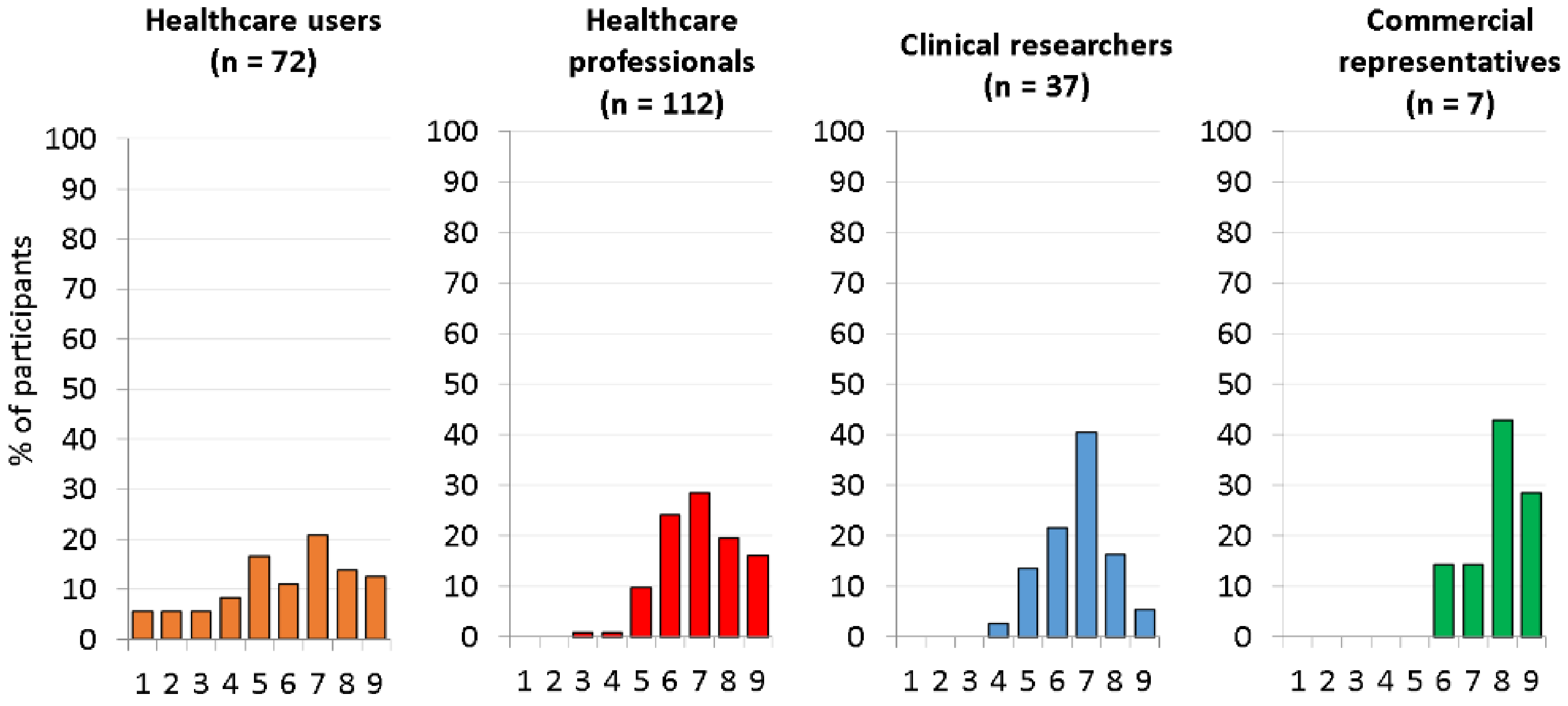

Domain Category: Self

Rating scale:

1	2	3	4	5	6	7	8	9
Not at all important			*Important but not critical*			*Critical*		

NIHR | National Institute for Health Research

48. CONCERN ABOUT YOUR HEARING

Feeling worried about the hearing in your better ear and the thought that it may decline

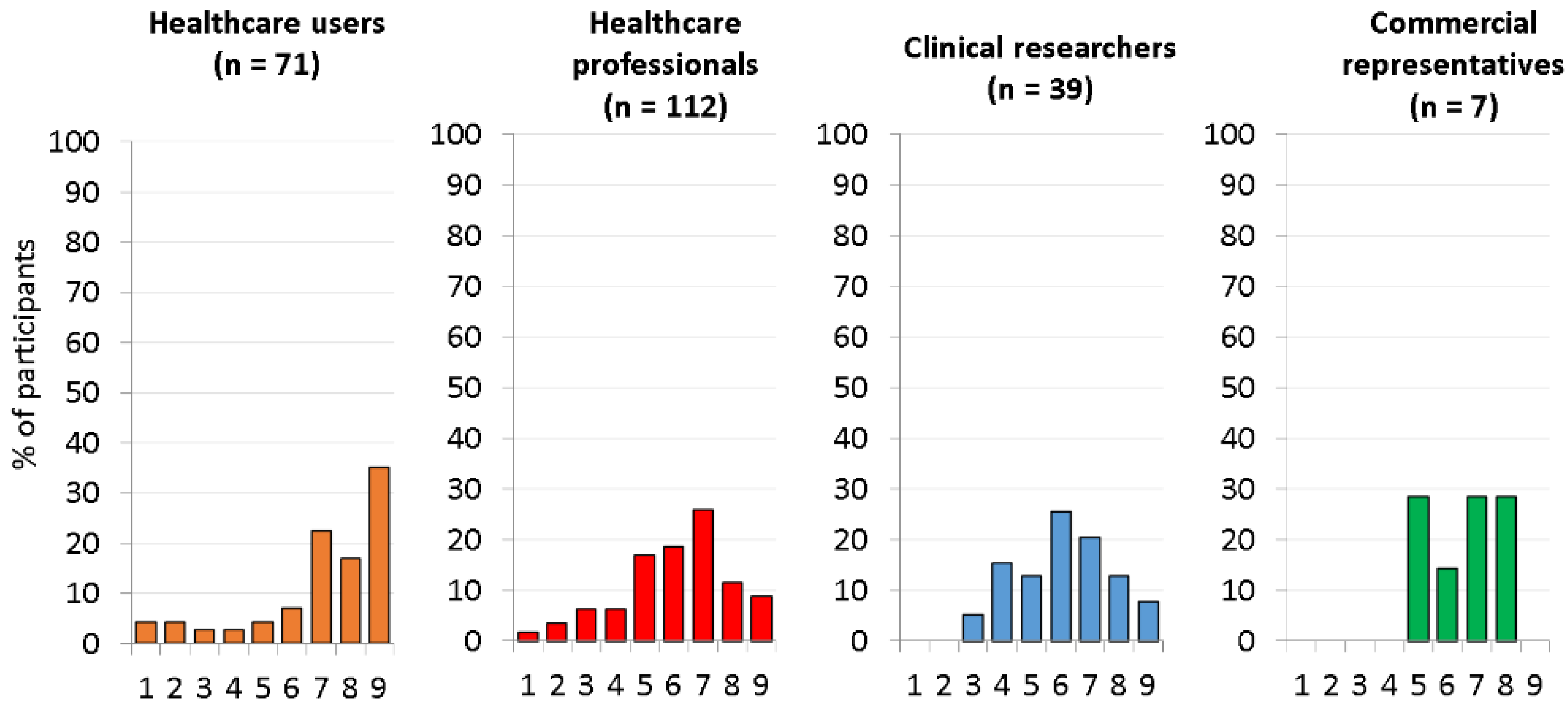

Domain Category:
Psychological effects

NIHR | National Institute for Health Research

Rating scale:

1	2	3	4	5	6	7	8	9
Not at all important			*Important but not critical*			*Critical*		

49. VULNERABILITY

Feeling insecure because your hearing loss affects your awareness of potential hazards and threats in your daily life (for example, moving traffic, hazards at the workplace) and those you may not be able to see or hear (for example, other people behind you)

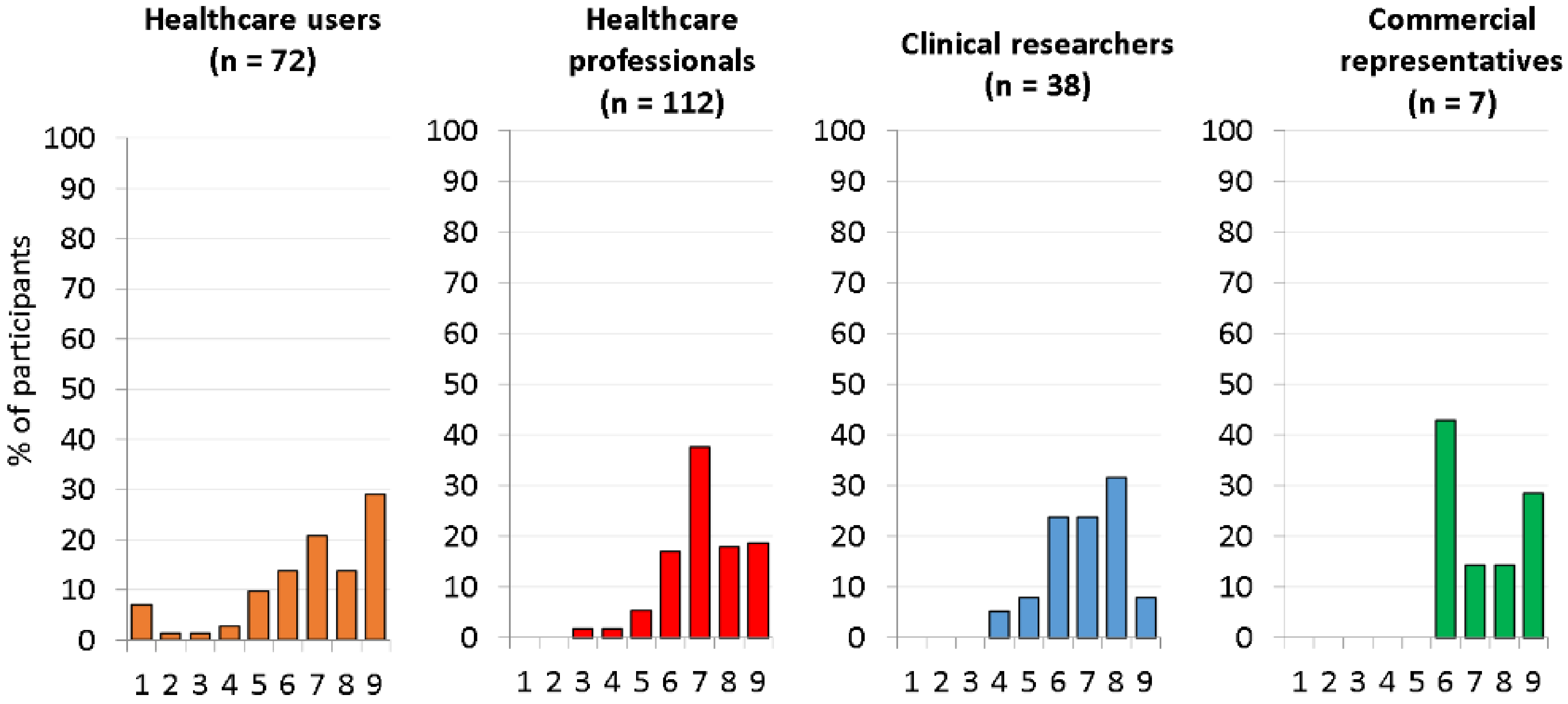

Domain Category: Self

Rating scale:

1	2	3	4	5	6	7	8	9
Not at all important			*Important but not critical*			*Critical*		

National Institute for Health Research
