# Supplementary material for: The Core Rehabilitation Outcome Set for Single-Sided Deafness (CROSSSD) study: International consensus on outcome measures for trials of interventions for adults with single-sided deafness
Source: Trials. 2022 Sep 8;23:764. doi: 10.1186/s13063-022-06702-1 (PMC9454406; doi:10.1186/s13063-022-06702-1)

Additional file 5. Consenting participants' characteristics and demographics.

Table 5a. Number and percentage (%) of participants in each e-Delphi survey.

Stakeholder group	Participated in Round 1 n (%)	Invited to Round 2 (completed >50% of Round 1)	Participated in Round 2 n (%)	Retention rate (%)
Healthcare users	92 (29.9)	81	71 (30.5)	87.7
Healthcare professionals	148 (48.1)	135	116 (49.8)	85.9
Clinical researchers	59 (19.2)	53	39 (16.7)	73.6
Commercial representatives	8 (2.6)	8	7 (3.0)	87.5
Funders	1 (0.3)	0	-	-
**Total (n)**	**308**	**277**	**233**	**75.6**

Table 5b. Age range of all (n=308) participants who consented to take part in the e-Delphi survey, arranged by stakeholder group.

Stakeholder group	18-29 years	30-39 years	40-49 years	50-59 years	60-69 years	70-79 years	80-89 years	Total
Healthcare users	7	15	18	17	21	12	2	92
Healthcare professionals	19	61	39	24	5	-	-	148
Clinical researchers	4	17	17	13	8	-	-	59
Commercial representatives	1	5	2	-	-	-	-	8
Funders	-	-	1	-	-	-	-	1
**Total n (%)**	**31 (10.1)**	**98 (31.8)**	**77 (25.0)**	**54 (17.5)**	**34 (11.0)**	**12 (3.9)**	**2 (0.6)**	**308 (100)**

Table 5c. Distribution of consenting participants (n=308) across countries (n=29) arranged per stakeholder group.

Country	Healthcare users	Healthcare professionals	Clinical researchers	Commercial rep-resentatives	Funders	Grand total per country	%
Argentina		2	2			4	1.3
Australia		9	2			11	3.6
Austria			2			2	0.6
Belgium		1	2			3	1.0
Canada	2		1			3	1.0
China		1	1	1		3	1.0
Cyprus		2				2	0.6
Czechia		1				1	0.3
Denmark			1	1		2	0.6
Estonia	1	3				4	1.3
France			1			1	0.3
Germany	1	4	5			10	3.2
India	1	4	1			6	1.9
Ireland	14	21	2			37	12.0
Italy			2			2	0.6
Japan		1				1	0.3
Jordan		1				1	0.3

Malaysia		4				4	1.3
Netherlands		4	4			8	2.6
New Zealand		5				5	1.6
Norway			1			1	0.3
Poland			3			3	1.0
South Korea			1			1	0.3
Spain	3	2				5	1.6
Sweden		1	1			2	0.6
Switzerland			3			3	1.0
Turkey			1			1	0.3
United Kingdom	67	63	8	6	1	145	47.1
United States of America	3	19	15			37	12.0
**Total n (%)**	**92 (29.9)**	**148 (48.0)**	**59 (19.1)**	**8 (2.6)**	**1 (0.3)**	**308**	

Table 5d. Primary language for everyday communication disclosed by consenting participants (n=308) across countries (n=29) arranged per stakeholder group. A variety of 25 different languages were listed. One healthcare professional did not disclose their primary language of communication.

**Primary language**	**Healthcare users**	**Healthcare professionals**	**Clinical researchers**	**Commercial rep-resentatives**	**Funders**	**Total n of participants**	**%**
English	88	117	31	6	1	243	78.9
German	1	4	10			15	4.9
Dutch		5	6			11	3.6
Spanish		4	2			6	1.9
Estonian	1	3				4	1.3
Polish			3			3	1.0
Swedish		1	2			3	1.0
French	1		1			2	0.6
Greek		2				2	0.6
Italian			2			2	0.6
Malay		2				2	0.6
Afrikaans		1				1	0.3
Arabic		1				1	0.3
Bahasa Melayu		1				1	0.3
Cantonese		1				1	0.3
Czech		1				1	0.3
Danish				1		1	0.3
Hindi	1					1	0.3
Japanese		1				1	0.3
Kannada		1				1	0.3
Korean			1			1	0.3
Mandarin				1		1	0.3
Marathi		1				1	0.3
Turkish			1			1	0.3
Welsh		1				1	0.3
Not stated		1				1	0.3

**Total n**	**92**	**148**	**59**	**8**	**1**	**308**	

Figure 5e. Time since SSD diagnosis as disclosed by healthcare users (n=84). For the majority, it had been 2-5 years since their diagnosis of SSD. Healthcare users needed to have lived experience of SSD for 12 months or more to be eligible to take part.

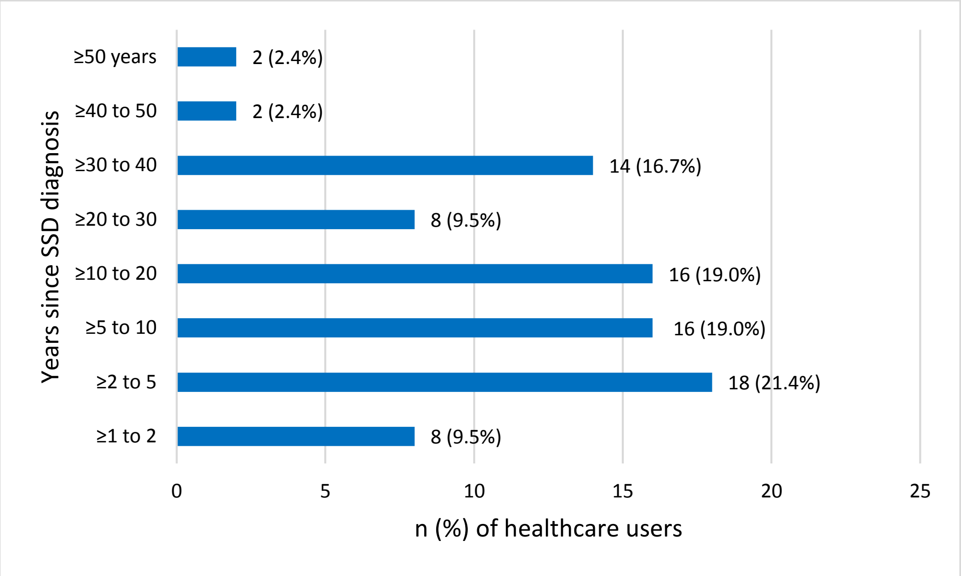

Supplement: Supplementary file 5 — Additional file 5. Consenting participants’ characteristics and demographics [file 13063_2022_6702_MOESM5_ESM.pdf]
