# Supplementary material for: The Core Rehabilitation Outcome Set for Single-Sided Deafness (CROSSSD) study: International consensus on outcome measures for trials of interventions for adults with single-sided deafness
Source: Trials. 2022 Sep 8;23:764. doi: 10.1186/s13063-022-06702-1 (PMC9454406; doi:10.1186/s13063-022-06702-1)
Supplement: Supplementary file 7 — Additional file 7. Detailed breakdown of the participant feedback [file 13063_2022_6702_MOESM7_ESM.pdf]

Additional file 7. Breakdown of responses received by 95 (43.3%) participants out of 219 who completed both rounds of the e-Delphi survey. Participants were asked to rate on a 5-point rating scale how satisfied they were with the choice of outcome domains included in the core outcome domains set.

**Stakeholder group**	**Respondents n (%)**	***very satisfied* n (%)**	***somewhat satisfied* n (%)**	***neither satisfied or dissatisfied* n (%)**	***somewhat dissatisfied* n (%)**	***I am very dissatisfied* n (%)**
Healthcare users	32 (33.7)	25 (78.1)	6 (18.8)	-	-	1 (3.1%)
Healthcare professionals	48 (50.5)	38 (79.2)	8 (16.7)	1 (2.1)	-	1 (2.1%)
Clinical researchers	14 (14.7)	9 (64.3)	5 (35.7)	-	-	-
Commercial representatives	1 (1.0)	1 (100)	-	-	-	-
**Total n (%)**	**95**	**73 (76.8)**	**19 (20)**	**1 (1.1)**	**-**	**2 (2.1)**
